# Supplementary material for: Preserved collagen reveals species identity in archaeological marine turtle bones from Caribbean and Florida sites
Source: R Soc Open Sci. 2019 Oct 30;6(10):191137. doi: 10.1098/rsos.191137 (PMC6837194; doi:10.1098/rsos.191137)
Supplement: Supplementaries [file rsos191137supp1.pdf]

SUPPLEMENTARY INFORMATION FOR:

Preserved collagen reveals species identity in archaeological marine turtle bones from Caribbean and Florida sites

Virginia L. Harvey[1], Michelle J. LeFebvre[2], Susan deFrance[3], Casper Toftgaard[4,5], Konstantina Drosou[6], Andrew C. Kitchener[7] and Michael Buckley[1*]*

[1]*Manchester Institute of Biotechnology,* School of Earth and Environmental Sciences*, 131 Princess Street, University of Manchester. M1 7DN. UK*

[2]*Florida Museum of Natural History, University of Florida, Gainesville, FL 32611, United States*

[3]*Department of Anthropology, University of Florida, Gainesville, FL 32611, United States*

[4]*The SAXO Institute, University of Copenhagen, Karen Blixens Plads 8, 2300 København S*

[5]*National Museum of Denmark, Nationalmuseet, Ny Vestergade 10, Prinsens Palæ, DK-1471, København K*

[6]*KNH Centre for Biomedical Egyptology, School of Biological Sciences, 99 Oxford Road, Manchester. M13 9PG, UK*

[7]*Department of Natural Sciences, National Museums Scotland, Chambers Street, Edinburgh EH1 1JF, UK and The University of Edinburgh, Institute of Geography, School of Geosciences, Drummond Street, Edinburgh, EH8 9XP, UK*

**Correspondence to virginia.harvey@manchester.ac.uk and m.buckley@manchester.ac.uk*

Royal Society Open Science. Accepted: 3rd September 2019.

Supplementary Table S1: Full list of all archaeological specimens in this study. (1) and (2) represent the two different genetic stocks proposed for *Chelonia* spp.

No.	Site	Origin	FS Lot #:	Provenience /Unit	Square	Level/ Layer	Depth (cm)	Morphological ID	Element	NISP	Cat No.	ZooMS Sample Code	ZooMS Identification
1	Garden Patch	Florida, USA	4	Area X, TU 1	–	D	30–40	Cheloniidae, cf. *Caretta caretta*	unidentified fragment	1	FLMNH ANT 2013-22-4.29	GP1	*Chelonia mydas* (1)
2	Garden Patch	Florida, USA	4	Area X, TU 1	–	D	30–40	Cheloniidae, cf. *Caretta caretta*	post cranial fragment	1	FLMNH ANT 2013-22-4.29	GP2	*Chelonia mydas* (1)
3	Garden Patch	Florida, USA	5	Area X, TU 1	–	E	40–50	Cheloniidae	post cranial fragment	1	FLMNH ANT 2013-22-5.25	GP3	Chelydridae
4	Garden Patch	Florida, USA	5	Area X, TU 1	–	E	40–50	Cheloniidae	marginal fragment	1	FLMNH ANT 2013-22-5.25	GP4	*Chelonia mydas* (1)
5	Garden Patch	Florida, USA	5	Area X, TU 1	–	E	40–50	Cheloniidae	marginal fragment	1	FLMNH ANT 2013-22-5.25	GP5	POOR
6	Garden Patch	Florida, USA	5	Area X, TU 1	–	E	40–50	Cheloniidae	marginal fragment	1	FLMNH ANT 2013-22-5.25	GP6	*Chelonia mydas* (1)
7	Garden Patch	Florida, USA	5	Area X, TU 1	–	E	40–50	Cheloniidae	carapace/plastron fragment	1	FLMNH ANT 2013-22-5.25	GP7	POOR
8	Garden Patch	Florida, USA	19	Area X, TU 1	–	G	60–70	Cheloniidae, cf. *Caretta caretta*	humerus, distal fragment	1	FLMNH ANT 2013-22-19.18	GP8	*Chelonia mydas* (1)
9	Garden Patch	Florida, USA	19	Area X, TU 1	–	G	60–70	Cheloniidae, cf. *Caretta caretta*	post cranial fragment	1	FLMNH ANT 2013-22-19.18	GP9	*Chelonia* sp. (2)
10	Garden Patch	Florida, USA	19	Area X, TU 1	–	G	60–70	Cheloniidae, cf. *Caretta caretta*	cf. forelimb	1	FLMNH ANT 2013-22-19.18	GP10	*Chelonia mydas* (1)
11	Garden Patch	Florida, USA	19	Area X, TU 1	–	G	60–70	Cheloniidae, cf. *Caretta caretta*	scapula	1	FLMNH ANT 2013-22-19.18	GP11	*Chelonia mydas* (1)
12	Garden Patch	Florida, USA	19	Area X, TU 1	–	G	60–70	Cheloniidae, cf. *Caretta caretta*	carapace/plastron fragment	1	FLMNH ANT 2013-22-19.18	GP12	*Chelonia mydas* (1)
13	Garden Patch	Florida, USA	19	Area X, TU 1	–	G	60–70	Cheloniidae, cf. *Caretta caretta*	humerus, distal fragment	1	FLMNH ANT 2013-22-19.18	GP13	*Chelonia mydas* (1)
14	Garden Patch	Florida, USA	19	Area X, TU 1	–	G	60–70	Cheloniidae, cf. *Caretta caretta*	carapace/plastron fragment	1	FLMNH ANT 2013-22-19.18	GP14	*Chelonia mydas* (1)
15	Garden Patch	Florida, USA	19	Area X, TU 1	–	G	60–70	Cheloniidae, cf. *Caretta caretta*	post cranial fragment	1	FLMNH ANT 2013-22-19.18	GP15	POOR
16	Garden Patch	Florida, USA	19	Area X, TU 1	–	G	60–70	Cheloniidae, cf. *Caretta caretta*	post cranial fragment	1	FLMNH ANT 2013-22-19.18	GP16	POOR
17	Garden Patch	Florida, USA	19	Area X, TU 1	–	G	60–70	Cheloniidae, cf. *Caretta caretta*	marginal fragment	1	FLMNH ANT 2013-22-19.18	GP17	*Chelonia mydas* (1)
18	Garden Patch	Florida, USA	29	Mound II, TU 2	–	C	20–30	Cheloniidae	distal humerus or femur fragment	1	FLMNH ANT 2013-22-29.20	GP18	*Lepidochelys* spp.
19	Garden Patch	Florida, USA	29	Mound II, TU 2	–	C	20–30	Cheloniidae	carapace fragment	1	FLMNH ANT 2013-22-29.20	GP19	*Lepidochelys* spp.
20	Garden Patch	Florida, USA	29	Mound II, TU 2	–	C	20–30	Cheloniidae	marginal fragment	1	FLMNH ANT 2013-22-29.20	GP20	*Chelonia* sp. (2)
21	Garden Patch	Florida, USA	29	Mound II, TU 2	–	C	20–30	Cheloniidae	carapace, cf. neural fragment	1	FLMNH ANT 2013-22-29.20	GP21	*Chelonia mydas* (1)
22	Garden Patch	Florida, USA	29	Mound II, TU 2	–	C	20–30	Cheloniidae	cf. cranial fragment	1	FLMNH ANT 2013-22-29.20	GP22	*Chelonia mydas* (1)
23	Garden Patch	Florida, USA	36	Area X, TU 1	–	I	80–90	Cheloniidae	cf. pectoral girdle fragment	1	FLMNH ANT 2013-22-36.12	GP23	*Chelonia mydas* (1)
24	Garden Patch	Florida, USA	36	Area X, TU 1	–	I	80–90	Cheloniidae	marginal fragment	1	FLMNH ANT 2013-22-36.12	GP24	*Chelonia mydas* (1)
25	Garden Patch	Florida, USA	36	Area X, TU 1	–	I	80–90	Cheloniidae	marginal fragment	1	FLMNH ANT 2013-22-36.12	GP25	*Chelonia mydas* (1)
26	Garden Patch	Florida, USA	36	Area X, TU 1	–	I	80–90	Cheloniidae	carapace/plastron fragment	1	FLMNH ANT 2013-22-36.12	GP26	Emydidae
27	Garden Patch	Florida, USA	36	Area X, TU 1	–	I	80–90	Cheloniidae	marginal fragment	1	FLMNH ANT 2013-22-36.12	GP27	*Chelonia mydas* (1)
28	Garden Patch	Florida, USA	57	Mound II, TU 2	–	F	50–60	Cheloniidae	carapace/plastron fragment	1	FLMNH ANT 2013-22-57.17	GP28	*Chelonia mydas* (1)
29	Garden Patch	Florida, USA	57	Mound II, TU 2	–	F	50–60	Cheloniidae	marginal fragment	1	FLMNH ANT 2013-22-57.17	GP29	POOR
30	Garden Patch	Florida, USA	57	Mound II, TU 2	–	F	50–60	Cheloniidae	left femur	1	FLMNH ANT 2013-22-57.17	GP30	*Chelonia mydas* (1)
31	Garden Patch	Florida, USA	57	Mound II, TU 2	–	F	50–60	Cheloniidae	cf. pectoral girdle fragment	1	FLMNH ANT 2013-22-57.17	GP31	*Chelonia mydas* (1)
32	Garden Patch	Florida, USA	57	Mound II, TU 2	–	F	50–60	Cheloniidae	carapace fragment	1	FLMNH ANT 2013-22-57.17	GP32	POOR
33	Garden Patch	Florida, USA	76	Mound II, TU 2	–	I	80–90	Cheloniidae	carapace fragment	1	FLMNH ANT 2013-22-76.24	GP33	*Lepidochelys* spp.
34	Garden Patch	Florida, USA	76	Mound II, TU 2	–	I	80–90	Cheloniidae	carapace fragment	1	FLMNH ANT 2013-22-76.24	GP34	*Chelonia mydas* (1)
35	Garden Patch	Florida, USA	76	Mound II, TU 2	–	I	80–90	Cheloniidae	carapace fragment	1	FLMNH ANT 2013-22-76.24	GP35	*Chelonia mydas* (1)
36	Garden Patch	Florida, USA	76	Mound II, TU 2	–	I	80–90	Cheloniidae	carapace/plastron fragment	1	FLMNH ANT 2013-22-76.24	GP36	*Chelonia mydas* (1)
37	Garden Patch	Florida, USA	76	Mound II, TU 2	–	I	80–90	Cheloniidae	carapace/plastron fragment	1	FLMNH ANT 2013-22-76.24	GP37	*Chelonia mydas* (1)
38	Garden Patch	Florida, USA	76	Mound II, TU 2	–	I	80–90	Cheloniidae	marginal fragment	1	FLMNH ANT 2013-22-76.24	GP38	*Chelonia mydas* (1)
39	Garden Patch	Florida, USA	76	Mound II, TU 2	–	I	80–90	Cheloniidae	carapace fragment	1	FLMNH ANT 2013-22-76.24	GP39	Testudinidae
40	Garden Patch	Florida, USA	76	Mound II, TU 2	–	I	80–90	Cheloniidae	carapace/plastron fragment	1	FLMNH ANT 2013-22-76.24	GP40	Testudinidae
41	Garden Patch	Florida, USA	76	Mound II, TU 2	–	I	80–90	Cheloniidae	marginal fragment	1	FLMNH ANT 2013-22-76.24	GP41	*Chelonia mydas* (1)
42	Garden Patch	Florida, USA	76	Mound II, TU 2	–	I	80–90	Cheloniidae	carapace fragment	1	FLMNH ANT 2013-22-76.24	GP42	*Chelonia mydas* (1)
43	Garden Patch	Florida, USA	159	Mound II, TU 2	–	L	110–120	Cheloniidae	carapace fragment	1	FLMNH ANT 2013-22-159.22	GP43	*Lepidochelys* spp.
44	Garden Patch	Florida, USA	159	Mound II, TU 2	–	L	110–120	Cheloniidae	unidentified fragment, possibly cranial	1	FLMNH ANT 2013-22-159.22	GP44	*Chelonia mydas* (1)
45	Garden Patch	Florida, USA	159	Mound II, TU 2	–	L	110–120	Cheloniidae	marginal fragment	1	FLMNH ANT 2013-22-159.22	GP45	*Chelonia mydas* (1)
46	Garden Patch	Florida, USA	159	Mound II, TU 2	–	L	110–120	Cheloniidae	unidentified fragment, possibly cranial	1	FLMNH ANT 2013-22-159.22	GP46	*Chelonia mydas* (1)
47	Garden Patch	Florida, USA	159	Mound II, TU 2	–	L	110–120	Cheloniidae	pectoral girdle fragment	1	FLMNH ANT 2013-22-159.22	GP47	*Chelonia mydas* (1)
48	Garden Patch	Florida, USA	159	Mound II, TU 2	–	L	110–120	Cheloniidae	post cranial fragment	1	FLMNH ANT 2013-22-159.22	GP48	*Chelonia mydas* (1)
49	Garden Patch	Florida, USA	159	Mound II, TU 2	–	L	110–120	Cheloniidae	marginal fragment	1	FLMNH ANT 2013-22-159.22	GP49	*Chelonia mydas* (1)
50	Garden Patch	Florida, USA	159	Mound II, TU 2	–	L	110–120	Cheloniidae	carapace fragment	1	FLMNH ANT 2013-22-159.22	GP50	POOR
51	Garden Patch	Florida, USA	159	Mound II, TU 2	–	L	110–120	Cheloniidae	flipper element	1	FLMNH ANT 2013-22-159.22	GP51	*Chelonia mydas* (1)
52	Garden Patch	Florida, USA	159	Mound II, TU 2	–	L	110–120	Cheloniidae	carapace fragment	1	FLMNH ANT 2013-22-159.22	GP52	*Chelonia mydas* (1)
53	Garden Patch	Florida, USA	172	Mound II, TU 2	–	P	150–160	Cheloniidae	unidentified element	1	FLMNH ANT 2013-22-172.27	GP53	POOR
54	Garden Patch	Florida, USA	172	Mound II, TU 2	–	P	150–160	Cheloniidae	marginal fragment	1	FLMNH ANT 2013-22-172.27	GP54	*Chelonia mydas* (1)
55	Garden Patch	Florida, USA	172	Mound II, TU 2	–	P	150–160	Cheloniidae	carapace fragment, cf. neural	1	FLMNH ANT 2013-22-172.27	GP55	*Chelonia mydas* (1)

56	Garden Patch	Florida, USA	172	Mound II, TU 2	–	P	150–160	Cheloniidae	marginal fragment	1	FLMNH ANT 2013-22-172.27	GP56	*Chelonia* sp. (2)
57	Garden Patch	Florida, USA	172	Mound II, TU 2	–	P	150–160	Cheloniidae	carapace fragment, cf. neural	1	FLMNH ANT 2013-22-172.27	GP57	*Chelonia mydas* (1)
58	Garden Patch	Florida, USA	172	Mound II, TU 2	–	P	150–160	Cheloniidae	marginal fragment	1	FLMNH ANT 2013-22-172.27	GP58	*Chelonia mydas* (1)
59	Garden Patch	Florida, USA	172	Mound II, TU 2	–	P	150–160	Cheloniidae	carapace fragment	1	FLMNH ANT 2013-22-172.27	GP59	*Chelonia mydas* (1)
60	Garden Patch	Florida, USA	172	Mound II, TU 2	–	P	150–160	Cheloniidae	carapace fragment	1	FLMNH ANT 2013-22-172.27	GP60	*Chelonia mydas* (1)
61	Garden Patch	Florida, USA	172	Mound II, TU 2	–	P	150–160	Cheloniidae	carapace/plastron fragment	1	FLMNH ANT 2013-22-172.27	GP61	*Chelonia mydas* (1)
62	Garden Patch	Florida, USA	172	Mound II, TU 2	–	P	150–160	Cheloniidae	carapace/plastron fragment	1	FLMNH ANT 2013-22-172.27	GP62	POOR
63	Grand Bay	Carriacou, Grenadines	04CGB000371BOA	Trench 447, Square 7	–	2	–	Cheloniidae	sample from humerus, left, proximal 1/2	2	–	GB63	*Chelonia mydas* (1)
64	Grand Bay	Carriacou, Grenadines	05CGB000612BOA	Trench 446, Square 12	–	4	–	Cheloniidae	sample from humerus, left, distal 1/3	4	–	GB64	*Chelonia mydas* (1)
65	Grand Bay	Carriacou, Grenadines	04CGB000241	Trench 446, Square 21	–	2	–	Cheloniidae	sample from plastron	3	–	GB65	*Chelonia mydas* (1)
66	Grand Bay	Carriacou, Grenadines	04CBG000371BOA	Trench 447, Square 7	–	2	–	Cheloniidae	sample from humerus, left, complete	2	–	GB66	*Chelonia mydas* (1)
67	Grand Bay	Carriacou, Grenadines	04CBG000237	Trench 446, Square 17	–	2	–	Cheloniidae, cf. *Chelonia mydas*	femur, right, proximal 2/3	1	–	GB67	POOR
68	Grand Bay	Carriacou, Grenadines	04CGB000298	Trench 446, Square 18	–	2	–	Cheloniidae, cf. *Chelonia mydas*	humerus, proximal head fragment	1	–	GB68	*Chelonia mydas* (1)
69	Grand Bay	Carriacou, Grenadines	04CGB000240	Trench 446, Square 20	–	2	–	Cheloniidae, cf. *Eretmochelys* sp.	femur, left, distal 3/4 complete	1	–	GB69	*Eretmochelys imbricata*
70	Grand Bay	Carriacou, Grenadines	04CGB000240	Trench 446, Square 20	–	2	–	Cheloniidae, cf. *Eretmochelys* sp.	radius, right, complete	1	–	GB70	*Eretmochelys imbricata*
71	Grand Bay	Carriacou, Grenadines	04CGB000_	Trench 446, Square 21	–	2	–	Cheloniidae, cf. *Eretmochelys* sp.	opisthotic, nearly whole	1	–	GB71	*Eretmochelys imbricata*
72	Grand Bay	Carriacou, Grenadines	05CGB000612BOA	Trench 446, Square 12	–	4	–	Cheloniidae	sample from humerus, right, proximal 1/2	1	–	GB72	*Chelonia* sp. (2)
73	Grand Bay	Carriacou, Grenadines	05CGB000612BOA	Trench 446, Square 12	–	4	–	Cheloniidae	sample from carapace, marginal	1	–	GB73	*Eretmochelys imbricata*
74	MC-6	Middle Caicos, Turks & Caicos Islands	46	Structure IV	–	5	–	Cheloniidae	carapace fragment (possible marginal)	1	–	MC74	POOR
75	MC-6	Middle Caicos, Turks & Caicos Islands	47	Structure IV	–	3	–	Cheloniidae	post cranial fragment	1	–	MC75	*Chelonia mydas* (1)
76	MC-6	Middle Caicos, Turks & Caicos Islands	54	Structure IV	–	5	–	Cheloniidae	unidentified	1	–	MC85	*Chelonia mydas* (1)
77	MC-6	Middle Caicos, Turks & Caicos Islands	54	Structure IV	–	5	–	Cheloniidae	unidentified	1	–	MC86	*Chelonia mydas* (1)
78	Magens Bay	St. Thomas, US Virgin Islands	0.1.951	1	VI	–	–	Suspected Cheloniidae	suspected carapace/plastron fragment	1	–	T1	*Eretmochelys imbricata*
79	Magens Bay	St. Thomas, US Virgin Islands	0.1.951	1	VI	–	–	Suspected Cheloniidae	suspected carapace/plastron fragment	1	–	T2	*Eretmochelys imbricata*
80	Magens Bay	St. Thomas, US Virgin Islands	0.1.951	1	VI	–	–	Suspected Cheloniidae	suspected carapace/plastron fragment	1	–	T3	*Eretmochelys imbricata*
81	Magens Bay	St. Thomas, US Virgin Islands	0.1.951	1	VI	–	–	Suspected Cheloniidae	suspected carapace/plastron fragment	1	–	T5	*Chelonia mydas* (1)
82	Magens Bay	St. Thomas, US Virgin Islands	0.1.886	1	LI	1	0–30	Suspected Cheloniidae	suspected carapace/plastron fragment	1	–	T6	*Eretmochelys imbricata*
83	Magens Bay	St. Thomas, US Virgin Islands	0.1.951	1	VI	–	–	Suspected Cheloniidae	suspected femur	1	–	T7	*Eretmochelys imbricata*
84	Magens Bay	St. Thomas, US Virgin Islands	0.1.951	1	VI	–	–	Suspected Cheloniidae	unidenfitied long bone	1	–	T8	POOR
85	Magens Bay	St. Thomas, US Virgin Islands	0.1.804	1	VI	4	90–125	Suspected Cheloniidae	suspected carapace/plastron fragment	1	–	T9	*Chelonia mydas* (1)
86	Magens Bay	St. Thomas, US Virgin Islands	0.1.862	1	XXIII	2	30–60	Suspected Cheloniidae	suspected carapace/plastron fragment	1	–	T10	*Chelonia mydas* (1)
87	Magens Bay	St. Thomas, US Virgin Islands	0.1.862	1	XXIII	2	30–60	Suspected Cheloniidae	unidenfitied long bone	1	–	T11	*Chelonia mydas* (1)
88	Magens Bay	St. Thomas, US Virgin Islands	0.1.952	1	VII	1	0–60	Suspected Cheloniidae	suspected carapace/plastron fragment	1	–	T13	*Eretmochelys imbricata*
89	Magens Bay	St. Thomas, US Virgin Islands	0.1.952	1	VII	1	0–60	Suspected Cheloniidae	unidenfitied long bone	1	–	T14	*Eretmochelys imbricata*
90	Magens Bay	St. Thomas, US Virgin Islands	0.1.952	1	VII	1	0–60	Suspected Cheloniidae	unidenfitied long bone	1	–	T15	*Chelonia mydas* (1)
91	Magens Bay	St. Thomas, US Virgin Islands	0.1.952	1	VII	1	0–60	Suspected Cheloniidae	suspected carapace/plastron fragment	1	–	T16	*Chelonia mydas* (1)
92	Magens Bay	St. Thomas, US Virgin Islands	0.1.952	1	VII	2	60–90	Suspected Cheloniidae	suspected carapace/plastron fragment	1	–	T17	*Chelonia mydas* (1)
93	Magens Bay	St. Thomas, US Virgin Islands	0.1.952	1	VII	2	60–90	Suspected Cheloniidae	unidentified	1	–	T18	*Eretmochelys imbricata*
94	Magens Bay	St. Thomas, US Virgin Islands	0.1.952	1	VII	2	60–90	Suspected Cheloniidae	suspected carapace/plastron fragment	1	–	T19	*Chelonia mydas* (1)
95	Magens Bay	St. Thomas, US Virgin Islands	0.1.952	1	VII	2	60–90	Suspected Cheloniidae	suspected carapace/plastron fragment	1	–	T21	*Chelonia mydas* (1)
96	Magens Bay	St. Thomas, US Virgin Islands	0.1.952	1	VII	3	90–125	Suspected Cheloniidae	suspected carapace/plastron fragment	1	–	T24	*Eretmochelys imbricata*
97	Magens Bay	St. Thomas, US Virgin Islands	0.1.952	1	VII	3	90–125	Suspected Cheloniidae	suspected carapace/plastron fragment	1	–	T26	Emydidae
98	Magens Bay	St. Thomas, US Virgin Islands	0.1.952	1	VII	3	90–125	Suspected Cheloniidae	suspected carapace/plastron fragment	1	–	T27	*Eretmochelys imbricata*
99	Magens Bay	St. Thomas, US Virgin Islands	0.1.952	1	VII	3	90–125	Suspected Cheloniidae	suspected carapace/plastron fragment	1	–	T28	*Chelonia mydas* (1)
100	Magens Bay	St. Thomas, US Virgin Islands	0.1.952	1	VII	3	90–125	Suspected Cheloniidae	unidentified	1	–	T29	*Eretmochelys imbricata*
101	Magens Bay	St. Thomas, US Virgin Islands	0.1.952	1	VII	3	90–125	Suspected Cheloniidae	suspected carapace/plastron fragment	1	–	T30	*Chelonia mydas* (1)
102	Magens Bay	St. Thomas, US Virgin Islands	0.1.952	1	VII	3	90–125	Suspected Cheloniidae	suspected carapace/plastron fragment	1	–	T32	*Eretmochelys imbricata*
103	Magens Bay	St. Thomas, US Virgin Islands	0.1.952	1	VII	3	90–125	Suspected Cheloniidae	unidenfitied long bone	1	–	T33	*Eretmochelys imbricata*
104	Magens Bay	St. Thomas, US Virgin Islands	0.1.953	1	VIII	–	80–110	Suspected Cheloniidae	suspected carapace/plastron fragment	1	–	T39	*Chelonia mydas* (1)
105	Magens Bay	St. Thomas, US Virgin Islands	0.1.953	1	VIII	–	80–110	Suspected Cheloniidae	suspected carapace/plastron fragment	1	–	T40	Emydidae
106	Magens Bay	St. Thomas, US Virgin Islands	0.1.953	1	VIII	–	–	Suspected Cheloniidae	unidentified	1	–	T41	*Chelonia mydas* (1)
107	Magens Bay	St. Thomas, US Virgin Islands	0.1.953	1	VIII	–	–	Suspected Cheloniidae	unidentified	1	–	T42	*Chelonia mydas* (1)
108	Magens Bay	St. Thomas, US Virgin Islands	0.1.953	1	VIII	–	–	Suspected Cheloniidae	unidentified	1	–	T44	*Chelonia mydas* (1)
109	Magens Bay	St. Thomas, US Virgin Islands	0.1.953	1	VIII	–	–	Suspected Cheloniidae	unidentified	1	–	T45	*Chelonia mydas* (1)
110	Coral Bay	St. John, US Virgin Islands	0.5.10	A	Test pit 1	–	–	Suspected Cheloniidae	unidentified	1	–	T48	*Chelonia mydas* (1)
111	Coral Bay	St. John, US Virgin Islands	0.5.14	A	Test pit 2	–	–	Suspected Cheloniidae	suspected carapace/plastron fragment	1	–	T49	*Chelonia mydas* (1)
112	Coral Bay	St. John, US Virgin Islands	0.5.14	A	Test pit 2	–	–	Suspected Cheloniidae	unidentified	1	–	T50	*Eretmochelys imbricata*
113	Coral Bay	St. John, US Virgin Islands	0.5.14	A	Test pit 2	–	–	Suspected Cheloniidae	suspected carapace/plastron fragment	1	–	T51	*Chelonia mydas* (1)
114	Coral Bay	St. John, US Virgin Islands	0.5.147	A	–	–	–	Suspected Cheloniidae	unidenfitied long bone	1	–	T52	POOR

115	Coral Bay	St. John, US Virgin Islands	0.5.147	A	–	–	–	Suspected Cheloniidae	suspected carapace/plastron fragment	1	–	T53	*Chelonia mydas* (1)
116	Coral Bay	St. John, US Virgin Islands	0.5.147	A	–	–	–	Suspected Cheloniidae	suspected carapace/plastron fragment	1	–	T54	POOR
117	Coral Bay	St. John, US Virgin Islands	0.5.147	A	–	–	–	Suspected Cheloniidae	suspected carapace/plastron fragment	1	–	T55	*Chelonia mydas* (1)
118	Coral Bay	St. John, US Virgin Islands	0.5.147	A	–	–	–	Suspected Cheloniidae	suspected carapace/plastron fragment	1	–	T56	*Chelonia mydas* (1)
119	Coral Bay	St. John, US Virgin Islands	0.5.23	A	IV	1	0–(25?)30	Suspected Cheloniidae	suspected carapace/plastron fragment	1	–	T57	*Chelonia mydas* (1)
120	Coral Bay	St. John, US Virgin Islands	0.5.23	A	IV	1	0–(25?)30	Suspected Cheloniidae	unidenfitied long bone	1	–	T58	POOR
121	Coral Bay	St. John, US Virgin Islands	0.5.23	A	IV	1	0–(25?)30	Suspected Cheloniidae	unidentified	1	–	T59	POOR
122	Coral Bay	St. John, US Virgin Islands	0.5.23	A	IV	1	0–(25?)30	Suspected Cheloniidae	unidenfitied long bone	1	–	T60	*Eretmochelys imbricata*
123	Little Cruz Bay	St. John, US Virgin Islands	0.8.60	A	V	2	–	Suspected Cheloniidae	suspected carapace/plastron fragment	1	–	T61	*Chelonia mydas* (1)
124	Little Cruz Bay	St. John, US Virgin Islands	0.8.60	A	V	2	–	Suspected Cheloniidae	suspected carapace/plastron fragment	1	–	T62	*Chelonia mydas* (1)
125	Little Cruz Bay	St. John, US Virgin Islands	0.8.54	A	I	2	–	Suspected Cheloniidae	suspected carapace/plastron fragment	1	–	T63	*Chelonia mydas* (1)
126	Magens Bay	St. Thomas, US Virgin Islands	0.1.145	1	XII	3	–	Suspected Cheloniidae	suspected carapace/plastron fragment	1	–	T64	*Eretmochelys imbricata*
127	Magens Bay	St. Thomas, US Virgin Islands	0.1.172	1	XI	4	–	Suspected Cheloniidae	suspected carapace/plastron fragment	1	–	T65	*Chelonia mydas* (1)
128	Magens Bay	St. Thomas, US Virgin Islands	0.1.814	1	VIII	4	–	Suspected Cheloniidae	suspected carapace/plastron fragment	1	–	T66	*Chelonia mydas* (1)
129	Casey Long Bay	St. John, US Virgin Islands	0.7.50	A	II	1	–	Suspected Cheloniidae	suspected carapace/plastron fragment	1	–	T67	*Eretmochelys imbricata*
130	Casey Long Bay	St. John, US Virgin Islands	0.7.53	A	III	2	–	Suspected Cheloniidae	suspected carapace/plastron fragment	1	–	T68	*Eretmochelys imbricata*

Supplementary S1: Full ancient DNA methodology

1. *Authentication Criteria*

Ancient DNA (aDNA) analyses on archaeological marine turtle bone were carried out using two independent, physically isolated laboratories equipped with an ultra-filtered air supply, maintaining positive displacement pressure, at The University of Manchester (Manchester Institute of Biotechnology). Sample pre-treatment and DNA extractions were carried out in a Class II biological safety cabinet in one laboratory, and polymerase chain reactions (PCRs) were prepared in a laminar-flow cabinet in the second laboratory. All post-PCR works were carried out in a third multiple-user laboratory.

Access to the forensic rooms was managed and the rooms were continuously UV irradiated when not in use. All surfaces within the laboratories were cleaned with 5% bleach and 70% ethanol, and all utensils and equipment were treated with DNA-Away (Molecular BioProducts) before and after use. Plastic consumables were UV irradiated before use in a Crosslinker (254 nm, 120,000 mJ cm^{-2}) for 2 × 5 min, with 180° rotation between two exposures. All reagents and consumables were DNase- and RNase-free and aqueous solutions were irradiated for 10 min before every use.

Personnel wore protective clothing, including forensic suits, face masks, hair nets, goggles and two pairs of sterile gloves at all times. DNA extractions were accompanied by two extraction blanks (extraction following protocol but without skeletal material) and PCRs were accompanied by two PCR blanks (set up with water rather than DNA extract). MtDNA sequences were obtained from all personnel involved in DNA extraction.

2. *Sample Pre-treatment*

Six archaeological marine turtle bone samples were selected for aDNA analysis (Table S1.1). Two different extraction protocols were attempted:

- Dabney *et al.,* (1) on samples GP9 and GP10 from Garden Patch (both from Area X, TU 1, Level G, 60–70 cm depth), and GB64 and GB72 from Grand Bay (both from Trench 446, Square 12, Level 4);
- Rohland and Hofreiter, (2) with modifications from Rohland and Hofreiter, (3) on samples GP9, GB72 (as above), plus samples GP20 and GP56 from Garden Patch (Mound II, TU 2).

Samples GP10 and GB64 were identified through collagen fingerprinting as *Chelonia mydas*, whereas samples GP9, GP20, GP56 and GB72 were identified as *Chelonia* sp., potentially from a different genetic stock.

Table S1.1: List of samples subjected to aDNA analysis.

ZooMS Code	Site	Origin	FS Lot #:	Provenience	Level	Depth (cm)	Morphological ID	Element	ZooMS ID	Extraction Protocol(s)
GP9	Garden Patch	Florida, USA	19	Area X, TU 1	G	60–70	Cheloniidae, cf. *Caretta caretta*	post cranial fragment	*Chelonia* sp.	(1, 3)
GP10	Garden Patch	Florida, USA	19	Area X, TU 1	G	60–70	Cheloniidae, cf. *Caretta caretta*	cf. forelimb	*Chelonia mydas*	(1)
GP20	Garden Patch	Florida, USA	29	Mound II, TU 2	C	20–30	Cheloniidae	marginal fragment	*Chelonia* sp.	(3)
GP56	Garden Patch	Florida, USA	172	Mound II, TU 2	P	150–160	Cheloniidae	marginal fragment	*Chelonia* sp.	(3)
GB64	Grand Bay	Carriacou, Grenadines	05CGB000 612BOA	Trench 446, Square 12	4	–	Cheloniidae	sample from humerus, left, distal 1/3	*Chelonia mydas*	(1)
GB72	Grand Bay	Carriacou, Grenadines	05CGB000 612BOA	Trench 446, Square 12	4	–	Cheloniidae	sample from humerus, right, proximal 1/2	*Chelonia sp.*	(1, 3)

Samples were decontaminated by removal of the outer 1–2 mm using two sterile and irradiated scalpel blades. Each sample was then placed in a clear DNA-free bag and was UV irradiated using a Crosslinker as above. After irradiation each sample was placed in a second clear DNA-free bag, wrapped in two pieces of sterile aluminium foil and crushed into a powder. Bone powder was weighed and transferred in 1.5 ml Eppendorf tubes and stored in -20°C until extraction. DNA extractions were prepared from 250–500 mg of bone powder.

PCRs were directed at five mitochondrial loci, including sections of the hypervariable regions (HVR) and the 12S and 16S genes using previously established, as well as newly designed primers, based upon the complete mitochondrial DNA (mtDNA) sequence for *Chelonia mydas* (NCBI Accession AB012104.1) (Table S1.2). Reactions were setup according to Lee *et al.,* (4) in a 42.2 uL final volume, including 18 uL of DNase/RNase –free distilled water, 4 uL of 10 X buffer, 4 uL of 20 mM dNTP mix, 4 uL of $MgCl_2$, 4 uL of each of the forward and reverse primers, 0.2 uL of *Taq* DNA polymerase (NEB) and 4 uL of DNA template. Thermocycling conditions for all PCRs were: 95°C for 5 min to activate the *Taq* polymerase followed by 42–44 cycles at 95°C for 30s, a variable annealing temperature for 45s (see Table S1.2), 72°C for 1 min and a final extension of 6 min. PCRs were resolved in a 2% agarose gel using electrophoresis at 100 Volts stained with 0.8 uL GelRed.

Samples were set for sequencing to Eurofins Genomics and sequences were analysed on Geneious Prime. Marine turtle reference genomes were downloaded from NCBI (www.ncbi.nlm.nih.gov) and default algorithms for alignment were used.

Table S1.2: List of primers used in this study, designed on the basis of the complete mtDNA sequence for *Chelonia mydas.*

**Primer name**	**Sequence, 5'–3'**	**Genomic position**	**Fragment size**	**Annealing temp.**	**Source**
L1091 (12S)<br>H1478 (12S)	F-AAAAAGCTTCAAACTGGGATTAGATACCCCACTAT<br>R-TGACTGCAGAGGGTGACGGGCGGTGTGT	491–947	456	45 °C	(5)
AR (16S)<br>BR (16S)	F- CGCCTGTTTATCAAAAACAT<br>R-CCGGTCTGAACTCAGATCACGT	1959–2561	602	45 °C	(6)
XmyHVR2	F-CGGTATCAGGCACACCAT<br>R-TCGTGCCAGCCACCG	179–196<br>311–325	115	55 °C	Designed for this study
Xmy16Sb	F- CAAAGCGAACTAAAGTCTGAA<br>R- CTGGTTGCTCAATAAAAGAAT	1343–1363<br>1485–1505	122	57 °C	Designed for this study
Chmy1	F- TGATCTATTCTGGCCTCTGGT<br>R- GCCCACATAACTGATACCTGC	15,901–15,921<br>16,070–16,090	148	56 °C	Designed for this study

References

1. Dabney J, Knapp M, Glocke I, Gansauge M-T, Weihmann A, Nickel B, et al. Complete mitochondrial genome sequence of a Middle Pleistocene cave bear reconstructed from ultrashort DNA fragments. Proceedings of the National Academy of Sciences. 2013;110(39):15758–63.
2. Rohland N, Hofreiter M. Comparison and optimization of ancient DNA extraction. Biotechniques. 2007;42(3):343–52.
3. Rohland N, Hofreiter M. Ancient DNA extraction from bones and teeth. Nature Protocols. 2007;2(7):1756.
4. Le M, Raxworthy CJ, McCord WP, Mertz L. A molecular phylogeny of tortoises (Testudines: Testudinidae) based on mitochondrial and nuclear genes. Molecular Phylogenetics and Evolution. 2006;40(2):517–31.
5. Kocher TD, Thomas WK, Meyer A, Edwards SV, Pääbo S, Villablanca FX, et al. Dynamics of mitochondrial DNA evolution in animals: amplification and sequencing with conserved primers. Proceedings of the National Academy of Sciences. 1989;86(16):6196–200.
6. Palumbi S, Martin A, Romano S, McMillan W, Stice L, Grabowski G. The simple fool's guide to PCR, version 2.0. University of Hawaii, Honolulu. 1991;45.

Supplementary Table S2: Details of the modern Testudines samples used for this study.

Sample Code	Class	Order	Sub-Order	Family	Species	Common Name	Origin	Bone Element
FLMNH 57247	Reptilia	Testudines	Cryptodira	Cheloniidae	*Chelonia mydas*	Green sea turtle	Vietnam, Asia (Pacific)	Supra occipital
UF42972	Reptilia	Testudines	Cryptodira	Cheloniidae	*Chelonia mydas*	Green sea turtle	Monroe, Florida, USA (Atlantic)	Pleural scute
UF52003	Reptilia	Testudines	Cryptodira	Cheloniidae	*Chelonia mydas*	Green sea turtle	Sonora, Mexico (Pacific)	Pleural scute
WAM R177325	Reptilia	Testudines	Cryptodira	Cheloniidae	*Natator depressus*	Flatback sea turtle	Australia	Scute
FLMNH M.54206	Reptilia	Testudines	Cryptodira	Cheloniidae	*Caretta caretta*	Loggerhead sea turtle	Brevard, Florida, USA	Metapodial
FLMNH 62368	Reptilia	Testudines	Cryptodira	Cheloniidae	*Eretmochelys imbricata*	Hawksbill sea turtle	Puerto Rico, Caribbean	Hyloid
FLMNH 22371	Reptilia	Testudines	Cryptodira	Cheloniidae	*Lepidochelys kempii*	Kemp's ridley sea turtle	Pinellas, Florida, USA	Phalanx 2
FLMNH 44952	Reptilia	Testudines	Cryptodira	Cheloniidae	*Lepidochelys olivacea*	Olive ridley sea turtle	Guanacaste, Costa Rica	Hatchling (full)
FLMNH 37557	Reptilia	Testudines	Cryptodira	Dermochelyidae	*Dermochelys coriacea*	Leatherback sea turtle	Nassau, Florida, USA	Phalanx 1
UF12695	Reptilia	Testudines	Cryptodira	Chelydridae	*Macrochelys suwanniensis*	Suwanee snapping turtle	Lafayette, Florida, USA	Pleural scute
UOM_ST1	Reptilia	Testudines	Cryptodira	Chelydridae	*Chelydra serpentina*	Common snapping turtle	[unknown]	Postcranial bone
UF172810	Reptilia	Testudines	Cryptodira	Kinosternidae	*Kinosternon baurii*	Striped mud turtle	Miami-Dade, Florida, USA	Hypoplastron
UF57434	Reptilia	Testudines	Cryptodira	Kinosternidae	*Kinosternon subrubrum*	Common mud turtle	Lee, Florida, USA	Pleural scute
UF47704	Reptilia	Testudines	Cryptodira	Kinosternidae	*Sternotherus odoratus*	Common musk turtle	Kosciusko, Indiana, USA	Peripheral scute
UF153668	Reptilia	Testudines	Cryptodira	Kinosternidae	*Sternotherus minor*	Loggerhead musk turtle	Alachua, Florida, USA	Pleural scute
UF152640	Reptilia	Testudines	Cryptodira	Emydidae	*Clemmys guttata*	Spotted turtle	Alachua, Florida, USA	Pleural scute
UF154416	Reptilia	Testudines	Cryptodira	Emydidae	*Terrapene carolina carolina*	Eastern box turtle	Etowah, Alabama, USA	Peripheral scute
UF151665	Reptilia	Testudines	Cryptodira	Emydidae	*Terrapene carolina major*	Gulf Coast box turtle	Wakulla, Florida, USA	Pleural scute
UF41524	Reptilia	Testudines	Cryptodira	Emydidae	*Deirochelys reticularia*	Chicken turtle	Wakulla, Florida, USA	Pleural scute
UF154029	Reptilia	Testudines	Cryptodira	Emydidae	*Graptemys barbouri*	Barbour's map turtle	Jackson, Florida, USA	Pleural scute
UF3929	Reptilia	Testudines	Cryptodira	Emydidae	*Graptemys pseudogeographica*	False map turtle	Tennessee, USA	Pleural scute
UF35022	Reptilia	Testudines	Cryptodira	Emydidae	*Graptemys geographica*	Common map turtle	Green, Kentucky, USA	Pleural scute
UF137329	Reptilia	Testudines	Cryptodira	Emydidae	*Malaclemys terrapin*	Diamondback turtle	Pinellas, Florida, USA	Pleural scute
UF151150	Reptilia	Testudines	Cryptodira	Emydidae	*Pseudemys concinna*	River cooter	Florida's Gulf coast, USA	Peripheral scute
UF167048	Reptilia	Testudines	Cryptodira	Emydidae	*Pseudemys floridana*	River cooter turtle	Brevard, Florida, USA	Pleural scute
UF141631	Reptilia	Testudines	Cryptodira	Emydidae	*Pseudemys nelsoni*	Florida redbelly turtle	Dixie, Florida, USA	Pleural scute
UF150314	Reptilia	Testudines	Cryptodira	Emydidae	*Pseudemys floridana peninsularis*	Florida cooter turtle	[unlisted]	Hypoplastron
UF150619	Reptilia	Testudines	Cryptodira	Emydidae	*Pseudemys suwanniensis*	Cooter turtle	Leon, Florida, USA	Pleural scute
UF142391	Reptilia	Testudines	Cryptodira	Emydidae	*Trachemys scripta elegans*	Red-eared slider	Saint Johns, Florida, USA	Hypoplastron
UF151640	Reptilia	Testudines	Cryptodira	Testudinidae	*Gopherus polyphemus*	Gopher tortoise	Hamilton, Florida, USA	Pleural scute
US182296	Reptilia	Testudines	Cryptodira	Trionychidae	*Apalone ferox*	Florida soft-shell turtle	Alachua, Florida, USA	Peripheral scute
UF76532	Reptilia	Testudines	Cryptodira	Trionychidae	*Apalone spinifera*	Spiny soft-shell turtle	[unlisted]	Pleural scute

Supplementary Table S3: Estimated amino acid sequences and *m/z* values of collagen (I) biomarkers for all extant marine turtle species.

KEY

Green = Modern
Blue = Ancient
X = Amino acid unknown
* = Publically available collagen (I) sequence acquired from BLAST/P®
. = Amino acid is unchanged from the top sequence

Table S3.1: Datasets aquired for each sea turtle species analysed.

Taxonomy	Species	PMF	MS/MS	BLAST/P®
Reptilia: Testudines, Cheloniidae	*Chelonia mydas*	✓	✓	✓
Reptilia: Testudines, Cheloniidae	*Natator depressus*	✓	✓	
Reptilia: Testudines, Cheloniidae	*Caretta caretta*	✓	✓	
Reptilia: Testudines, Cheloniidae	*Eretmochelys imbricata*	✓	✓	
Reptilia: Testudines, Cheloniidae	*Lepidochelys kempii*	✓	✓	
Reptilia: Testudines, Cheloniidae	*Lepidochelys olivacea*	✓	✓	
Reptilia: Testudines, Dermochelyidae	*Dermochelys coriacea*	✓	✓	
Reptilia: Testudines, Cheloniidae	*Chelonia* spp. (GP9)	✓	✓	

Table S3.2: Estimated MASCOT-derived sequences for peptide COL1A1T47

Taxonomy	Species	M+H$^+$ *m/z* value	Peptide COL1A1T47
Reptilia: Testudines, Cheloniidae	*Chelonia mydas**	1136.5	G V Q G A P G P Q G P R
Reptilia: Testudines, Cheloniidae	*Natator depressus*	1136.5	. . . .
Reptilia: Testudines, Cheloniidae	*Caretta caretta*	1136.5	. . . .
Reptilia: Testudines, Cheloniidae	*Eretmochelys imbricata*	1136.5	. . . .
Reptilia: Testudines, Cheloniidae	*Lepidochelys kempii*	1152.6	. . . . L . . . .
Reptilia: Testudines, Cheloniidae	*Lepidochelys olivacea*	1152.6	. . . . L . . . .
Reptilia: Testudines, Dermochelyidae	*Dermochelys coriacea*	1136.5	. . . .
Reptilia: Testudines, Cheloniidae	*Chelonia* spp. (GP9)	1136.5	. . . .

Table S3.3: Estimated MASCOT-derived sequences for peptide COL1A2T85

Taxonomy	Species	M+H$^+$ *m/z* value	Peptide COL1A2T85 (A)
Reptilia: Testudines, Cheloniidae	*Chelonia mydas**	1220.7	N G L P G P I G P A G V R
Reptilia: Testudines, Cheloniidae	*Natator depressus*	1220.7	. . . .
Reptilia: Testudines, Cheloniidae	*Caretta caretta*	1220.7	. . . .
Reptilia: Testudines, Cheloniidae	*Eretmochelys imbricata*	1220.7	. . . .
Reptilia: Testudines, Cheloniidae	*Lepidochelys kempii*	1220.7	. . . .
Reptilia: Testudines, Cheloniidae	*Lepidochelys olivacea*	1220.7	. . . .
Reptilia: Testudines, Dermochelyidae	*Dermochelys coriacea*	1220.7	. . . .
Reptilia: Testudines, Cheloniidae	*Chelonia* spp. (GP9)	1220.7	. . . .

Table S3.4: Estimated MASCOT-derived sequences for peptide COL1A1T28

Taxonomy	Species	M+H$^+$ *m/z* value	Peptide COL1A1T28
Reptilia: Testudines, Cheloniidae	*Chelonia mydas**	1393.7	G E P G P A G L P G P A G E R
Reptilia: Testudines, Cheloniidae	*Natator depressus*	1393.7	. . . .
Reptilia: Testudines, Cheloniidae	*Caretta caretta*	1393.7	. . . .
Reptilia: Testudines, Cheloniidae	*Eretmochelys imbricata*	1393.7	. . . .
Reptilia: Testudines, Cheloniidae	*Lepidochelys kempii*	1393.7	. . . .
Reptilia: Testudines, Cheloniidae	*Lepidochelys olivacea*	1393.7	. . . .
Reptilia: Testudines, Dermochelyidae	*Dermochelys coriacea*	1393.7	. . . .
Reptilia: Testudines, Cheloniidae	*Chelonia* spp. (GP9)	1393.7	. . . .

Table S3.5: Estimated MASCOT-derived sequences for peptide COL1A2T43

Taxonomy	Species	M+H$^+$ *m/z* value	Peptide COL1A2T43 (B)
Reptilia: Testudines, Cheloniidae	*Chelonia mydas**	1453.7	G I P G E F G L P G P A G P R
Reptilia: Testudines, Cheloniidae	*Natator depressus*	1453.7	. . . .
Reptilia: Testudines, Cheloniidae	*Caretta caretta*	1453.7	. . . .
Reptilia: Testudines, Cheloniidae	*Eretmochelys imbricata*	1443.7	. . . . S . . . .
Reptilia: Testudines, Cheloniidae	*Lepidochelys kempii*	1453.7	. . . .
Reptilia: Testudines, Cheloniidae	*Lepidochelys olivacea*	1453.7	. . . .
Reptilia: Testudines, Dermochelyidae	*Dermochelys coriacea*	1453.7	. . . .
Reptilia: Testudines, Cheloniidae	*Chelonia* spp. (GP9)	1453.7	. . . .

Table S3.6: Estimated MASCOT-derived sequences for peptide COL1A1T62

Taxonomy	Species	M+H$^+$ *m/z* value	Peptide COL1A1T62
Reptilia: Testudines, Cheloniidae	*Chelonia mydas**	1459.7	G S A G P P G A T G F P G A A G R
Reptilia: Testudines, Cheloniidae	*Natator depressus*	1459.7	. . . .
Reptilia: Testudines, Cheloniidae	*Caretta caretta*	1459.7	. . . .
Reptilia: Testudines, Cheloniidae	*Eretmochelys imbricata*	1459.7	. . . .
Reptilia: Testudines, Cheloniidae	*Lepidochelys kempii*	1459.7	. . . .
Reptilia: Testudines, Cheloniidae	*Lepidochelys olivacea*	1459.7	. . . .
Reptilia: Testudines, Dermochelyidae	*Dermochelys coriacea*	1459.7	. . . .
Reptilia: Testudines, Cheloniidae	*Chelonia* spp. (GP9)	1459.7	. . . .

Table S3.7: Estimated MASCOT-derived sequences for peptide COL1A1T79

Taxonomy	Species	M+H$^+$ *m/z* value	Peptide COL1A1T79
Reptilia: Testudines, Cheloniidae	*Chelonia mydas**	1516.7	G E T G P A G P A G P A G P A G P R
Reptilia: Testudines, Cheloniidae	*Natator depressus*	1490.7	. . . . A .
Reptilia: Testudines, Cheloniidae	*Caretta caretta*	1490.7	. . . . A .
Reptilia: Testudines, Cheloniidae	*Eretmochelys imbricata*	1490.7	. . . . A .
Reptilia: Testudines, Cheloniidae	*Lepidochelys kempii*	1490.7	. . . . A .
Reptilia: Testudines, Cheloniidae	*Lepidochelys olivacea*	1490.7	. . . . A .
Reptilia: Testudines, Dermochelyidae	*Dermochelys coriacea*	1490.7	. . . . A .
Reptilia: Testudines, Cheloniidae	*Chelonia* spp. (GP9)	1516.7	. . . .

Table S3.8: Estimated MASCOT-derived sequences for peptide COL1A1T21

Taxonomy	Species	M+H$^+$ *m/z* value	Peptide COL1A1T21
Reptilia: Testudines, Cheloniidae	*Chelonia mydas**	1572.7	G A T G A P G I A G A P G F P G A R
Reptilia: Testudines, Cheloniidae	*Natator depressus*	1572.7	. . . .
Reptilia: Testudines, Cheloniidae	*Caretta caretta*	1572.7	. . . .
Reptilia: Testudines, Cheloniidae	*Eretmochelys imbricata*	1572.7	. . . .
Reptilia: Testudines, Cheloniidae	*Lepidochelys kempii*	1572.7	. . . .
Reptilia: Testudines, Cheloniidae	*Lepidochelys olivacea*	1572.7	. . . .
Reptilia: Testudines, Dermochelyidae	*Dermochelys coriacea*	1572.7	. . . .
Reptilia: Testudines, Cheloniidae	*Chelonia* spp. (GP9)	1572.7	. . . .

Table S3.9: Estimated MASCOT-derived sequences for peptide COL1A2T69

Taxonomy	Species	M+H$^+$ *m/z* value	Peptide COL1A2T69 (D)
Reptilia: Testudines, Cheloniidae	*Chelonia mydas**	2097.0	G L P G I S G G T G E P G P A G I S G P S G S R
Reptilia: Testudines, Cheloniidae	*Natator depressus*	2107.0	. . . . P .
Reptilia: Testudines, Cheloniidae	*Caretta caretta*	2097.0	. . . .
Reptilia: Testudines, Cheloniidae	*Eretmochelys imbricata*	2097.0	. . . .
Reptilia: Testudines, Cheloniidae	*Lepidochelys kempii*	2097.0	. . . .
Reptilia: Testudines, Cheloniidae	*Lepidochelys olivacea*	2097.0	. . . .
Reptilia: Testudines, Dermochelyidae	*Dermochelys coriacea*	2125.0	. . . . V . . . .
Reptilia: Testudines, Cheloniidae	*Chelonia* spp. (GP9)	2097.0	. . . .

Table S3.10: Estimated MASCOT-derived sequences for peptide COL1A2T65/66

Taxonomy	Species	M+H$^+$ *m/z* value	Peptide COL1A2T65/66
Reptilia: Testudines, Cheloniidae	*Chelonia mydas**	2353.1	G D T G P V G R P G E Q G I V G P P G F I G E K
Reptilia: Testudines, Cheloniidae	*Natator depressus*	2341.1	. . . . T . . .
Reptilia: Testudines, Cheloniidae	*Caretta caretta*	2341.1	. . . . T . . .
Reptilia: Testudines, Cheloniidae	*Eretmochelys imbricata*	2341.1	. . . . T . . .
Reptilia: Testudines, Cheloniidae	*Lepidochelys kempii*	2341.1	. . . . T . . .
Reptilia: Testudines, Cheloniidae	*Lepidochelys olivacea*	2341.1	. . . . T . . .
Reptilia: Testudines, Dermochelyidae	*Dermochelys coriacea*	2311.1	. . . . A . . . . . .
Reptilia: Testudines, Cheloniidae	*Chelonia* spp. (GP9)	2353.1	. . . .

Table S3.11: Estimated MASCOT-derived sequences for peptide COL1A2T60

Taxonomy	Species	M+H$^+$ *m/z* value	Peptide COL1A2T60
Reptilia: Testudines, Cheloniidae	*Chelonia mydas**	2455.3	G E V G P L G A I G P A G S S G P A G P I G A A G P A G G R
Reptilia: Testudines, Cheloniidae	*Natator depressus*	2455.3	. . . .
Reptilia: Testudines, Cheloniidae	*Caretta caretta*	2455.3	. . . .
Reptilia: Testudines, Cheloniidae	*Eretmochelys imbricata*	2485.3	. . . . T . . .
Reptilia: Testudines, Cheloniidae	*Lepidochelys kempii*	2455.3	. . . .
Reptilia: Testudines, Cheloniidae	*Lepidochelys olivacea*	2455.3	. . . .
Reptilia: Testudines, Dermochelyidae	*Dermochelys coriacea*	2510.3	. . L . . . . N . . . . A .
Reptilia: Testudines, Cheloniidae	*Chelonia* spp. (GP9)	2455.3	. . . .

Table S3.12: Estimated MASCOT-derived sequences for peptide COL1A1T85

Taxonomy	Species	M+H$^+$ *m/z* value	Peptide COL1A1T85
Reptilia: Testudines, Cheloniidae	*Chelonia mydas**	2705.4	G F S G L Q G P P G P P G S P G E Q G P S G A S G P A G P R
Reptilia: Testudines, Cheloniidae	*Natator depressus*	2705.4	. . . .
Reptilia: Testudines, Cheloniidae	*Caretta caretta*	2705.4	. . . .
Reptilia: Testudines, Cheloniidae	*Eretmochelys imbricata*	2705.4	. . . .
Reptilia: Testudines, Cheloniidae	*Lepidochelys kempii*	2705.4	. . . .
Reptilia: Testudines, Cheloniidae	*Lepidochelys olivacea*	2705.4	. . . .
Reptilia: Testudines, Dermochelyidae	*Dermochelys coriacea*	2705.4	. . . .
Reptilia: Testudines, Cheloniidae	*Chelonia* spp. (GP9)	2705.4	. . . .

Table S3.13: Estimated MASCOT-derived sequences for peptide COL1A2T41/42

Taxonomy	Species	M+H$^+$ *m/z* value	Peptide COL1A2T41/42
Reptilia: Testudines, Cheloniidae	*Chelonia mydas**	2790.3	G E Q G P A G P P G F Q G L P G P S G P A G E A G K P G D R
Reptilia: Testudines, Cheloniidae	*Natator depressus*	2790.3	. . . .
Reptilia: Testudines, Cheloniidae	*Caretta caretta*	2790.3	. . . .
Reptilia: Testudines, Cheloniidae	*Eretmochelys imbricata*	2790.3	. . . .
Reptilia: Testudines, Cheloniidae	*Lepidochelys kempii*	2790.3	. . . .
Reptilia: Testudines, Cheloniidae	*Lepidochelys olivacea*	2790.3	. . . .
Reptilia: Testudines, Dermochelyidae	*Dermochelys coriacea*	2790.3	. . . .
Reptilia: Testudines, Cheloniidae	*Chelonia* spp. (GP9)	2790.3	. . . .

Table S3.14: Estimated MASCOT-derived sequences for peptide COL1A1T55/56

Taxonomy	Species	M+H$^+$ *m/z* value	Peptide COL1A1T55/56 (F)
Reptilia: Testudines, Cheloniidae	*Chelonia mydas**	2843/59.3	G L T G P I G P P G P A G A P G D K G E A G P S G P S G S T G A R
Reptilia: Testudines, Cheloniidae	*Natator depressus*	2869/85.3	. . . . P . . . .
Reptilia: Testudines, Cheloniidae	*Caretta caretta*	2869/85.3	. . . . P . . . .
Reptilia: Testudines, Cheloniidae	*Eretmochelys imbricata*	2843/59.3	. . . . S . . . . P . . . .
Reptilia: Testudines, Cheloniidae	*Lepidochelys kempii*	2843/59.3	. . . . A . . P . . . .
Reptilia: Testudines, Cheloniidae	*Lepidochelys olivacea*	2843/59.3	. . . . A . . P . . . .
Reptilia: Testudines, Dermochelyidae	*Dermochelys coriacea*	2853/69.3	. . . . A P . P . . . .
Reptilia: Testudines, Cheloniidae	*Chelonia* spp. (GP9)	2843/59.3	. . . .

Table S3.15: Estimated MASCOT-derived sequences for peptide COL1A2T67

Taxonomy	Species	M+H$^+$ *m/z* value	Peptide COL1A2T67 (G)
Reptilia: Testudines, Cheloniidae	*Chelonia mydas**	2929.5	G P S G E P G G A G P P G T S G P Q G I L G A P G I L G L P G S R
Reptilia: Testudines, Cheloniidae	*Natator depressus*	2929.5	. . . .
Reptilia: Testudines, Cheloniidae	*Caretta caretta*	2929.5	. . . .
Reptilia: Testudines, Cheloniidae	*Eretmochelys imbricata*	2929.5	. . . .
Reptilia: Testudines, Cheloniidae	*Lepidochelys kempii*	2929.5	. . . .
Reptilia: Testudines, Cheloniidae	*Lepidochelys olivacea*	2929.5	. . . .
Reptilia: Testudines, Dermochelyidae	*Dermochelys coriacea*	2899.5	. . . . V . . . . A . . . .
Reptilia: Testudines, Cheloniidae	*Chelonia* spp. (GP9)	2929.5	. . . .

Table S3.16: Estimated MASCOT-derived sequences for peptide COL1A2T3

Taxonomy	Species	M+H$^+$ *m/z* value	Peptide COL1A2T3
Reptilia: Testudines, Cheloniidae	*Chelonia mydas**	3007.4	G A P G T S G P P G A Q G F Q G P A G E P G E P G Q T G P V G A R
Reptilia: Testudines, Cheloniidae	*Natator depressus*	3007.4	. . . .
Reptilia: Testudines, Cheloniidae	*Caretta caretta*	3007.4	. . . .
Reptilia: Testudines, Cheloniidae	*Eretmochelys imbricata*	3007.4	. . . .
Reptilia: Testudines, Cheloniidae	*Lepidochelys kempii*	3007.4	. . . .
Reptilia: Testudines, Cheloniidae	*Lepidochelys olivacea*	3007.4	. . . .
Reptilia: Testudines, Dermochelyidae	*Dermochelys coriacea*	3007.4	. . . .
Reptilia: Testudines, Cheloniidae	*Chelonia* spp. (GP9)	3035.7	. V . . . .

Supplementary Table S4.1: Concatenated COL1A1 and COL1A2 amino acid sequences for all extant marine turtle species.

>*Chelonia_mydas* (BLAST/P)
QLAYGYDEKSVGG-LSMPGPMGPAGPRGLPGPPGSPGPQGFQGPPGEPGEPGASGPMGPR
GPAGPPGKNGDDGEAGKPGRPGERGAPGPQGARGLPGTAGLPGMKGHRGFSGLDGAKGDT
GPAGPKGEPGSPGENGAPGQIGPRGLPGERGRPGPSGSAGARGNDGAPGAAGPTGPAGPA
GPPGFPGAVGAKGETGPQGGRGGEGPQGARGEPGAPGPAGAAGPTGNPGSDGQPGAKGAT
GAPGIAGAPGFPGARGPSGPQGPSGAPGPKGNSGEPGAPGNKGDAGAKGEPGPVGVQGPP
GPSGEEGKRGSRGEPGPAGLPGPAGERGAPGSRGFPGSDGISGPKGPTGERGSPGPVGPK
GSPGESGRPGEPGLPGAKGLTGSPGSPGPDGKTGPTGPAGQDGRPGAAGPPGSRGPAGVM
GFPGPKGAAGEPGKPGERGVPGPVGAVGAPGKDGEAGAQGPPGAAGPSGERGEQGPSGAP
GFQGLPGPAGPAGESGKSGEQGVPGDAGAPGPAGSRGERGFPGERGVQGAPGPQGPRGGN
GAPGNDGAKGDAGAPGSPGNQGPPGLQGMPGERGAAGLPGARGDRGDGGPKGADGAPGKD
GPRGLTGPIGPPGPAGAPGDKGEAGPSGPSGSTGARGAPGDRGEPGPPGPAGFAGPPGAD
GQPGAKGETGDAGAKGDAGAPGPAGPTGAPGPSGAVGAPGPKGARGSAGPPGATGFPGAA
GRVGPPGPSGNIGLPGPPGPSGKEGSKGPRGETGPIGRTGEPGPAGAPGPSGEKGSPGAD
GAPGAPGTPGPQGISGQRGVVGLPGQRGERGFPGLPGPSGEPGKQGPSGSSGERGPPGPV
GPPGLAGPPGEAGREGSPGAEGAPGRDGAAGPKGDRGETGPAGPPGAPGAPGAPGPIGPA
GKNGDRGETGPAGPAGPAGPAGPRGAAGPQGPRGDKGETGEHGDRGLKGHRGFSGLQGPP
GPPGSPGEQGPSGASGPAGPRGPPGSSGSPGKDGLNGLPGPIGPPGPRGRTGDVGPAGPP
GPPGPPGPPGPPSGGFDFSFLPQPPQEKAHTDSRYYRQYDPSKAADYGQGPMGIMGPRGA
PGTSGPPGAQGFQGPAGEPGEPGQTGPVGARGPAGPPGKSGEDGHPGKPGRSGERGVPGT
QGARGFPGTPGLPGFKGIRGHNGLDGQRGQAGAPGVKGEPGAHGENGSPGQAGARGLPGE
RGRVGSPGPVGSRGSDGSAGPTGPAGPIGAAGPPGFPGAPGAKGDIGAAGAVGPSGPAGP
RGEPGLPGASGPVGPAGNPGANGIAGAKGAAGLPGVAGAPGLPGPRGIPGPAGPAGTAGA
RGLAGEPGPAGSKGESGNKGEPGAAGSPGPAGPNGEEGKRGSTGEAGATGPPGSAGLRGV
PGSRGLPGSEGRAGSMGLAGSRGSPGPAGARGPGGDSGRPGEPGLLGPRGLPGQPGNPGP
AGKEGPVGFPGADGRVGPIGPAGPRGEPGNIGFPGPKGPNGEPGKPGDKGNVGVAGSRGA
PGPDGNNGVQGPPGVAGNPGAKGEQGPAGPPGFQGLPGPSGPAGEAGKPGDRGIPGEFGL
PGPAGPRGERGAPGESGAVGPVGSMGSRGPTGPPGSDGNKGEPGNVGAPGGPGPAGPGGI
PGERGIAGVPGGKGEKGLPGLRGEAGATGRDGSRGIPGAVGSPGPAGGPGDRGEAGPAGS
AGPAGPRGAPGERGESGPAGPNGFAGPPGAAGQTGAKGERGQKGPKGEVGPLGAIGPAGS
SGPAGPIGAAGPAGGRGDAGPSGATGFPGAAGRTGPPGPAGITGPPGPPGQSGKDGPRGP
RGDTGPVGRPGEQGIVGPPGFIGEKGPSGEPGGAGPPGTSGPQGILGAPGILGLPGSRGE
RGLPGISGGTGEPGPAGISGPSGSRGPSGPSGAPGLNGPPGEAGRDGNPGNDGPPGRDGA
PGFKGERGYPGNNGLAGAAGAPGPHGAVGPSGKPGNRGEPGPVGVTGSIGPAGARGLGGP
QGPRGEKGVPGDKGPRGMPGFKGHNGLQGLPGLAGHHGDQGAPGSTGPAGPRGPAGPSGP
HGKDGRNGLPGPIGPAGVRGSQGSQGPSGPPGPPGHPGPAGPNGGGY--EIGYDAEYYR

>*Chelonia_mydas*_(Pacific)
QLAYGYDEKSVGG-LSMPGPMGPAGPRGLPGPPGSPGPQGFQGPPGEPGEPGASGPMGPR
XXXXXXXXNGDDGEAGKPGRPGERGAPGPQGARGLPGTAGLPGMKGHRGFSGLDGAKGDT
GPAGPKGEPGSPGENGAPGQIGPRGLPGERGRPGPSGSAGARGNDGAPGAAGPTGPAGPA
GPPGFPGAVGAKGETGPQGGRGGEGPQGARGEPGAPGPAGAAGPTGNPGSDGQPGAKGAT
GAPGIAGAPGFPGARGPSGPQGPSGAPGPKGNSGEPGAPGNKGDAGAKGEPGPVGVQGPP
GPSGEEGKRGSRGEPGPAGLPGPAGERGAPGSRGFPGSDGISGPKGPTGERGSPGPVGPK
GSPGESGRPGEPGLPGAKGLTGSPGSPGPDGKTGPTGPAGQDGRPGAAGPPGSRGPAGVM
GFPGPKGAAGEPGKPGERGVPGPVGAVGAPGKDGEAGAQGPPGAAGPSGERGEQGPSGAP
GFQGLPGPAGPAGESGKSGEQGVPGDAGAPGPAGSRXXXGFPGERGVQGAPGPQGPRGGN
GAPGNDGAKGDAGAPGSPGNQGPPGLQGMPGERGAAGLPGARGDRGDGGPKGADGAPGKD
GPRGLTGPIGPPGPAGAPGDKGEAGPSGPSGSTGARGAPGDRGEPGPPGPAGFAGPPGAD
GQPGAKGETGDAGAKGDAGAPGPAGPTGAPGPSGAVGAPGPKGARGSAGPPGATGFPGAA
GRVGPPGPSGNIGLPGPPGPSGKEXXXGPRGETGPIGRTGEPGPAGAPGPSGEKGSPGAD
GAPGAPGTPGPQGISGQRGVVGLPGQRGERGFPGLPGPSGEPGKQGPSGSSGERGPPGPV
GPPGLAGPPGEAGREGSPGAEGAPGRDGAAGPKGDRGETGPAGPPGAPGAPGAPGPIGPA
GKNGDRGETGPAGPAGPAGPAGPRGAAGPQGPRGDKGETGEHGDRXXXGHRGFSGLQGPP
GPPGSPGEQGPSGASGPAGPRGPPGSSGSPGKDGLNGLPGPIGPPGPRGRTGDVGPAGPP
GPPGPPGPPGPPSGGFDFSFLPQPPQEKXXXXXXXXXXXXXXXXAADYGQGPMGIMGPRGA
PGTSGPPGAQGFQGPAGEPGEPGQTGPVGARXXXXXXXXSGEDGHPGKPGRXXXXGVPGT
QGARGFPGTPGLPGFKGIRGHNGLDGQRGQAGAPGVKGEPGAHGENGSPGQAGARXXXXX
XXXVGSPGPVGSRGSDGSAGPTGPAGPIGAAGPPGFPGAPGAKGDIGAAGAVGPSGPAGP
RGEPGLPGASGPVGPAGNPGANGIAGAKGAAGLPGVAGAPGLPGPRGIPGPAGPAGTAGA
RGLAGEPGPAGSKGESGNKGEPGAAGSPGPAGPNGEEGKRGSTGEAGATGPPGSAGLRXX
XXXXGLPGSEGRAGSMGLAGSRGSPGPAGARGPGGDSGRPGEPGLLGPRGLPGQPGNPGP
AGKEGPVGFPGADGRVGPIGPAGPRGEPGNIGFPGPKGPNGEPGKPGDKGNVGVAGSRGA
PGPDGNNGVQGPPGVAGNPGAKGEQGPAGPPGFQGLPGPSGPAGEAGKPGDRGIPGEFGL
PGPAGPRXXXGAPGESGAVGPVGSMGSRGPTGPPGSDGNKGEPGNVGAPGGPGPAGPGGI
PGERGIAGVPGGKGEKGLPGLRXXXXXXXXXXXXGIPGAVGSPGPAGGPGDRGEAGPAGS
AGPAGPRGAPGERGESGPAGPNGFAGPPGAAGQTGAKGERGQKGPKGEVGPLGAIGPAGS
SGPAGPIGAAGPAGGRGDAGPSGATGFPGAAGRTGPPGPAGITGPPGPPGQSGKDGPRGP
RGDTGPVGRPGEQGIVGPPGFIGEKGPSGEPGGAGPPGTSGPQGILGAPGILGLPGSRGE
RGLPGISGGTGEPGPAGISGPSGSRGPSGPSGAPGLNGPPGEAGRDGNPGNDGPPGRDGA
PGFKGERGYPGNNGLAGAAGAPGPHGAVGPSGKPGNRGEPGPVGVTGSIGPAGARGLGGP
QGPRGEKGVPGDKGPRXXXXXXGHNGLQGLPGLAGHHGDQGAPGSTGPAGPRGPAGPSGP
HGKDGRNGLPGPIGPAGVRGSQGSQGPSGPPGPPGHPGPAGPNXXGY--EIGYDAEYYR

>*Chelonia_mydas_*(Atlantic)
QLAYGYDEKSVGG-LSMPGPMGPAGPRGLPGPPGSPGPQGFQGPPGEPGEPGASGPMGPR
GPAGPPGKNGDDGEAGKPGRPGERGAPGPQGARGLPGTAGLPGMKGHRGFSGLDGAKGDT
GPAGPKGEPGSPGENGAPGQIGPRGLPGERGRPGPSGSAGARGNDGAPGAAGPTGPAGPA
GPPGFPGAVGAKGETGPQGGRGGEGPQGARGEPGAPGPAGAAGPTGNPGSDGQPGAKGAT
GAPGIAGAPGFPGARGPSGPQGPSGAPGPKGNSGEPGAPGNKGDAGAKGEPGPVGVQGPP
GPSGEEGKRGSRGEPGPAGLPGPAGERGAPGSRGFPGSDGISGPKGPTGERGSPGPVGPK
GSPGESGRPGEPGLPGAKGLTGSPGSPGPDGKTGPTGPAGQDGRPGAAGPPGSRGPAGVM
GFPGPKGAAGEPGKPGERGVPGPVGAVGAPGKDGEAGAQGPPGAAGPSGERGEQGPSGAP
GFQGLPGPAGPAGESGKSGEQGVPGDAGAPGPAGSRGERGFPGERGVQGAPGPQGPRGGN
GAPGNDGAKGDAGAPGSPGNQGPPGLQGMPGERGAAGLPGARGDRGDGGPKGADGAPGKD
GPRGLTGPIGPPGPAGAPGDKGEAGPSGPSGSTGARGAPGDRGEPGPPGPAGFAGPPGAD
GQPGAKGETGDAGAKGDAGAPGPAGPTGAPGPSGAVGAPGPKGARGSAGPPGATGFPGAA
GRVGPPGPSGNIGLPGPPGPSGKXXXXXPRGETGPIGRTGEPGPAGAPGPSGEKGSPGAD
GAPGAPGTPGPQGISGQRGVVGLPGQRGERGFPGLPGPSGEPGKQGPSGSSGERGPPGPV
GPPGLAGPPGEAGREGSPGAEGAPGRDGAAGPKGDRGETGPAGPPGAPGAPGAPGPIGPA
GKNGDRGETGPAGPAGPAGPAGPRGAAGPQGPRGDKGETGEHGDRXXXGHRGFSGLQGPP
GPPGSPGEQGPSGASGPAGPRGPPGSSGSPGKDGLNGLPGPIGPPGPRXXTGDVGPAGPP
GPPGPPGPPGPPSGGFDFSFLPQPPQEKXXXXXXXXXQYDPSKAADYGQGPMGIMGPRGA
PGTSGPPGAQGFQGPAGEPGEPGQTGPVGARGPAGPPGKSGEDGHPGKPGRXXXRGVPGT
QGARGFPGTPGLPGFKGIRXXXXXXXXXGQAGAPGVKGEPGAHGENGSPGQAGARXXXXX
XXXVGSPGPVGSRGSDGSAGPTGPAGPIGAAGPPGFPGAPGAKGDIGAAGAVGPSGPAGP
RGEPGLPGASGPVGPAGNPGANGIAGAKGAAGLPGVAGAPGLPGPRGIPGPAGPAGTAGA
RGLAGEPGPAGSKGESGNKGEPGAAGSPGPAGPNGEEGKRGSTGEAGATGPPGSAGLRXX
XXXXGLPGSEGRAGSMGLAGSRGSPGPAGARGPGGDSGRPGEPGLLGPRGLPGQPGNPGP
AGKEGPVGFPGADGRVGPIGPAGPRGEPGNIGFPGPKGPNGEPGKPGDKGNVGVAGSRGA
PGPDGNNGVQGPPGVAGNPGAKGEQGPAGPPGFQGLPGPSGPAGEAGKPGDRGIPGEFGL
PGPAGPRGERGAPGESGAVGPVGSMGSRGPTGPPGSDGNKGEPGNVGAPGGPGPAGPGGI
PGERGIAGVPGGKGEKGLPGLRGEAGATGRDGSRGIPGAVGSPGPAGGPGDRGEAGPAGS
AGPAGPRXXXXXXGESGPAGPNGFAGPPGAAGQTGAKGERGQKGPKGEVGPLGAIGPAGS
SGPAGPIGAAGPAGGRGDAGPSGATGFPGAAGRTGPPGPAGITGPPGPPGQSGKDGPRGP
RGDTGPVGRPGEQGIVGPPGFIGEKGPSGEPGGAGPPGTSGPQGILGAPGILGLPGSRGE
RGLPGISGGTGEPGPAGISGPSGSRGPSGPSGAPGLNGPPGEAGRDGNPGNDGPPGRDGA
PGFKGERGYPGNNGLAGAAGAPGPHGAVGPSGKPGNRGEPGPVGVTGSIGPAGARGLGGP
QGPRXXXGVPGDKGPRXXXXXXGHNGLQGLPGLAGHHGDQGAPGSTGPAGPRGPAGPSGP
HGKDGRNGLPGPIGPAGVRXXXXXXXXXXXXXXXXXXXXXXXXXXGY--EIGYDAEYYR

>*Chelonia* sp. (Ancient)
XXXXXXXXXSVGG-LSMPGPMGPAGPRGLPGPPGSPGPQGFQGPPGEPGEPGASGPMGPR
XXXXXXXXNGDDGEAGKPGRPGERGAPGPQGARGLPGTAGLPGMKXXXGFSGLDGAKGDT
GPAGPKGEPGSPGENGAPGQIGPRXXXXXXGRPGPSGSAGARGNDGAPGAAGPTGPAGPA
GPPGFPGAVGAKGETGPQGGRXXXXXXXXXGEPGAPGPAGAAGPTGNPGSDGQPGAKGAT
GAPGIAGAPGFPGARGPSGPQGPSGAPGPKGNSGEPGAPGNKGDAGAKGEPGPVGVQGPP
GPSGEEGKRGSRGEPGPAGLPGPAGERGAPGSRGFPGSDGISGPKGPTGERGSPGPVGPK
GSPGESGRPGEPGLPGAKGLTGSPGSPGPDGKTGPTGPAGQDGRPGAAGPPGSRGPAGVM
GFPGPKGAAGEPGKPGERGVPGPVGAVGAPGKDGEAGAQGPPGAAGPSGERGEQGPSGAP
GFQGLPGPAGPAGESGKSGEQGVPGDAGAPGPAGSRGERXXXXXXGVQGAPGPQGPRGGN
GAPGNDGAKGDAGAPGSPGNQGPPGLQGMPGERGAAGLPGARXXXXXXXXXXXXXXXXXX
XXXGLTGPIGPPGPAGAPGDKGEAGPSGPSGSTGARGAPGDRGEPGPPGPAGFAGPPGAD
GQPGAKGETGDAGAKGDAGAPGPAGPTGAPGPSGAVGAPGPKGARGSAGPPGATGFPGAA
GRVGPPGPSGNIGLPGPPGPSGKXXXXXXXGETGPIGRTGEPGPAGAPGPSGEKGSPGAD
GAPGAPGTPGPQGISGQRGVVGLPGQRGERGFPGLPGPSGEPGKQGPSGSSGERGPPGPV
GPPGLAGPPGEAGREGSPGAEGAPGRDGAAGPKGDRGETGPAGPPGAPGAPGAPGPIGPA
GKNGDRGETGPAGPAGPAGPAGPRGAAGPQGPRXXXXXXXXXXXXXXXXXXGFSGLQGPP
GPPGSPGEQGPSGASGPAGPRGPPGSSGSPGKDGLNGLPGPIGPPGPRXXXXXXXXXXXX
XXXXXXXXXXXXXXXXXXXXXXXXXXXXXXXXXXXXXXXXXXXAADYGQGPMGIMGPRGV
PGTSGPPGAQGFQGPAGEPGEPGQTGPVGARXXXXXXXXSGEDGHPGKPGRXXXXGVPGT
QGARGFPGTPGLPGFKXXXXXXXXXXXXGQAGAPGVKGEPGAHGENGSPGQAGARXXXXX
XXXVGSPGPVGSRGSDGSAGPTGPAGPIGAAGPPGFPGAPGAKGDIGAAGAVGPSGPAGP
RGEPGLPGASGPVGPAGNPGANGIAGAKGAAGLPGVAGAPGLPGPRGIPGPAGPAGTAGA
RGLAGEPGPAGSKGESGNKGEPGAAGSPGPAGPNGEEGKRGSTGEAGATGPPGSAGLRXX
XXXXXXXXXXXXAGSMGLAGSRGSPGPAGARGPGGDSGRPGEPGLLGPRGLPGQPGNPGP
AGKEGPVGFPGADGRVGPIGPAGPRGEPGNIGFPGPKGPNGEPGKPGDKGNVGVAGSRGA
PGPDGNNGVQGPPGVAGNPGAKGEQGPAGPPGFQGLPGPSGPAGEAGKPGDRGIPGEFGL
PGPAGPRXXXGAPGESGAVGPVGSMGSRGPTGPPGSDGNKGEPGNVGAPGGPGPAGPGGI
PGERGIAGVPGGKXXXXXXXXXGEAGATGRXXXXGIPGAVGSPGPAGGPGDRGEAGPAGS
AGPAGPRXXXXXXGESGPAGPNGFAGPPGAAGQTGAKGERXXXGPKGEVGPLGAIGPAGS
SGPAGPIGAAGPAGGRGDAGPSGATGFPGAAGRTGPPGPAGITGPPGPPGQSGKXXXXXX
XGDTGPVGRPGEQGIVGPPGFIGEKGPSGEPGGAGPPGTSGPQGILGAPGILGLPGSRXX
XGLPGISGGTGEPGPAGISGPSGSRGPSGPSGAPGLNGPPGEAGRDGNPGNDGPPGRDGA
PGFKGERGYPGNNGLAGAAGAPGPHGAVGPSGKPGNRGEPGPVGVTGSIGPAGARGLGGP
QGPRXXXGVPGDKGPRXXXXXXXXXXXXXXXXXXXXXXXXXXXXXXXXXXXXGPAGPSGP
HGKDGRNGLPGPIGPAGVRXXXXXXXXXXXXXXXXXXXXXXXXXXXXX--XXXXXXXXXX

>*Natator depressus*
XXXXXXXXXSVGG-LSMPGPMGPAGPRGLPGPPGSPGPQGFQGPPGEPGEPGASGPMGPR
GPAGPPGKNGDDGEAGKPGRPGERGAPGPQGARGLPGTAGLPGMKGHRGFSGLDGAKGDT
GPAGPKGEPGSPGENGAPGQIGPRXXXXXXGRPGPSGSAGARGNDGAPGAAGPTGPAGPA
GPPGFPGAVGAKGETGPQGGRXXXXXXXXXGEPGAPGPAGAAGPTGNPGSDGQPGAKGAT
GAPGIAGAPGFPGARGPSGPQGPSGAPGPKGNSGEPGAPGNKGDAGAKGEPGPVGVQGPP
GPSGEEGKRGSRGEPGPAGLPGPAGERGAPGSRGFPGSDGISGPKGPTGERXXXXXXXXX
GSPGESGRPGEPGLPGAKGLTGSPGSPGPDGKTGPTGPAGQDGRPGAAGPPGSRGPAGVM
GFPGPKGAAGEPGKPGERGVPGPVGAVGAPGKDGEAGAQGPPGAAGPTGERGEQGPSGAP
GFQGLPGPAGPAGESGKXXXXXXXXXXXXXXXXXXXXXXXXXXXXGVQGAPGPQGPRGGN
GAPGNDGAKGDAGAPGSPGNQGPPGLQGMPGERGAAGLPGARGDRGDGGPKXXXXXXXXX
XXXGLTGPIGPPGPAGAPGDKGEAGPSGPSGPTGARGAPGDRGEPGPPGPAGFAGPPGAD
GQPGAKGETGDAGAKGDAGAPGPAGPTGAPGPSGAVGAPGPKGARGSAGPPGATGFPGAA
GRVGPPGPSGNIGLPGPPGPSGKXXXXGPRGETGPIGRXXXXXXXXXXXXXXXXGSPGAD
GAPGAPGTPGPQGISGQRGVVGLPGQRXXXGFPGLPGPSGEPGKXXXXXXXXXXGPPGPV
GPPGLAGPPGEAGREGSPGAEGAPGRDGAAGPKGDRGETGPAGPPGAPGAPGAPGPIGPA
GKNGDRGETGPAGPAGPAGPAGARGAAGPQGPRGDKGETGEHGDRXXXXXXGFSGLQGPP
GPPGSPGEQGPSGASGPAGPRGPPGSSGSPGKDGLNGLPGPIGPPGPRXXXXXXXXXXXX
XXXXXXXXXXXXXXXXXXXXXXXXXXXXXXXXXXXXXXXXXXXAADYGQGPMGIMGPRGA
PGTSGPPGAQGFQGPAGEPGEPGQTGPVGARXXXXXXXXXXXXXXXXXXXXXXXXXXXXX
XXXXGFPGTPGLPGFKXXXGHNGLDGQRGQAGAPGVKGEPGAHGENGSPGQAGARXXXXX
XXXVGSPGPVGSRGSDGSAGPTGPAGPIGAAGPPGFPGAPGAKGDIGAAGAVGPSGPAGP
RGEPGLPGASGPVGPAGNPGANGIAGAKGAAGLPGVAGAPGLPGPRGIPGPAGPAGAAGA
RGLAGEPGPAGSKGESGNKXXXXXXXXXXXXXXXXXXXXXXXXXXXXXXXXXXXXXXXXX
XXXXXXXXXXXXXXXXXXXXXXXXXXXXXXXGPGGDSGRPGEPGLLGPRGLPGQPGNPGP
AGKEGPVGFPGADGRVGPIGPAGARGEPGNIGFPGPKGPNGEPGKPGDKGNVGVAGSRGA
PGPDGNNGVQGPPGVAGNPGAKGEQGPAGPPGFQGLPGPSGPAGEAGKPGDRGIPGEFGL
PGPAGPRXXXGAPGESGAVGPVGSMGSRXXXXXXXXXXXXGEPGNVGAPGGPGPAGPGGI
PGERGIAGVPGGKGEKXXXXXXXXXXXXXXXXXXGIPGAVGSPGPAGGPGDRGEAGPAGS
AGPAGPRXXXXXXGESGPAGPNGFAGPPGAAGQTGAKGERXXXGPKGEVGPLGAIGPAGS
SGPAGPIGAAGPAGGRGDAGPSGATGFPGAAGRTGPPGPAGITGPPGPPGQSGKDGPRXX
XGDTGPVGRPGEQGIVGPPGFTGEKGPSGEPGGAGPPGTSGPQGILGAPGILGLPGSRGE
RGLPGISGGTGEPGPAGISGPSGPRGPSGPSGAPGLNGPPGEAGRDGNPGNDGPPGRDGA
PGFKGERGYPGNNGLAGAAGAPGPHGAVGPSGKPGNRGEPGPVGVTGSIGPAGARXXXXX
XXXXXXXXXXXXXXXXGMPGFKGHNGLQGLPGLAGHHGDQGAPGSTGPAGPRGPAGPSGP
HGKDGRNGLPGPIGPAGVRXXXXXXXXXXXXXXXXXXXXXXXXXXXXX--XXXXXXXXXX

>*Caretta caretta*
XXXXXXXXXSVGG-LSMPGPMGPAGPRGLPGPPGSPGPQGFQGPPGEPGEPGASGPMGPR
GPAGPPGKNGDDGEAGKPGRPGERGAPGPQGARGLPGTAGLPGMKGHRGFSGLDGAKGDT
GPAGPKGEPGSPGENGAPGQIGPRXXXXXXGRPGPSGPAGARGNDGAPGAAGPTGPAGPA
GPPGFPGAVGAKGETGPQGGRXXXXXXXXXXXXXXXXXXXXXXXXXXXXXXXXXXXXGAT
GAPGIAGAPGFPGARGPSGPQGPSGAPGPKGNSGEPGAPGNKGDAGAKGEPGPVGVQGPP
GPSGEEGKRXXXGEPGPAGLPGPAGERXXXXXXGFPGSDGISGPKGPTGERXXXXXXXXX
GSPGESGRPGEPGLPGAKGLTGSPGSPGPDGKTGPTGPAGQDGRPGTAGPPGSRGPAGVM
GFPGPKGAAGEPGKPGERGVPGPVGAVGVPGKDGEAGAQGPPGAAGPSGERGEQGPSGAP
GFQGLPGPAGPAGESGKXXXXXXXXXXXXXXXXXXXXXXXXXXXXGVQGAPGPQGPRGGN
GAPGNDGAKGDAGAPGTPGNQGPPGLQGMPGERGAAGLPGARXXXXXXXXXXXXXXXXXX
XXXGLTGPIGPPGPAGAPGDKGEAGPSGPSGPTGARGAPGDRGEPGPPGPAGFAGPPGAD
GQPGAKGETGDAGAKGDAGAPGPAGPTGAPGPSGAVGAPGPKXXXGSAGPPGATGFPGAA
GRVGPPGPSGNIGLPGPPGPSGKEGTKGPRGETGPVGRTGEPGPVGPPGPSGEKGSPGAD
GAPGAPGTPGPQGISGQRGVVGLPGQRXXXGFPGLPGPSGEPGKQGPSGSSGERGPPGPV
GPPGLAGPAGEAGREGSPGAEGAPGRXXXXXXXGDRGETGPAGPPGAPGAPGAPGPIGPA
GKNGDRGETGPAGPAGPAGPAGARGAAGPQGPRGDKGETGEHGDRXXXXXXGFSGLQGPP
GPPGSPGEQGPSGASGPAGPRGPPGSSGSPGKDGLNGLPGPIGPPGPRXXXXXXXXXXXX
XXXXXXXXXXXXXXXXXXXXXXXXXXXXXXXXXXXXXXXXXXXAADYGQGPMGIMGPRGA
PGTSGPPGAQGFQGPAGEPGEPGQTGPVGARXXXXXXXXXXXXXXXXXXXXXXXXXXXXX
XXXXGFPGTPGLPGFKXXXXXXXXXXXXGQAGAPGVKGEPGAHGENGSPGQAGARXXXXX
XXXIGSPGPVGSRGSDGSAGPTGPAGPIGAAGPPGFPGAPGAKGDIGAAGAVGPSGPAGP
RGEPGLPGASGPVGPAGNPGANGIAGAKGAAGLPGVAGAPGLPGPRGIPGPAGPAGTAGA
RGLAGEPGPAGSKGESGNKGEPGAAGSPGPAGPNGEEGKRGATGEAGATGSPGSAGLRXX
XXXXXXXXXXXXAGSMGPAGSRXXXXXXXXXGPGGDSGRPGEPGLLGPRGLPGQPGNPGP
AGKEGPVGFPGADGRVGPTGAAGPRGEPGNIGFPGPKGPNGEPGKPGDKGNVGVAGSRXX
XXXXXXXXXXXXXXXXXXXXXXGEQGPAGPPGFQGLPGPSGPAGEAGKPGDRGIPGEFGL
PGPAGPRXXXGAPGESGAVGPVGSMGSRGPTGPPGSDGNKGEPGNVGAPGGPGPAGPGGI
PGERGIAGVPGGKGEKXXXXXXGEVGATGRDGSRGIPGAVGSPGPAGGPGDRGEAGPAGS
AGPAGPRXXXXXXGESGPAGPNGFAGPPGAAGQTGAKGERXXXGPKGEVGPLGAIGPAGS
SGPAGPIGAAGPAGGRGDAGPSGATGFPGAAGRTGPPGPAGITGPPGPNGQSGKDGPRXX
XGDTGPVGRPGEQGIVGPPGFTGEKGPSGEPGGAGPPGTSGPQGILGAPGILGLPGSRXX
XGLPGISGGTGEPGPAGISGPSGSRGPSGPSGAPGLNGPPGEAGRDGNPGNDGPPGRDGA
PGFKGERGYPGNNGLAGAAGAPGPHGAVGPSGKPGNRXXXXXXXXXXXXXXXXXXXXXXX
XXXXGEKGVPGDKGPRGMPGFKGHNGLQGLPGLAGHHGDQGAPGSTGPAGPRGPAGPSGP
HGKDGRNGLPGPIGPAGVRXXXXXXXXXXXXXXXXXXXXXXXXXXXXX--XXXXXXXXXX

>*Eretmochelys imbricata*
XXXXXXXXXSVGG-LSMPGPMGPAGPRGLPGPPGSPGPQGFQGPPGEPGEPGASGPMGPR
GPAGPPGKNGDDGEAGKPGRPGERGAPGPQGARGLPGTAGLPGMKGHRGFSGLDGAKGDT
GPAGPKGEPGSPGENGAPGQIGPRXXXXXXGRPGPSGSAGARGNDGAPGAAGPTGPAGPA
GPPGFPGAVGAKGETGPQGGRXXXXXXXXXGEPGAPGPAGAAGPTGNPGSDGQPGAKGAT
GAPGIAGAPGFPGARGPSGPQGPSGAPGPKGNSGEPGAPGNKGDAGAKGEPGPVGVQGPP
GPSGEEGKRXXXGEPGPAGLPGPAGERXXXXXXGFPGSDGISGPKXXXXXXXXXXXXXXX
GSPGESGRPGEPGLPGAKGLTGSPGSPGPDGKTGPTGPAGQDGRPGPAGPPGSRGPAGVM
GFPGPKGAAGEPGKPGERGVPGPVGAVGAPGKDGEAGAQGPPGAAGPSGERGEQGPSGAP
GFQGLPGPAGPAGESGKXXXXXXXXXXXXXXXXXXXXXXXXXXXXGVQGAPGPQGPRGGN
GAPGNDGAKGDAGAPGTPGNQGPPGLQGMPGERGAAGLPGARXXXXXXXXXXXXXXXXX
XXXGLTGPIGPPGPAGASGDKGEAGPSGPSGPTGARGAPGDRGEPGPPGPAGFAGPPGAD
GQPGAKGETGDAGAKGDAGAPGPAGPTGAPGPSGAVGAPGPKGARGSAGPPGATGFPGAA
GRVGPPGPSGNIGLPGPPGPSGKEGTKGPRXXXXXXXXXXXXXXXXXXXXXXXXGSPGAD
GAPGAPGTPGPQGISGQRGVVGLPGQRXXXGFPGLPGPSGEPGKQGPSGSSGERGPPGPV
GPPGLAGPPGEAGREGSPGAEGAPGRXXXXXXXGDRGETGPAGPPGAPGAPGAPGPIGPA
GKNGDRGETGPAGPAGPAGPAGARGAAGPQGPRGDKGETGEHGDRXXXXXXGFSGLQGPP
GPPGSPGEQGPSGASGPAGPRGPPGSSGSPGKDGLNGLPGPIGPPGPRXXXXXXXXXXXX
XXXXXXXXXXXXXXXXXXXXXXXXXXXXXXXXXXXXXQYDPSKAADYGQGPMGIMGPRGA
PGTSGPPGAQGFQGPAGEPGEPGQTGPVGARXXXXXXXXTGEDGHPGKPGRXXXXGVPGS
QGARGFPGTPGLPGFKXXXGHNGLDGQRGQAGAPGVKGEPGAHGENGSPGQAGARXXXXX
XGRIGSPGPVGSRGSDGSAGPTGPAGPIGAAGPPGFPGAPGAKGDIGAAGAVGPSGPAGP
RGEPGLPGASGPVGPAGNPGANGIAGAKGAAGLPGVAGAPGLPGPRGIPGPAGPAGAAGA
RGLAGEPGPAGSKGESGNKGEPGAAGPPGPAGPNGEEGKRGTTGEAGATGPPGSAGLRXX
XXXXXXXXXXXXXXXXXXXXXXXXXXXXXXXGPGGDTGRPGEPGLLGPRGLPGQPGNPGP
AGKEGPVGFPGADGRVGPSGPAGPRGEPGNIGFPGPKGPNGEPGKPGDKGNVGVAGSRGA
PGPDGNNGVQGPPGVAGNPGAKGEQGPAGPPGFQGLPGPSGPAGEAGKPGDRGIPGEFGL
PGSAGPRXXXGAPGESGAVGPVGSMGSRXXXXXXXXXXXXGEPGNVGAPGGPGPAGPGGI
PGERGIAGVPGGKGEKXXXXXXGEVGATGRXXXXGIPGAVGSPGPAGGPGDRGEAGPAGS
AGPAGPRXXXXXXGESGPAGPNGFAGPPGAAGQTGAKGERXXXGPKGEVGPLGAIGPAGS
SGPAGPIGAAGPTGGRGDAGPSGATGFPGAAGRTGPPGPAGITGPPGPPGQSGKXXXXXX
XGDTGPVGRPGEQGIVGPPGFTGEKGPSGEPGGAGPPGTSGPQGILGAPGILGLPGSRXX
XGLPGISGGTGEPGPAGISGPSGSRGPSGPSGASGLNGPPGEAGRDGNPGNDGPPGRDGA
PGFKGERGYPGNNGLAGAAGAPGPHGAVGPSGKPGNRGEPGPVGVTGSVGPAGARXXXXX
XXXXXXXGVPGDKGPRXXXXXXGHNGLQGLPGLAGHHGDQGAPGSTGPAGPRGPAGPSGP
HGKDGRNGLPGPIGPAGVRXXXXXXXXXXXXXXXXXXXXXXXXXXXX--XXXXXXXXXX

>Lepidochelys_kempii
XXXXXXXXXSVGG-LSMPGPMGPAGPRGLPGPPGSPGPQGFQGPPGEPGEPGASGPMGPR
XXXXXXXXXXXXXXXXXXXXXXXXGAPGPQGARGLPGTAGLPGMKGHRGFSGLDGAKGDT
GPAGPKGEPGSPGENGAPGQIGPRXXXXXXGRPGPSGSAGARGNDGAPGAAGPTGPAGPA
GPPGFPGAVGAKGETGPQGGRGGEGPQGTRGEPGAPGPAGAAGPTGNPGSDGQPGAKGAT
GAPGIAGAPGFPGARGPSGPQGPSGAPGKXXXXXXXXXXXXXGDAGAKGEPGPVGVQGPP
GPSGEEGKRGSRGEPGPAGLPGPAGERXXXXXXGFPGSDGISGPKGPSGERXXXXXXXXX
GSPGESGRPGEPGLPGAKGLTGSPGSPGPDGKTGPTGPAGQDGRPGAAGPPGSRGPAGVM
GFPGPKGAAGEPGKPGERGVPGPVGAVGAPGKDGEAGAQGPPGAAGPSGERGEQGPSGAP
GFQGLPGPAGPAGESGKXXXXXXXXXXXXXXXXXXXXXXGFPGERGVQGAPGLQGPRGGN
GAPGNDGAKGDAGAPGTPGNQGPPGLQGMPGERGAAGLPGARXXXXXXXXXXXXXXXXXX
XXXGLTGPIGPPGPAGAPGDKGEAGPSGASGPTGARGAPGDRGEPGPPGPAGFAGPPGAD
GQPGAKGETGDAGAKGDAGAPGPAGPTGAPGPSGAVGAPGPKGARGSAGPPGATGFPGAA
GRVGPPGPSGNIGLPGPPGPSGKXXXXXXXGETGPVGRTGEPGPTGPPGPSGEKGSPGAD
GAPGAPGTPGPQGISGQRGVVGLPGQRGERGFPGLPGPSGEPGKQGPSGSSGERGPPGPV
GPPGLAGPPGEAGREGSPGAEGAPGRXXXXXXXXXXGETGPAGPPGAPGAPGAPGPIGPA
GKXXXXGETGPAGPAGPAGPAGARGAAGPQGPRXXXXXXXXXXXXXXXXXXGFSGLQGPP
GPPGSPGEQGPSGASGPAGPRGPPGSSGSPGKDGLNGLPGPIGPPGPRXXXXXXXXXXXX
XXXXXXXXXXXXXXXXXXXXXXXXXXXXXXXXXXXXXXXXXXXAADYGQGPMGIMGPRGA
PGTSGPPGAQGFQGPAGEPGEPGQTGPVGARXXXXXXXXXXXXXXXXXXXXXXXXXXXXX
XXXXGFPGTPGLPGFKXXXGHNGLDGQRGQAGAPGVKGEPGAHGENGSPGQAGARXXXXX
XXXIGSPGPVGSRGSDGSAGPTGPAGPIGAAGPPGFPGAPGAKGDIGAAGAVGPSGPAGP
RGEPGLPGASGPVGPAGNPGANGIAGAKGAAGLPGVAGAPGLPGPRGIPGPAGPAGTAGA
RGLAGEPGPAGSKGESGNKGEPGAAGAPGPAGPNGEEGKRGSTGEAGATGSPGPAGLRXX
XXXXXXXXXXXXGGSMGLAGSRGSPGPAGARGPGGDSGRPGEPGLLGPRGLPGQPGNPGP
AGKEGPVGFPGADGRVGPTGAAGPRGEPGNIGFPGPKXXXXXXXXXXXXGHVGVAGSRXX
XXXXXXXXXXXXXXXXXXXXXXGEQGPAGPPGFQGLPGPSGPAGEAGKPGDRGIPGEFGL
PGPAGPRXXXGAPGESGAVGPVGSMGSRXXXXXXXXXXXXGEPGNVGAPGGPGPAGPGGI
PGERGIAGVPGGKGEKGLPGLRGEVGATGRXXXXGIPGAVGSPGPAGGPGDRGEAGPAGS
TGPAGPRGAPGERGESGPAGPNGFAGPPGAAGQTGAKGERXXXGPKGEVGPLGAIGPAGS
SGPAGPIGAAGPAGGRGDAGPSGATGFPGAAGRTGPPGPAGITGPPGPPGQSGKXXXXXX
XGDTGPVGRPGEQGIVGPPGFTGEKGPSGEPGGAGPPGTSGPQGILGAPGILGLPGSRXX
XGLPGISGGTGEPGPAGISGPSGSRGPSGPSGAPGLNGPPGEAGRDGNPGNDGPPGRDGA
PGFKGERXXXXXXXXXXXXXXXXXXXXXXXXXXPGNRGEPGPVGVTGSVGPAGARXXXXX
XXXXXXXXXXXXXXXXXXXXXXGHNGLQGLPGLAGHHGDQGASGSTGPAGPRGPAGPSGP
HGKDGRNGLPGPIGPAGVRXXXXXXXXXXXXXXXXXXXXXXXXXXXXX--XXXXXXXXXX

>*Lepidochelys_olivacea*
XXXXXXXXXSVGG-LSMPGPMGPAGPRXXXXXXXXXXXXXXXXXXXXXXXXXXXXXXXXX
GPAGPPGKNGDDGEAGKPGRPGERGAPGPQGARGLPGTAGLPGMKGHRGFSGLDGAKGDT
GPAGPKGEPGSPGENGAPGQIGPRXXXXXXGRPGPSGSAGARGNDGAPGAAGPTGPAGPA
GPPGFPGAVGAKGETGPQGGRGGEGPQGTRGEPGAPGPAGAAGPTGNPGSDGQPGAKGAT
GAPGIAGAPGFPGARGPSGPQGPSGAPGPKGNSGEPGAPGNKGDAGAKGEPGPVGVQGPP
GPSGEEGKRXXXGEPGPAGLPGPAGERXXXXXXGFPGSDGISGPKGPSGERXXXXXXXXX
GSPGESGRPGEPGLPGAKGLTGSPGSPGPDGKTGPTGPAGQDGRPGAAGPPGSRGPAGVM
GFPGPKGAAGEPGKPGERGVPGPVGAVGAPGKDGEAGAQGPPGAAGPSGERGEQGPSGAP
GFQGLPGPAGPAGESGKXXXXXXXXXXXXXXXXXXXXXXXXXXXXGVQGAPGLQGPRGGN
GAPGNDGAKGDAGAPGTPGNQGPPGLQGMPGERGAAGLPGARGDRGDGGPKGADGAPGKX
XXXGLTGPIGPPGPAGAPGDKGEAGPSGASGPTGARGAPGDRGEPGPPGPAGFAGPPGAD
GQPGAKGETGDAGAKGDAGAPGPAGPTGAPGPSGAVGAPGPKXXXGSAGPPGATGFPGAA
GRVGPPGPSGNIGLPGPPGPSGKEGTKGPRGETGPVGRTGEPGPTGPPGPSGEKGSPGAD
GAPGAPGTPGPQGISGQRGVVGLPGQRXXXGFPGLPGPSGEPGKQGPSGSSGERGPPGPV
GPPGLAGPPGEAGREGSPGAEGAPGRXXXXXXXXXXXXXXXXXXXXXXXXXXXXXXXXXX
XXNGDRGETGPAGPAGPAGPAGARGAAGPQGPRGDKGETGEHGDRXXXXXXGFSGLQGPP
GPPGSPGEQGPSGASGPAGPRGPPGSSGSPGKDGLNGLPGPIGPPGPRXXXXXXXXXXXX
XXXXXXXXXXXXXXXXXXXXXXXXXXXXXXXXXXXXXXXXXXXAADYGQGPMGIMGPRGA
PGTSGPPGAQGFQGPAGEPGEPGQTGPVGARXXXXXXXXXXXXXXXXXXXXXXXXXXXXX
XXXXGFPGTPGLPGFKXXXGHNGLDGQRGQAGAPGVKXXXXXXXXXXXXXXXXXXXXXXX
XXXIGSPGPVGSRXXXXXXXXXXXXXXXXXXXXXXXXXXXXXXGDIGAAGAVGPSGPAGP
RGEPGLPGASGPVGPAGNPGANGIAGAKGAAGLPGVAGAPGLPGPRGIPGPAGPAGTAGA
RGLAGEPGPAGSKGESGNKGEPGAAGAPGPAGPNGEEGKRGSTGEAGATGSPGPAGLRXX
XXXXXXXXXXXXGGSMGLAGSRXXXXXXXXXGPGGDSGRPGEPGLLGPRGLPGQPGNPGP
AGKEGPVGFPGADGRVGPTGAAGPRGEPGNIGFPGPKGPNGEPGKPGDKGHVGVAGSRXX
XXXXXXXXXXXXXXXXXXXXXXGEQGPAGPPGFQGLPGPSGPAGEAGKPGDRGIPGEFGL
PGPAGPRXXXGAPGESGAVGPVGSMGSRXXXXXXXXXXXXGEPGNVGAPGGPGPAGPGGI
PGERGIAGVPGGKGEKXXXXXXGEVGATGRXXXXGIPGAVGSPGPAGGPGDRGEAGPAGS
TGPAGPRXXXXXXGESGPAGPNGFAGPPGAAGQTGAKGERGQKGPKGEVGPLGAIGPAGS
SGPAGPIGAAGPAGGRGDAGPSGATGFPGAAGRTGPPGPAGITGPPGPPGQSGKXXXXXX
XGDTGPVGRPGEQGIVGPPGFTGEKGPSGEPGGAGPPGTSGPQGILGAPGILGLPGSRXX
XGLPGISGGTGEPGPAGISGPSGSRGPSGPSGAPGLNGPPGEAGRDGNPGNDGPPGRDGA
PGFKGERXXXXXXXXXXXXXXXXXXXXXXXXXXXXXXGEPGPVGVTGSVGPAGARGLVGP
QGPRGEKGVPGDKGPRXXXXXXGHNGLQGLPGLAGHHGDQGASGSTGPAGPRGPAGPSGP
HGKDGRNGLPGPIGPAGVRGSQGSQGPSGPPGPPGHPGPDGPNGGGY--EIGYDAEYYR

>Dermochelys_coriacea
XXXXXXXXXSVGG-LSMPGPMGPAGPRGLPGPPGSPGPQGFQGPPGEPGEPGASGPMGPR
XXXXXXXXNGDDGEAGKPGRXXXXGAPGPQGARGLPGTAGLPGMKXXXGFSGLDGAKGDT
GPAGPKGEPGSPGENGAPGQIGPRXXXXXXGRPGPSGSAGARGNDGAPGAAGPTGPVGPA
GPPGFPGAVGVKXXXXXXXXXXGGEGPQGARGEPGAPGPAGAAGPAGNPGSDGQPGAKGAT
GAPGIAGAPGFPGARGPSGPQGPSGAPGPKGNSGEPGAPGNKGDAGAKGEPGPVGVQGPP
GPSGEEGKRXXXGEPGPAGLPGPAGERGAPGSRGFPGSDGISGPKGPSGERGSPGPAGPK
GSPGESGRPGEPGLPGAKGLTGSPGSPGPDGKTGPTGPAGQDGRPGPAGPPGARGQAGVM
GFPGPKGAAGEPGKPGERGVPGPVGAVGALGKDGEAGAQGPPGAAGPSGERXXXXXXXXX
XXXXXXXXXXXXXXXXXXXXXXXXXXXXXXXXXXXXXXXXXXXGVQGAPGPQGPRGSN
GAPGNDGAKGDAGAPGSPGNQGPPGLQGMPGERGAAGLPGARGDRGDGGPKGADGAPGKX
XXXGLTGPIGPPGPAGAPGDKGEAGPSGAPGPTGARGAPGDRGEPGPPGPAGFAGPPGAD
GQPGAKGETGDAGAKGDAGAPGPAGPTGAPGPSGAVGAPGPKXXXGSAGPPGATGFPGAA
GRVGPPGPSGNIGLPGPPGPSGKXXXXGPRGETGPAGRTGEPGPAGPPGPSGEKGSPGAD
GAPGAPGLPGPQGISGQRGVVGLPGQRGERGFPGLPGPSGEPGKQGPSGSSGERXXXGPA
GPPGLAGPPGEAGREGSPGAEGAPGRDGAAGPKGDRGETGPAGPPGAPGAPGAPGPVGPA
GKNGDRGETGPAGPAGPAGPAGARGAAGPQGPRGDKGETGEHGDRXXXXXXGFSGLQGPP
GPPGSPGEQGPSGASGPAGPRGPPGSSGSPGKDGLNGLPGPIGPPGPRXXXXXXXXXXXX
XXXXXXXXXXXXXXXXXXXXXXXXXXXXXXXXXXXXQYDPSKAADYGQGPMGIMGPRGA
PGTSGPPGAQGFQGPAGEPGEPGQTGPVGARGPAGPPGKXXXXXXXXXXXXXXXXXXXXX
XXXXGFPGTPGLPGFKXXXGHNGLDGQRGQAGAPGVKGEPGAHGENGSPGQAGARXXXXX
XGRVGSPGPVGSRGSDGSAGPTGPAGPIGAAGPPGFPGAPGAKGDIGAAGAVGPSGPAGP
RGEPGLPGASGPVGPAGNPGANGIAGAKGAAGLPGVAGAPGLPGPRGIPGPAGPAGTAGA
RGLAGEPGPAGSKGESGNKXXXXXXXXXXXXXXXXXXXXXRGPTGEAGATGSPGPAGLRXX
XXXXXXXXXXXXAGGMGPAGSRXXXXXXXXXXXXXXXXXXPGEPGLLGPRGLPGQPGNPGP
AGKEGPVGFPGADGRVGPTGPAGVRGEPGNIGFPGPKGPNGEPGKPGDKGNVGVAGLRGA
PGPDGNNGVQGPPGVAGNPGAKGEQGPAGPPGFQGLPGPSGPAGEAGKPGDRGIPGEFGL
PGPAGPRXXXGAPGESGAAGPVGSMGSRXXXXXXXXXXXXXGEPGNAGAPGGPGPAGSGGI
PGERXXXXXXXXXXXXXXXXXXXGEAGATGRXXXXGIPGAVGSPGPAGGPGDRGEAGPAGS
AGPAGVRXXXXXXGESGPAGPNGFAGPPGAAGQTGAKGERXXXGPKGELGPLGAIGPAGN
SGPAGPIGAAGPAGARGDAGPSGATGFPGAAGRTGPPGPAGITGPPGPPGQSGKXXXXXX
XGDTGPVGRPGEQGIVGPAGFIGEKGPSGEPGGVGPPGTSGAQGILGAPGILGLPGSRGE
RGLPGISGGTGEPGPVGISGPSGSRGPSGPSGAPGLPGPPGEAGRDGNPGNDGPPGRDGA
PGFKGERGYPGSNGPAGAAGAPGPHGAVGPSGKPGMRGETGPVGVTGPIGPAGARGLAGP
QGPRXXXGVPGDKGPRXXXXXXGHNGLQGLPGLAGHHGDQGAPGSTGPAGPRGPAGPSGP
HGKDGRNGLPGPIGPAGVRGSQGSQGPSGPPGPPGHPGPPGPNGGGY--EIGYDAEYYR

Supplementary Table S4.2: Matrix displaying the total number of collagen (I) amino acid substitutions across the α1 and α2 chains of the species of Testudines listed.

Common name	Latin name		1	2	3	4	5	6	7	8	9	10
Green	*Chelonia mydas*	1										
Green	*Chelonia mydas* (Pacific)	2	0									
Green	*Chelonia mydas* (Atlantic)	3	0	0								
New species?	*Chelonia* sp. (Ancient)	4	1	1	1							
Flatback	*Natator depressus*	5	7	7	7	8						
Loggerhead	*Caretta caretta*	6	20	18	19	20	14					
Hawksbill	*Eretmochelys imbricata*	7	20	18	19	20	13	16				
Kemp's ridley	*Lepidochelys kempii*	8	23	22	23	22	16	17	18			
Olive ridley	*Lepidochelys olivacea*	9	26	24	24	23	16	17	18	0		
Leatherback	*Dermochelys coriacea*	10	46	46	45	46	39	44	45	46	46	
Softshell turtle	*Pelodiscus sinensis*	11	149	145	145	133	109	124	124	123	121	115

Supplementary Figure S1.1: Collagen fingerprints from reference sample of *Chelonia mydas* (green sea turtle; sample FLMNH 57247 from Vietnam, Pacific Ocean) following digestion with trypsin and fractionation into 10% ACN (top) and 50% ACN (bottom).

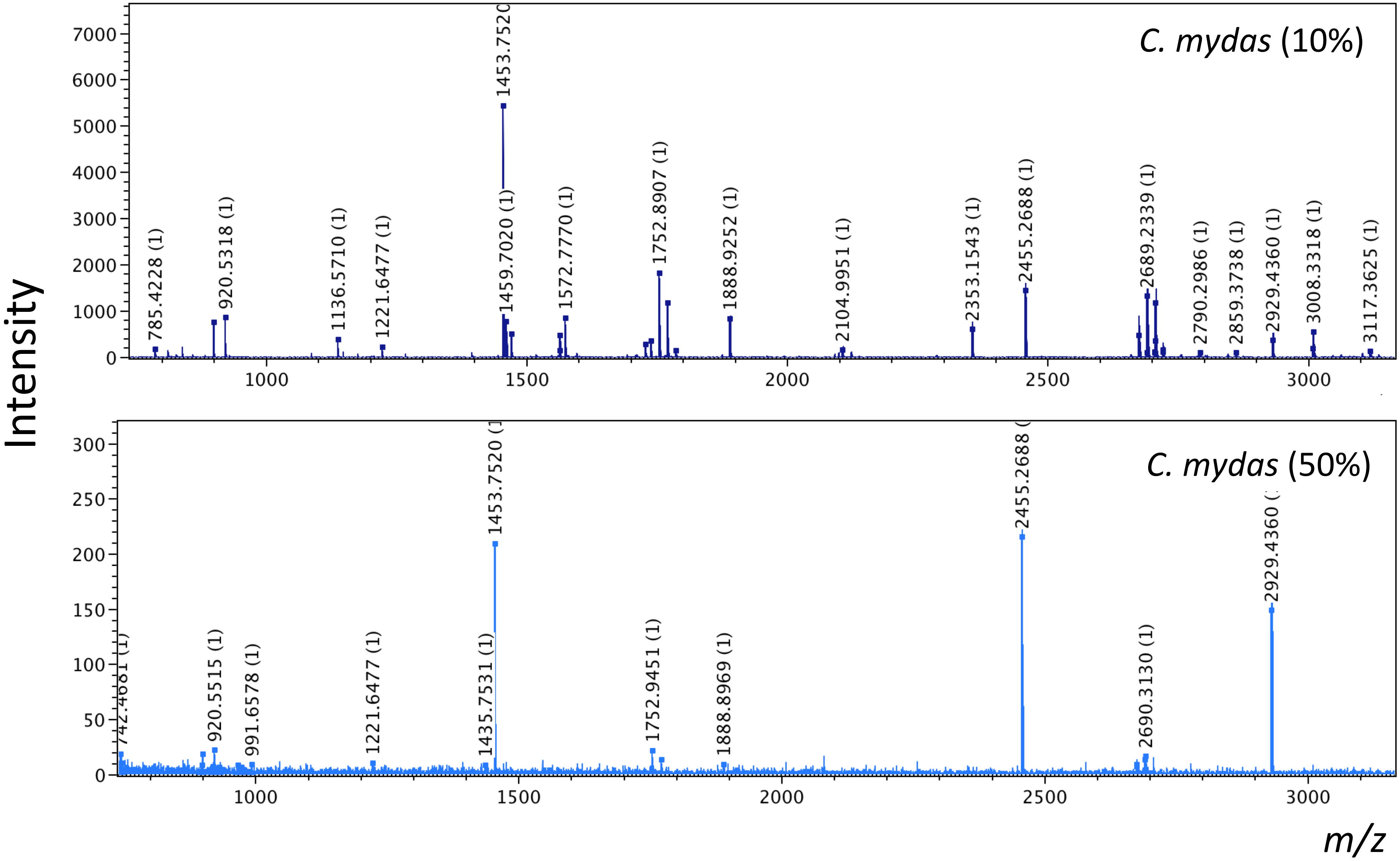

Supplementary Figure S1.2: Collagen fingerprints from reference sample of *Chelonia mydas* (green sea turtle; sample UF42972 from Florida, Atlantic Ocean) following digestion with trypsin and fractionation into 10% ACN (top) and 50% ACN (bottom).

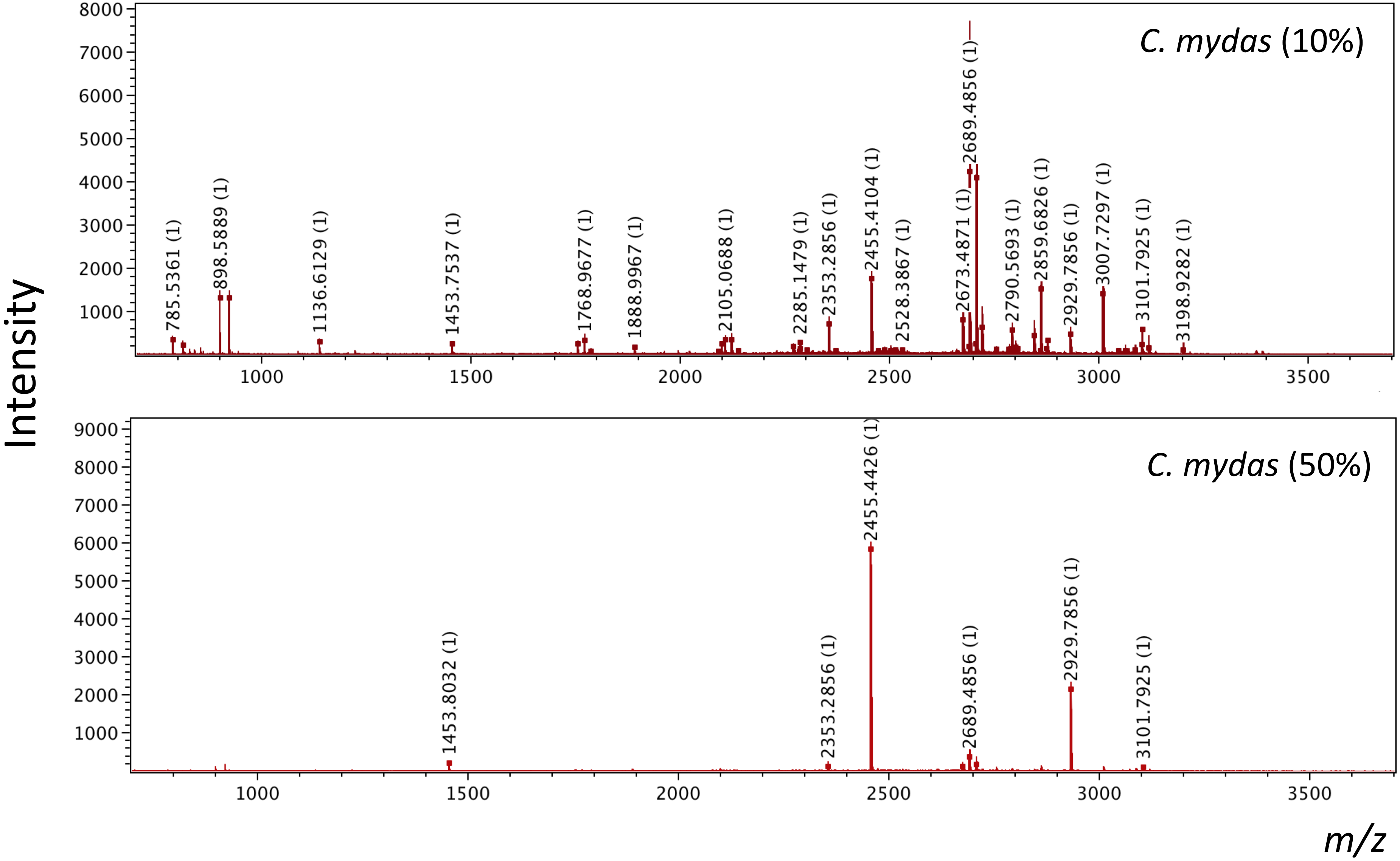

Supplementary Figure S1.3: Collagen fingerprints from reference sample of *Chelonia mydas* (green sea turtle; sample UF52003 from Sonora, Mexico, Pacific Ocean) following digestion with trypsin and fractionation into 10% ACN (top) and 50% ACN (bottom).

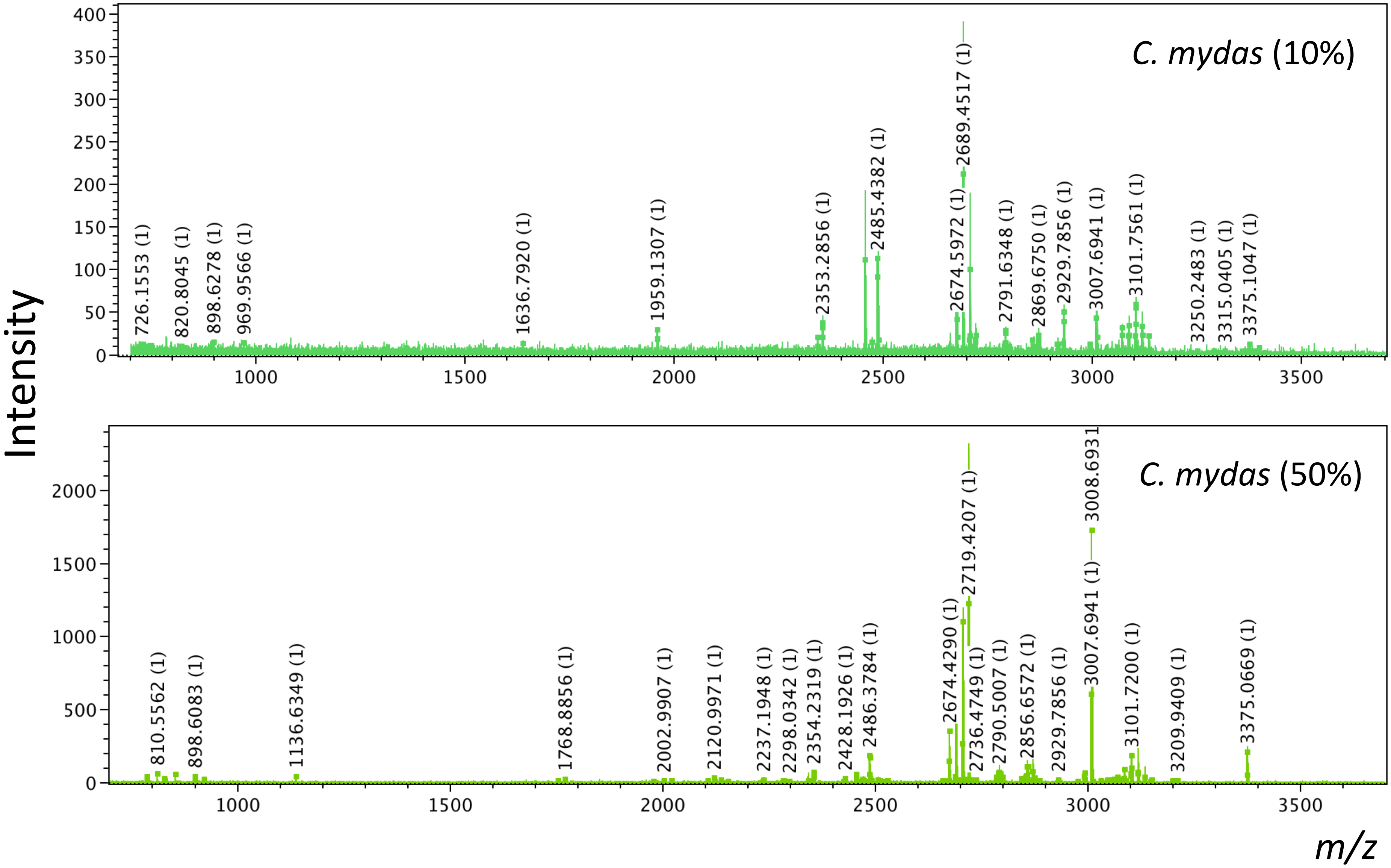

Supplementary Figure S1.4: Collagen fingerprints from reference sample of *Caretta caretta* (loggerhead sea turtle) following digestion with trypsin and fractionation into 10% ACN (top) and 50% ACN (bottom).

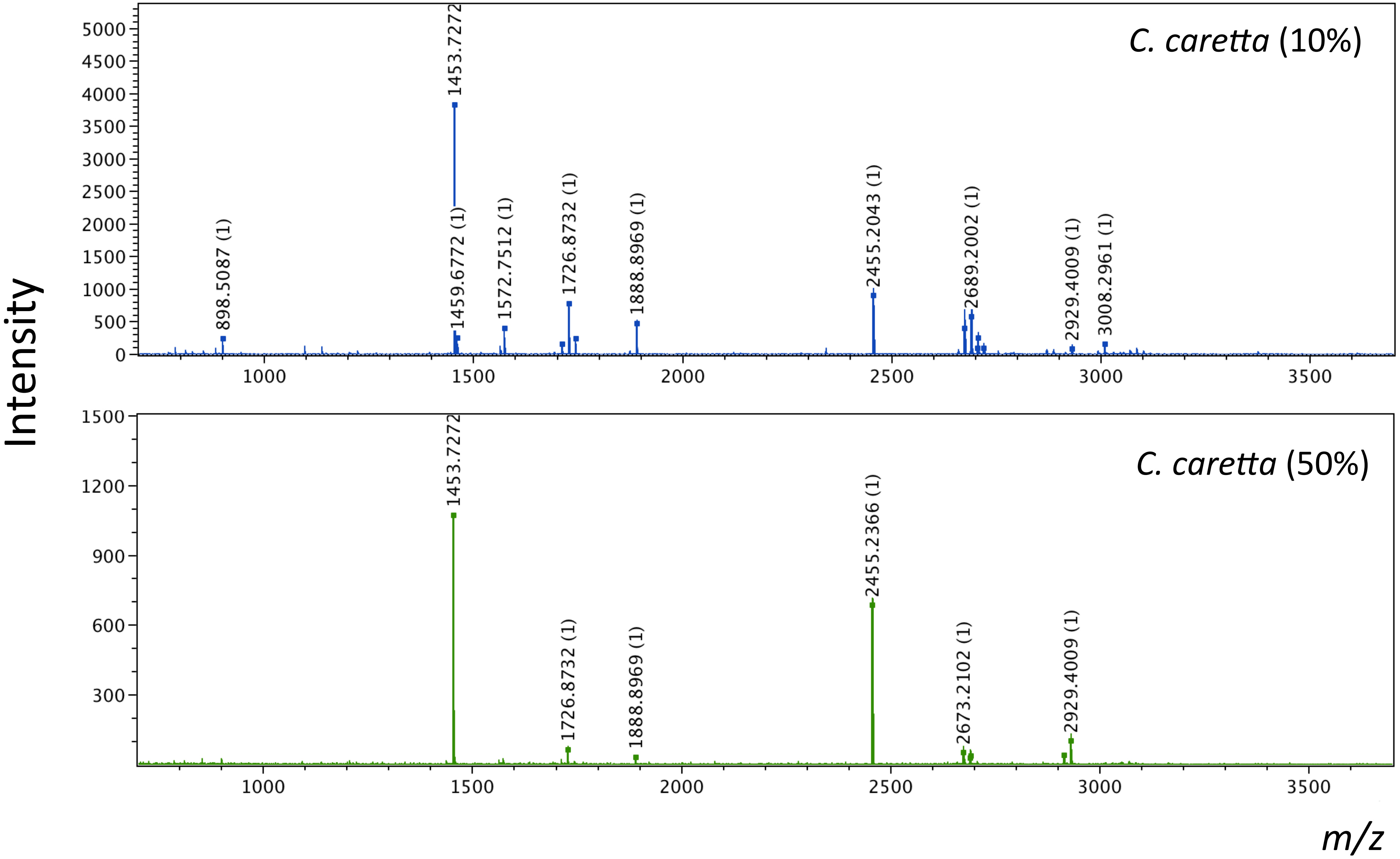

Supplementary Figure S1.5: Collagen fingerprints from reference sample of *Eretmochelys imbricata* (hawksbill sea turtle) following digestion with trypsin and fractionation into 10% ACN (top) and 50% ACN (bottom).

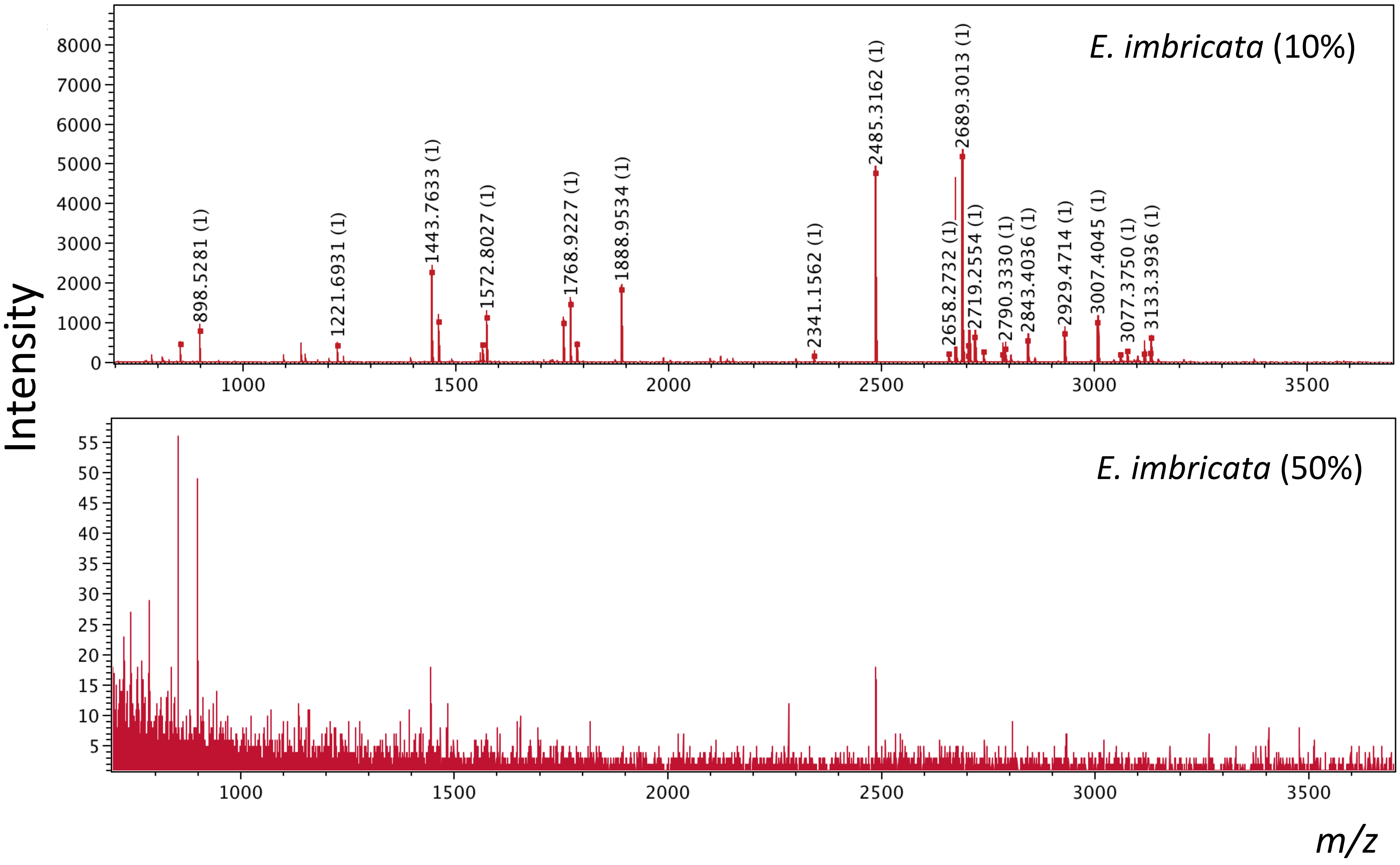

Supplementary Figure S1.6: Collagen fingerprints from reference sample of *Lepidochelys kempii* (Kemp's ridley sea turtle) following digestion with trypsin and fractionation into 10% ACN (top) and 50% ACN (bottom).

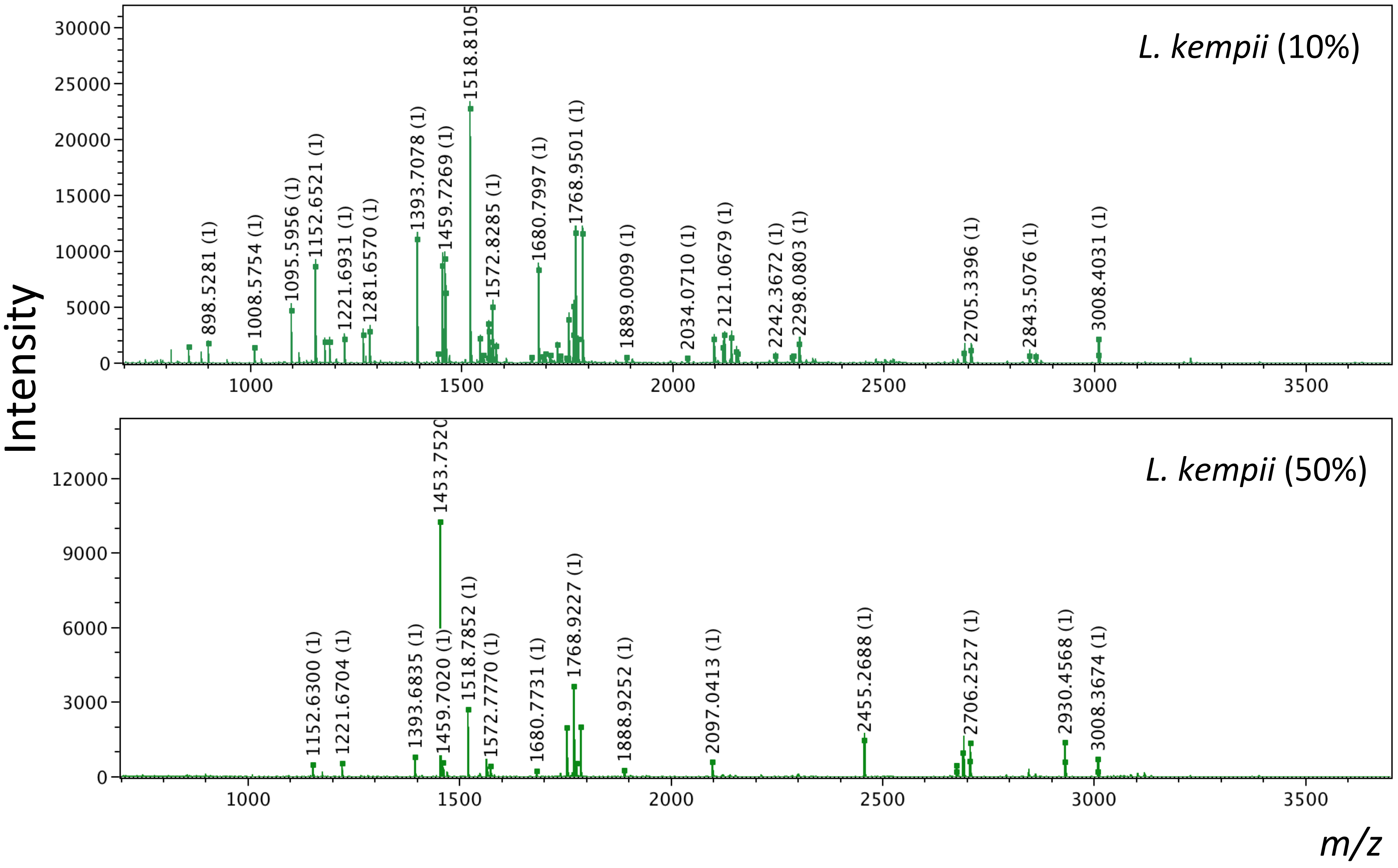

Supplementary Figure S1.7: Collagen fingerprints from reference sample of *Lepidochelys olivacea* (olive ridley sea turtle) following digestion with trypsin and fractionation into 10% ACN (top) and 50% ACN (bottom).

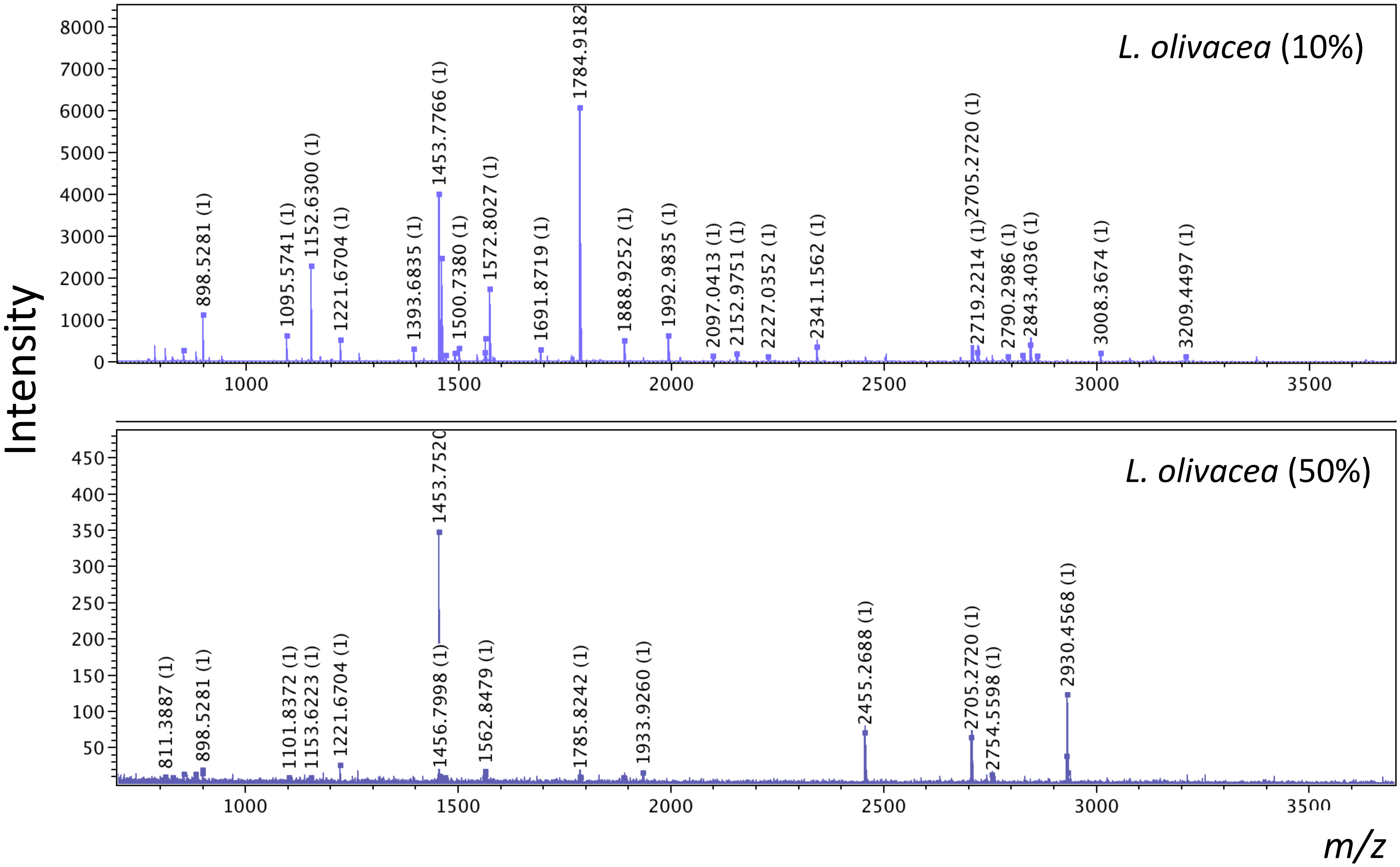

Supplementary Figure S1.8: Collagen fingerprints from reference sample of *Dermochelys coriacea* (leatherback sea turtle) following digestion with trypsin and fractionation into 10% ACN (top) and 50% ACN (bottom).

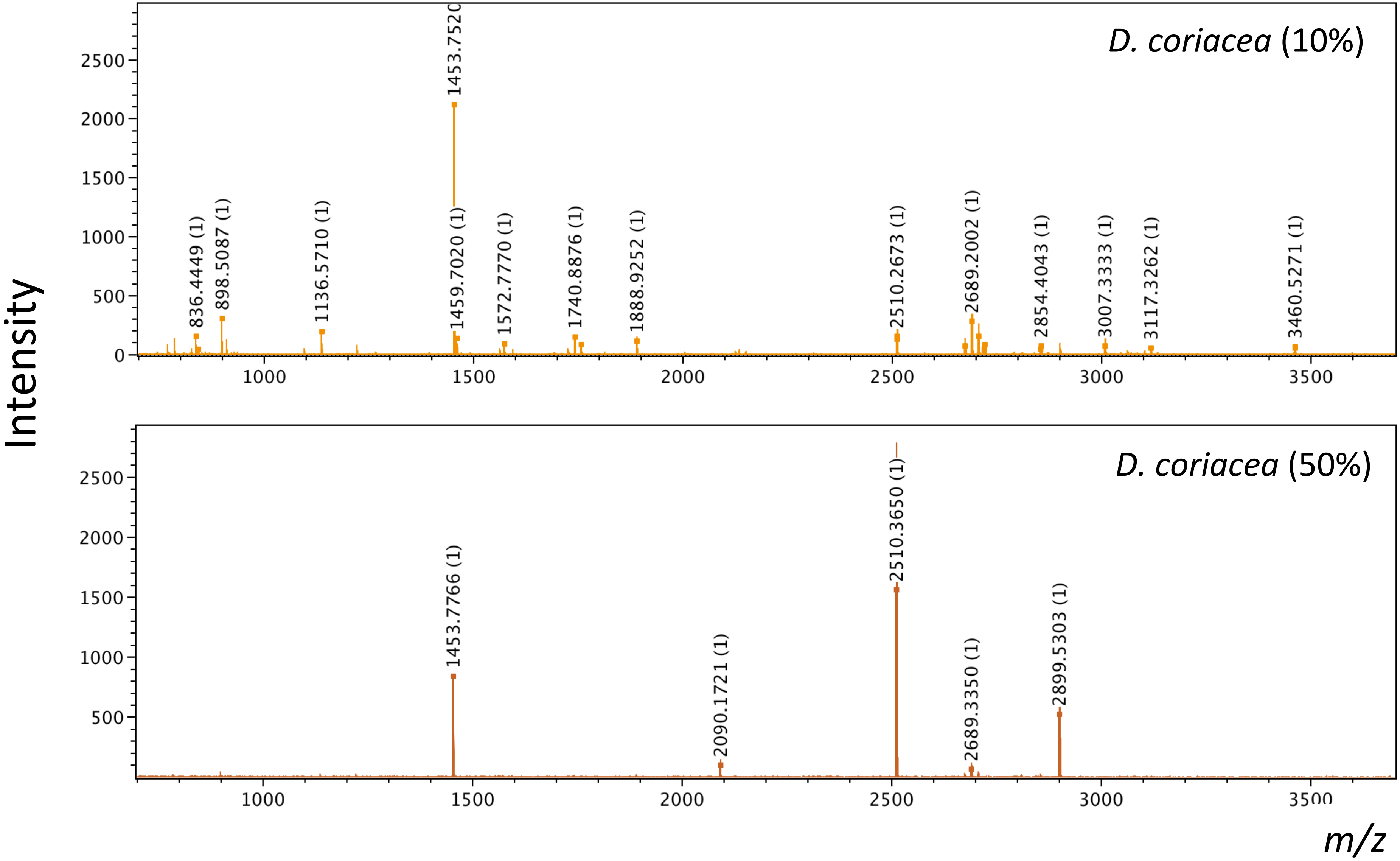

Supplementary Figure S1.9: Collagen fingerprints from reference sample of *Natator depressus* (flatback sea turtle) following digestion with trypsin and fractionation into 10% ACN (top) and 50% ACN (bottom).

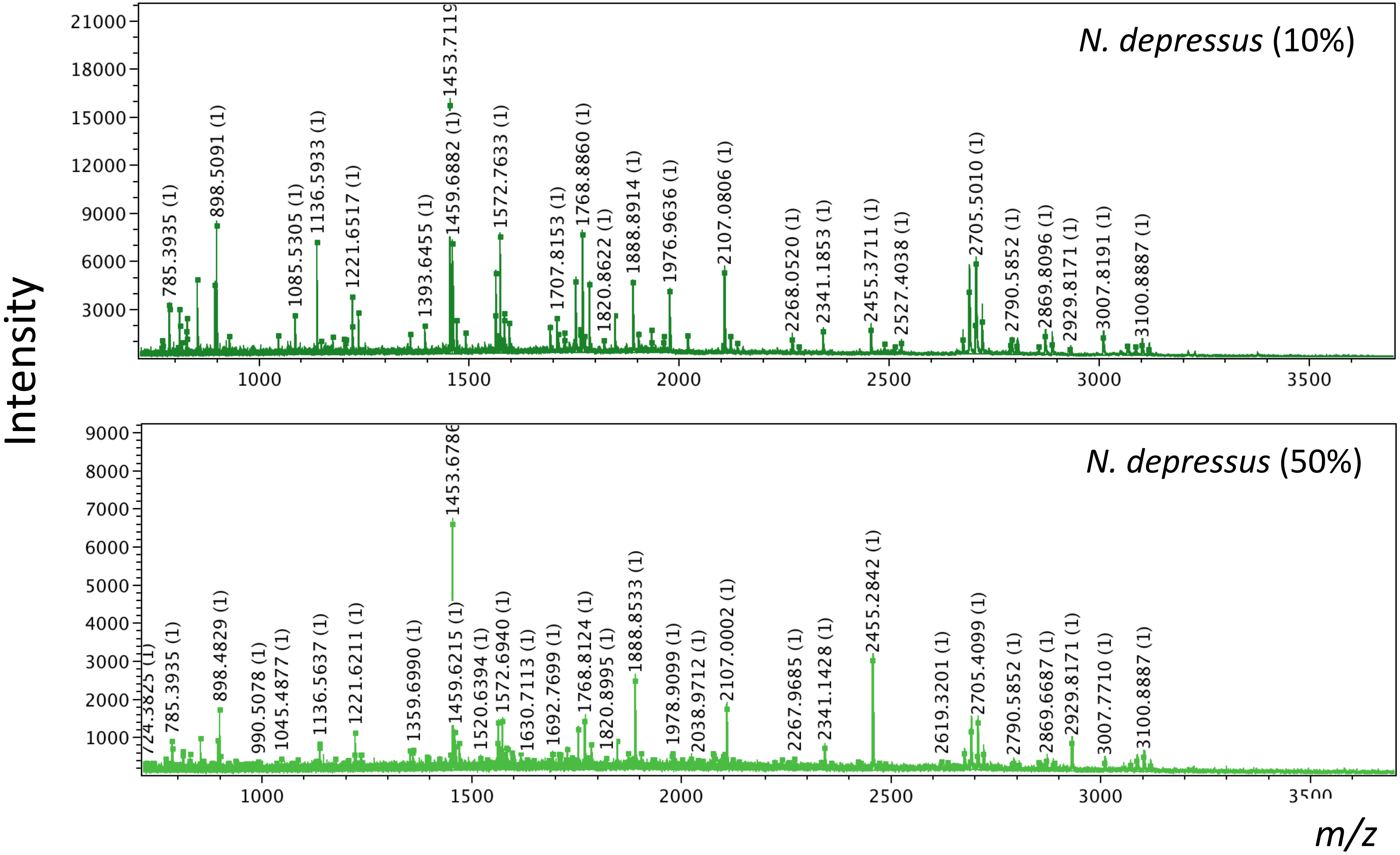

Supplementary Figure S1.10: Collagen fingerprints from reference sample of *Macrochelys suwanniensis* (Suwanee snapping turtle) (Family: Chelydridae) following digestion with trypsin and fractionation into 10% ACN (top) and 50% ACN (bottom).

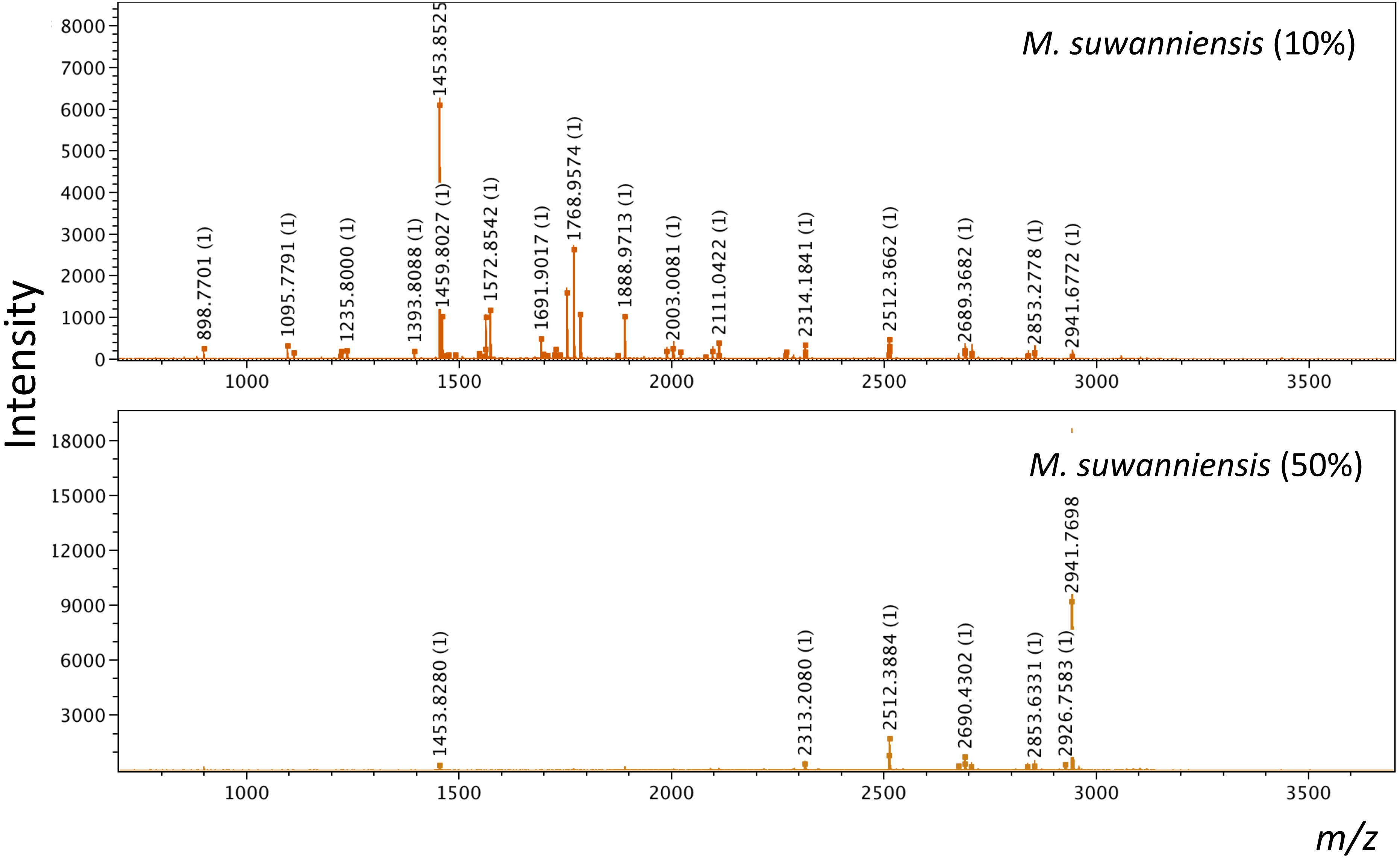

Supplementary Figure S1.11: Collagen fingerprints from reference sample of *Deirochelys reticularia* (chicken turtle) (Family: Emydidae) following digestion with trypsin and fractionation into 10% ACN (top) and 50% ACN (bottom).

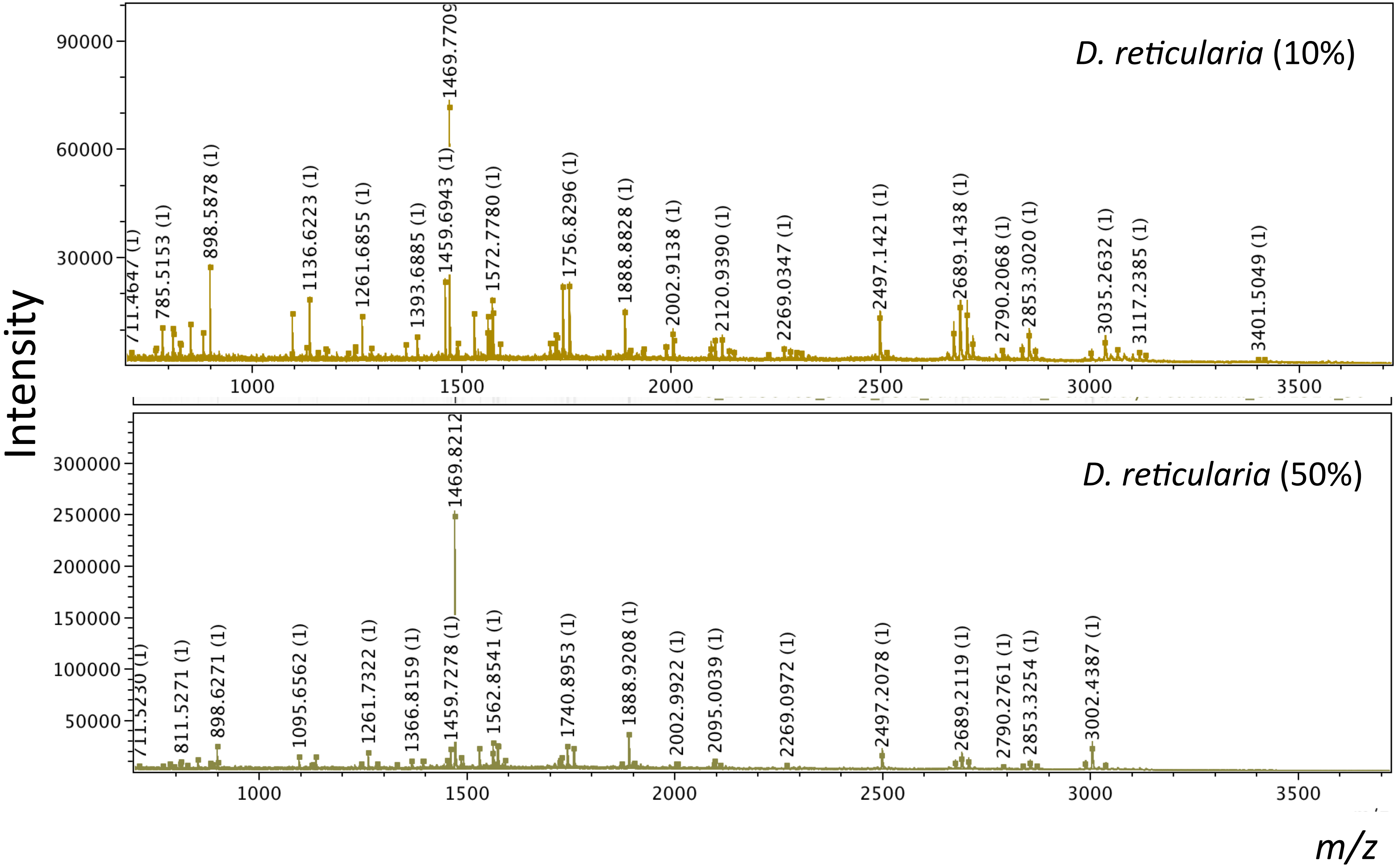

Supplementary Figure S1.12: Collagen fingerprints from reference sample of *Gopherus polyphemus* (gopher tortoise) (Family: Testudinidae) following digestion with trypsin and fractionation into 10% ACN (top) and 50% ACN (bottom).

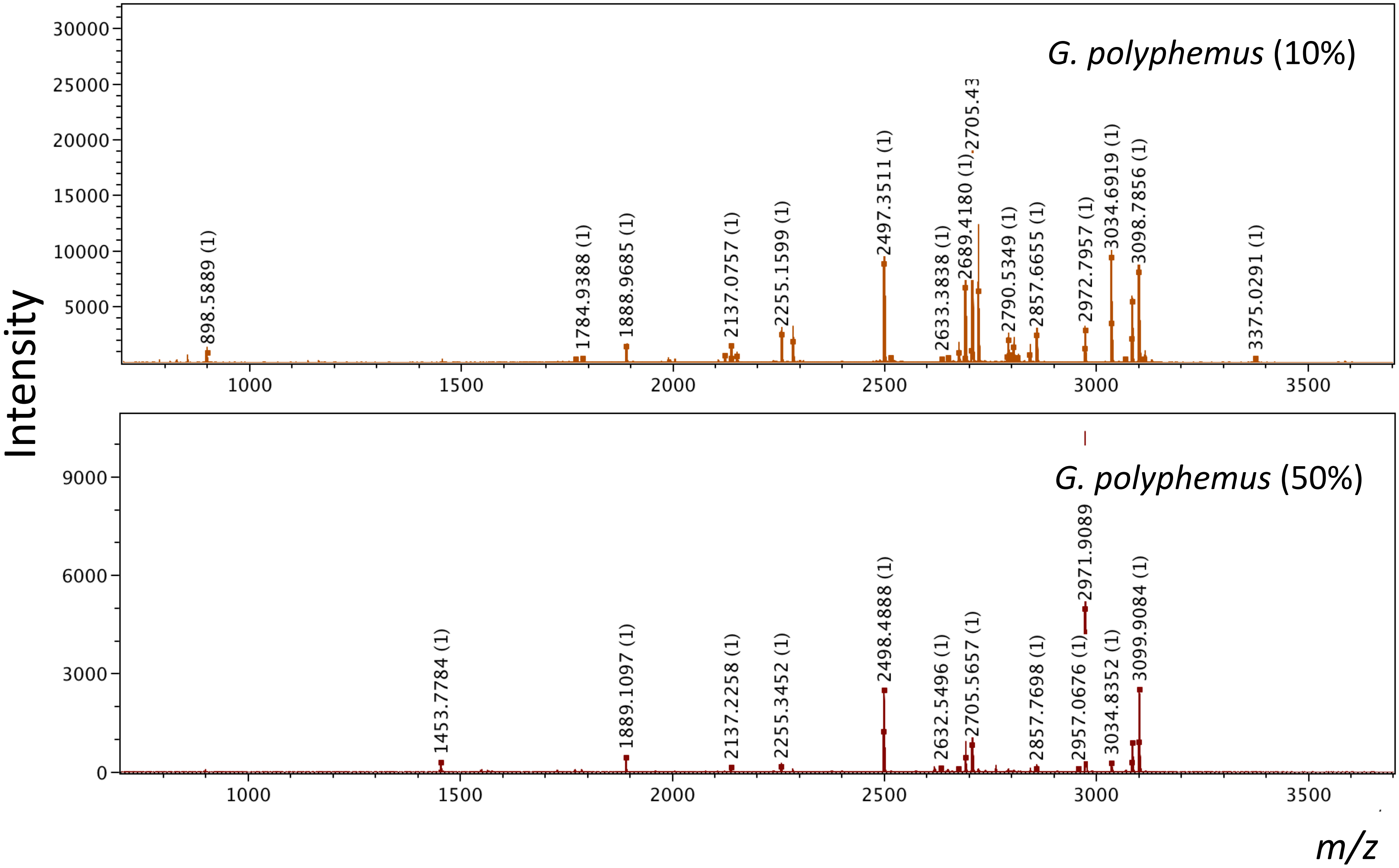

Supplementary Figure S1.13: Collagen fingerprint from a representative ancient sample of *Chelonia mydas* (green sea turtle) (Sample GP10) following digestion with trypsin.

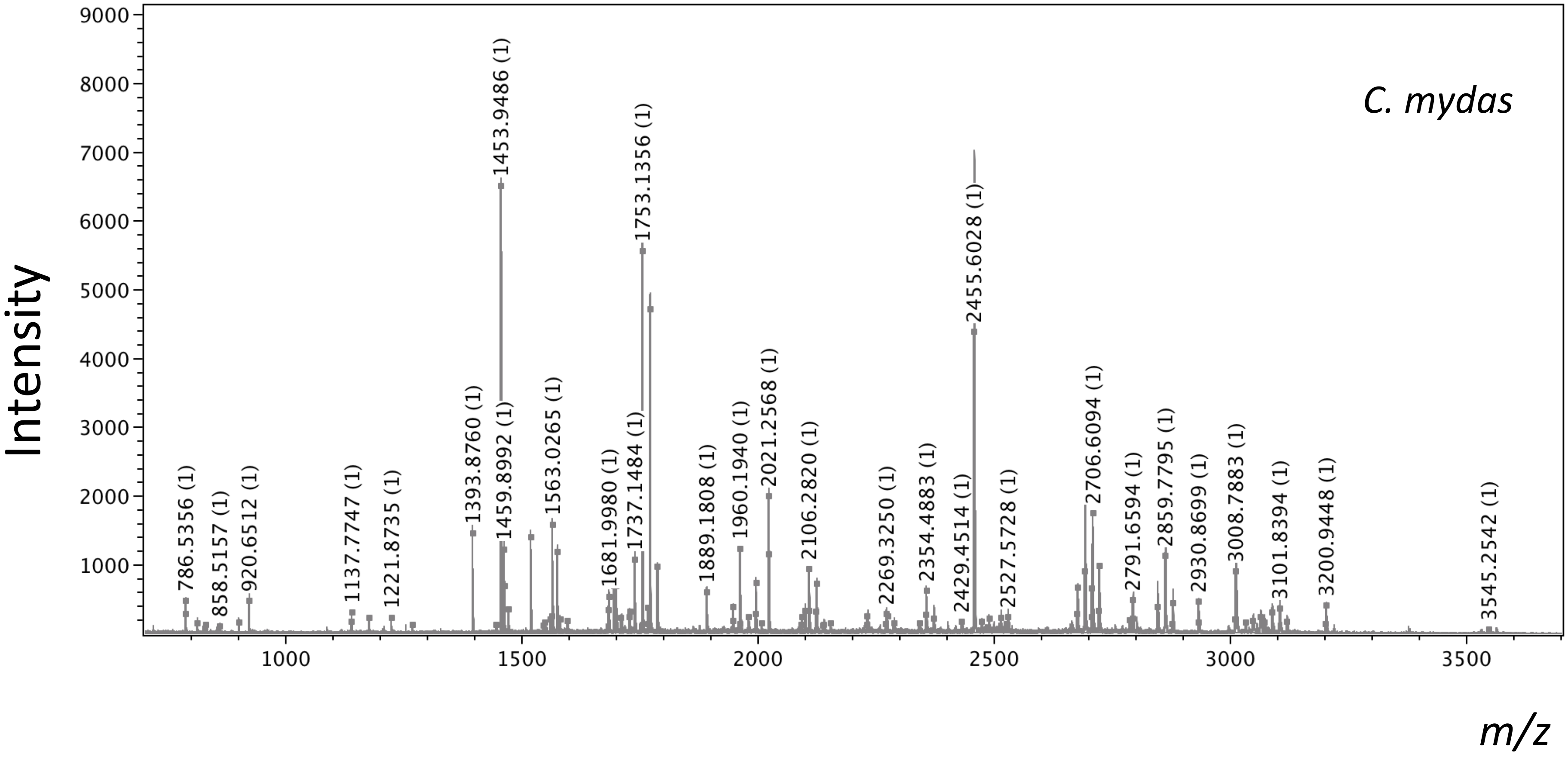

Supplementary Figure S1.14: Collagen fingerprint from a representative ancient sample of *Chelonia* spp. (Sample GP9) following digestion with trypsin.

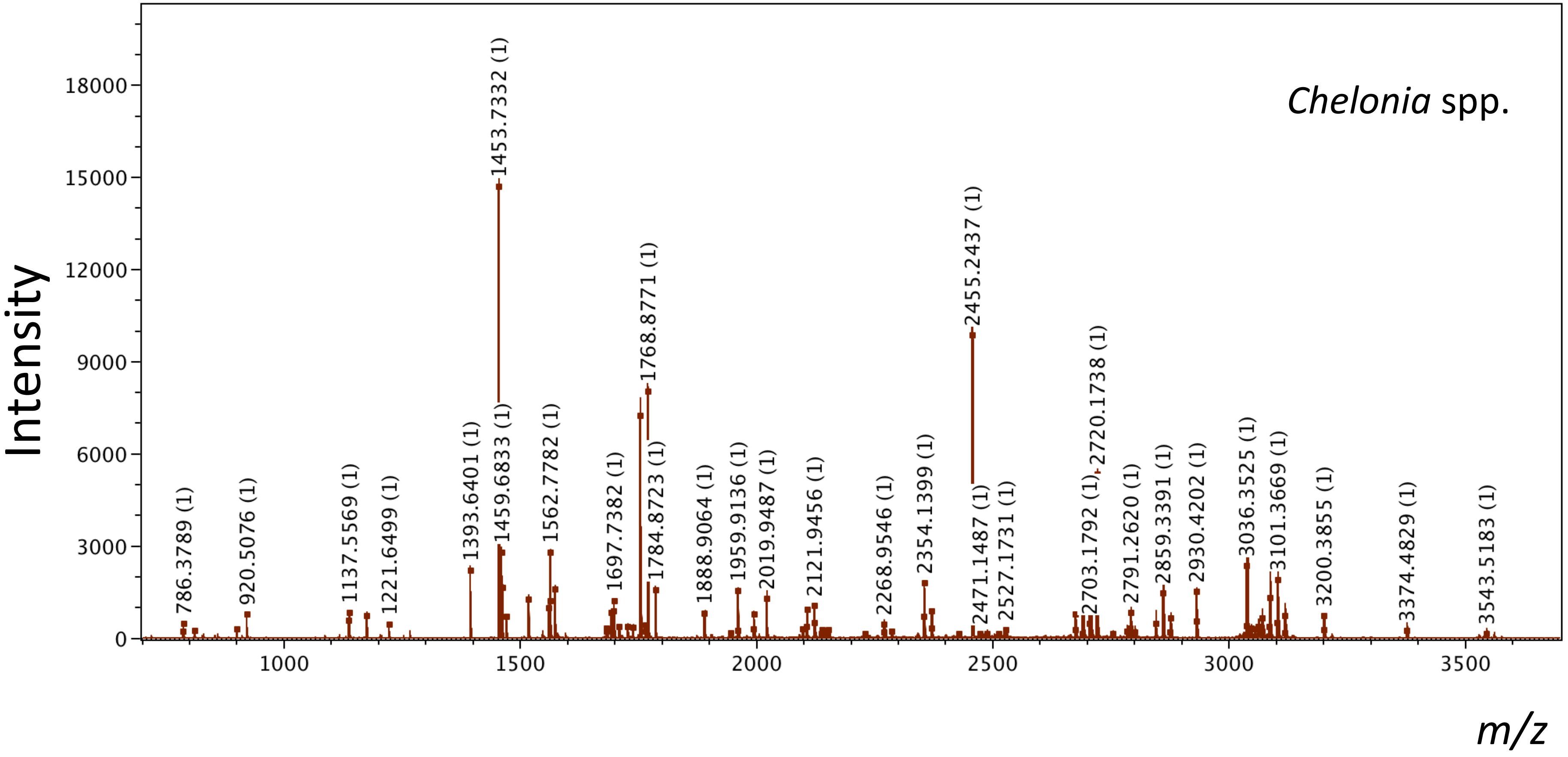

Supplementary Figure S1.15: Collagen fingerprint from a representative ancient sample of *Eretmochelys imbricata* (Sample GB71) following digestion with trypsin.

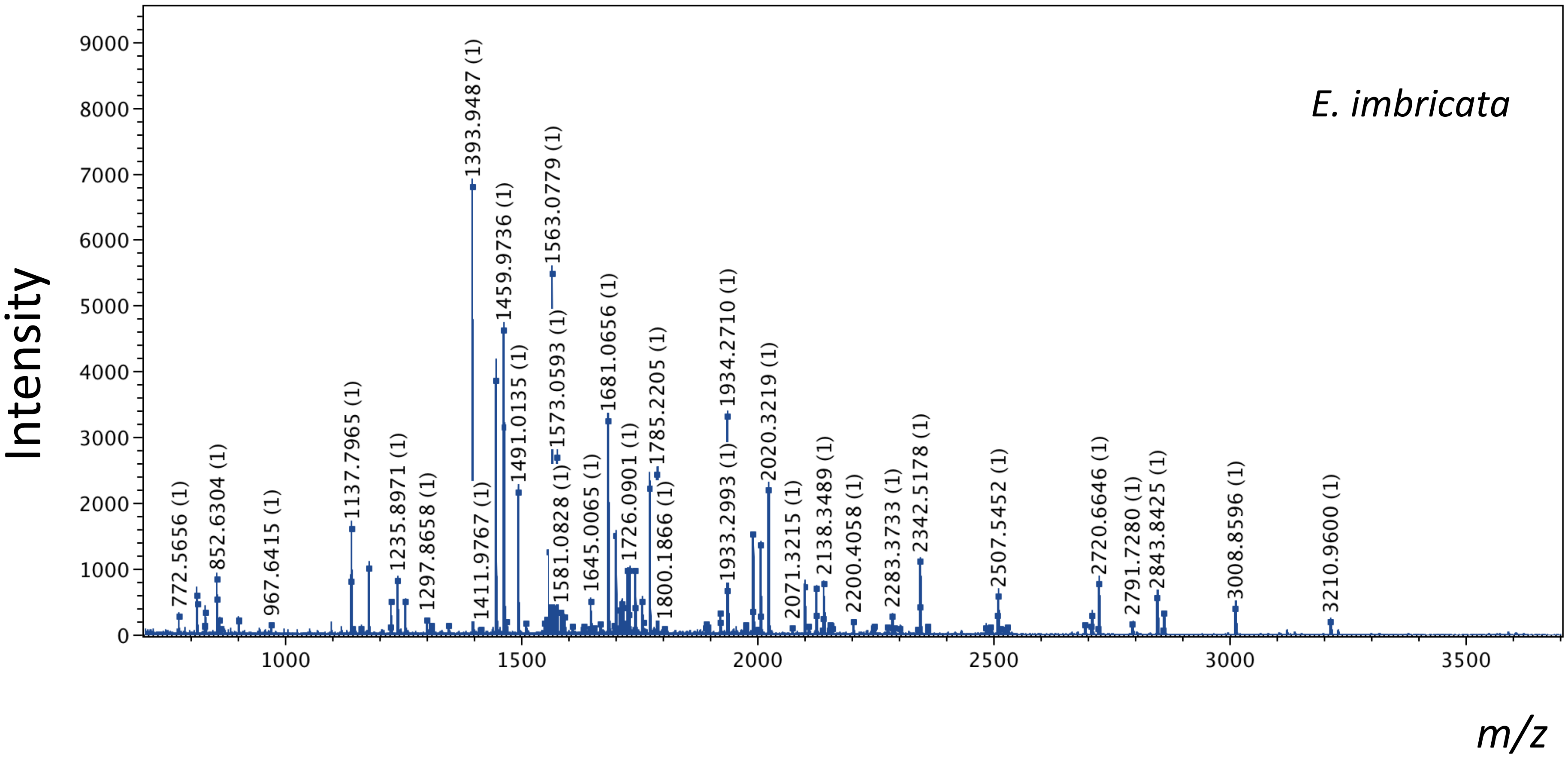

Supplementary Figure S1.16: Collagen fingerprint from a representative ancient sample of *Lepidochelys* spp. (Sample GP18) following digestion with trypsin.

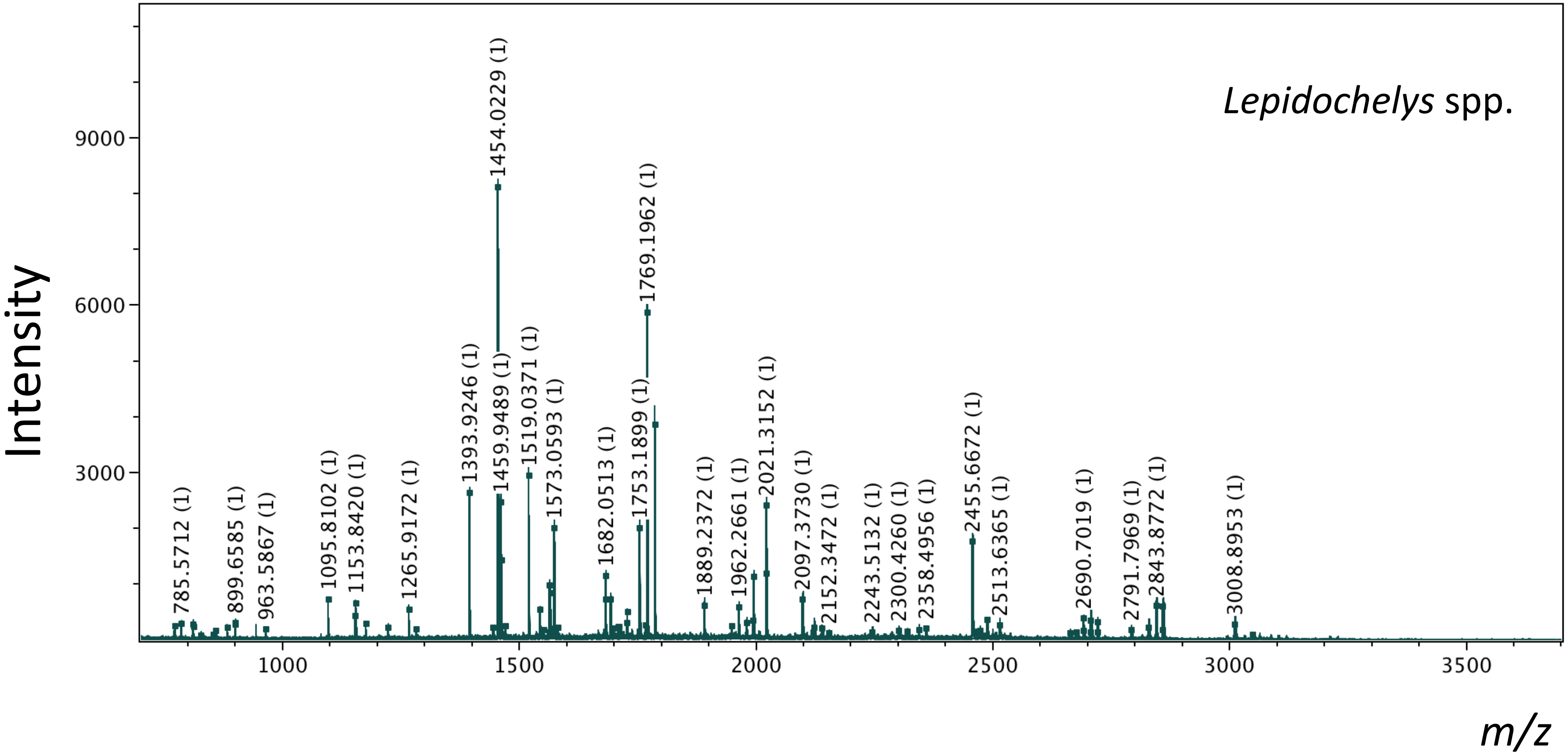

Supplementary Figure S1.17: Collagen fingerprint from a representative ancient sample of Chelydridae (Sample GP3) following digestion with trypsin.

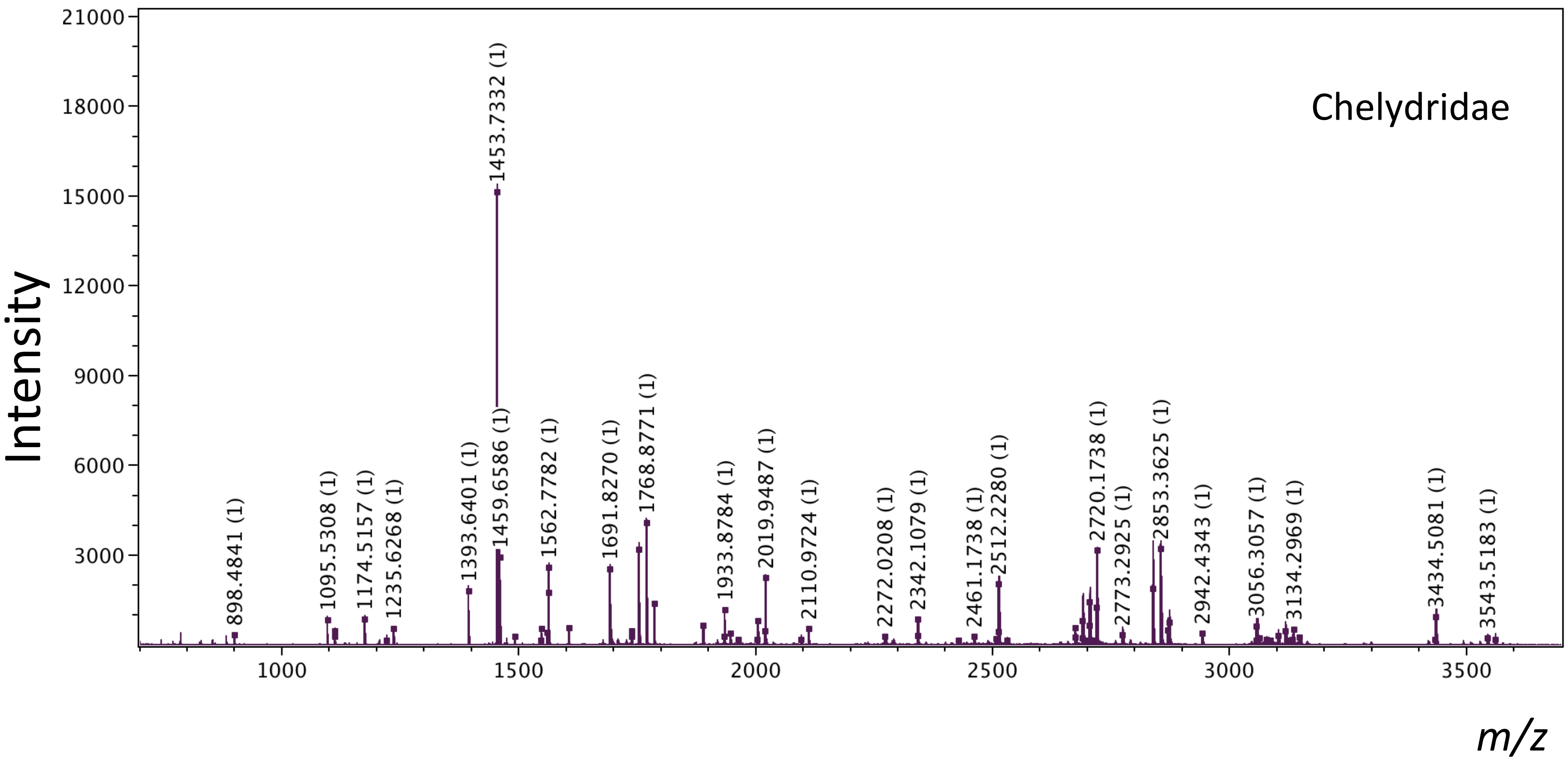

Supplementary Figure S1.18: Collagen fingerprint from a representative ancient sample of Emydidae (Sample GP26) following digestion with trypsin.

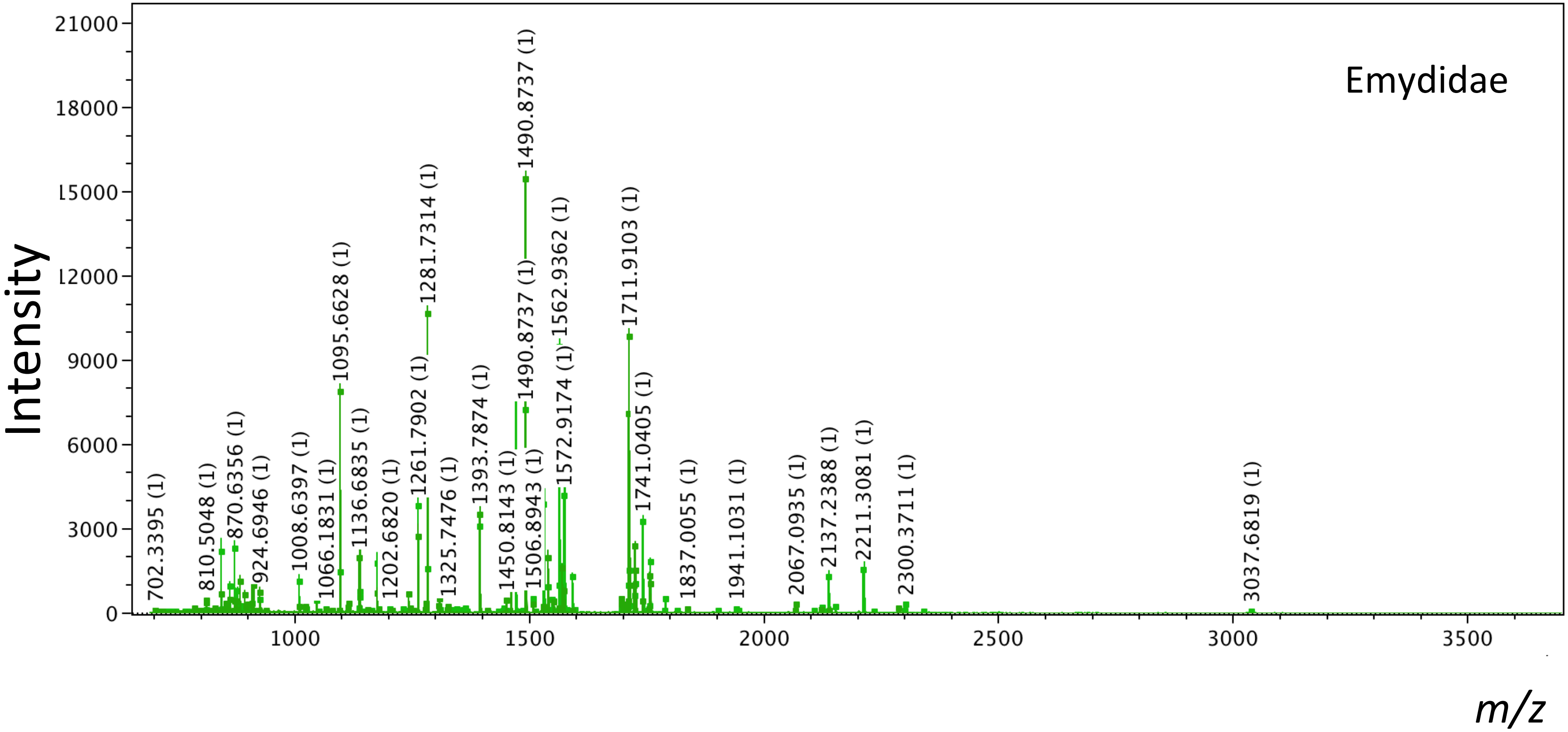

Supplementary Figure S1.19: Collagen fingerprint from a representative ancient sample of Testudinidae (Sample GP39) following digestion with trypsin.

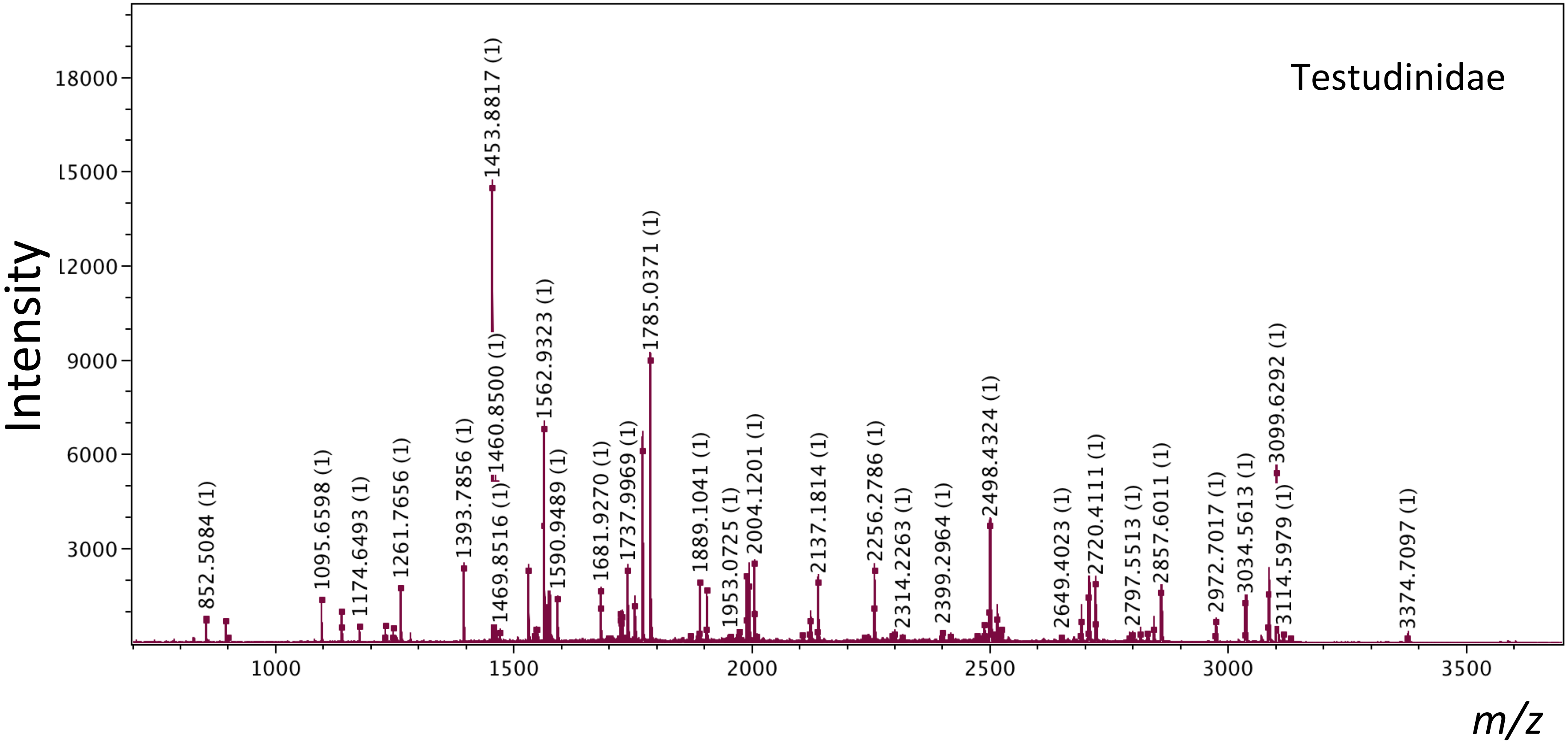

Supplementary Figure S2.1: Tandem mass spectrum for collagen (I) biomarker COL1A1T47 at *m/z* 1136 in *Chelonia mydas, Natator depressus, Caretta caretta, Eretmochelys imbricata* and *Dermochelys coriacea*.

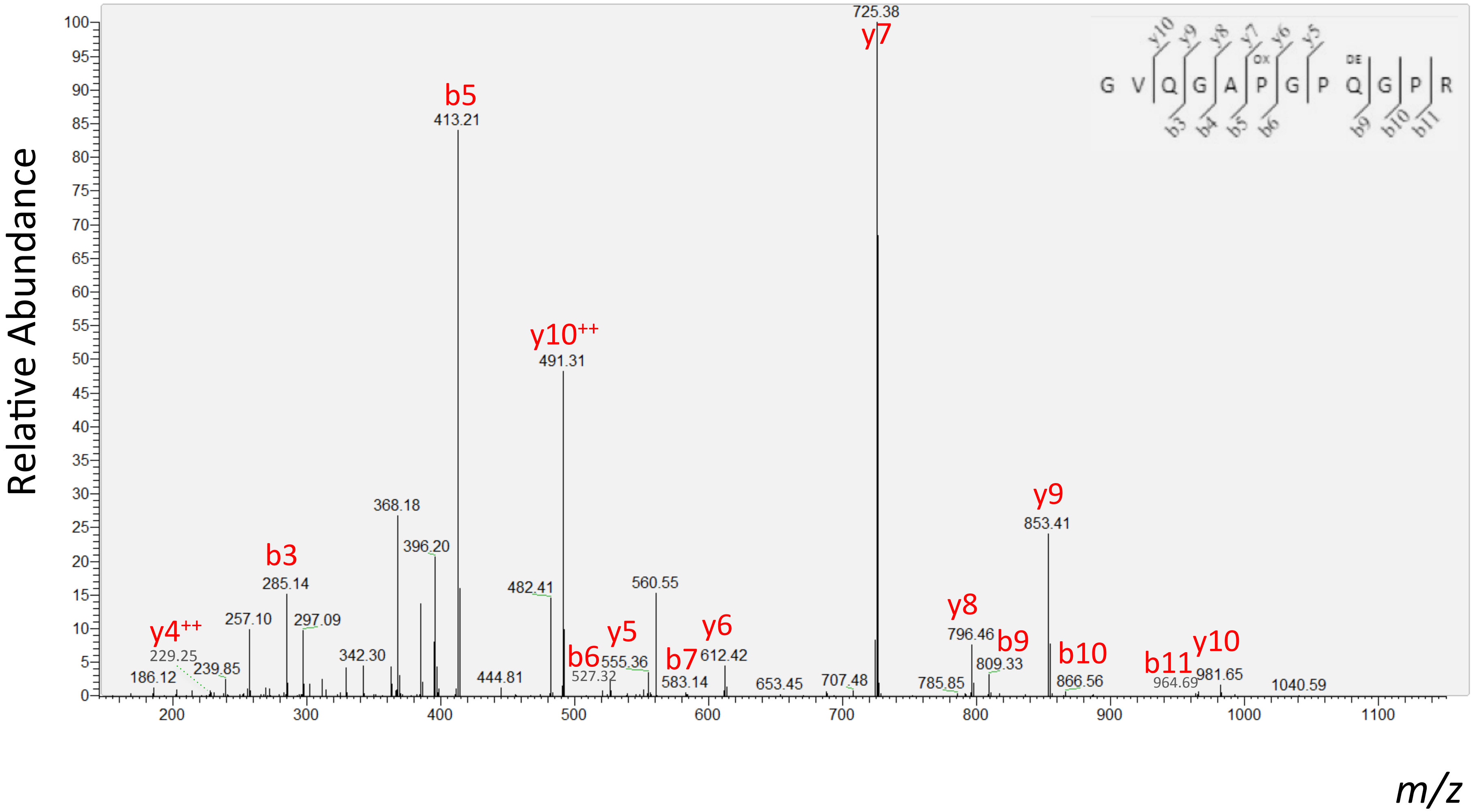

Supplementary Figure S2.2: Tandem mass spectrum for collagen (I) biomarker COL1A1T47 at *m/z* 1152 in *Lepidochelys kempii* and *L. olivacea*.

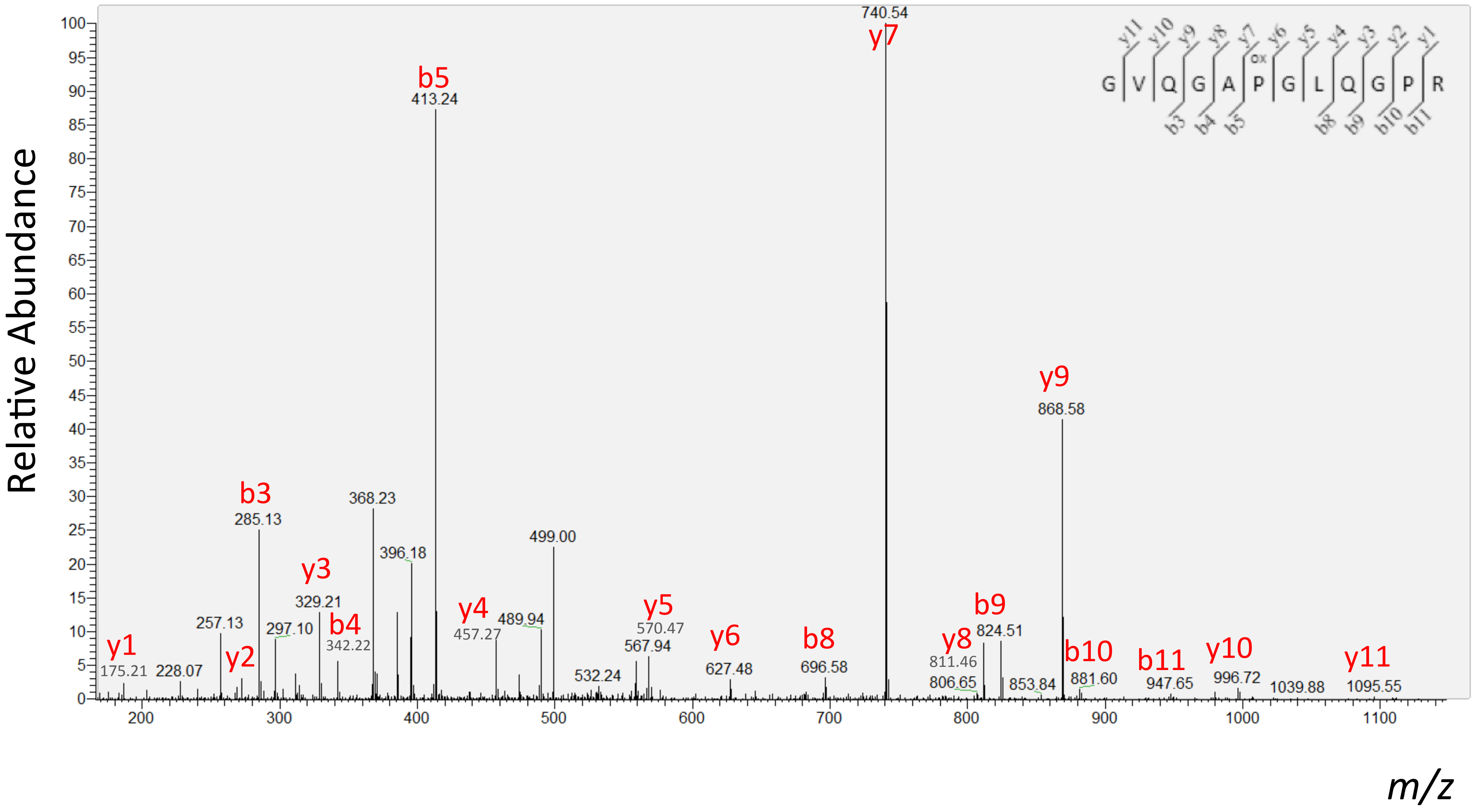

Supplementary Figure S2.3: Tandem mass spectrum for collagen (I) biomarker COL1A2T85 at *m/z* 1220 in all extant sea turtles.

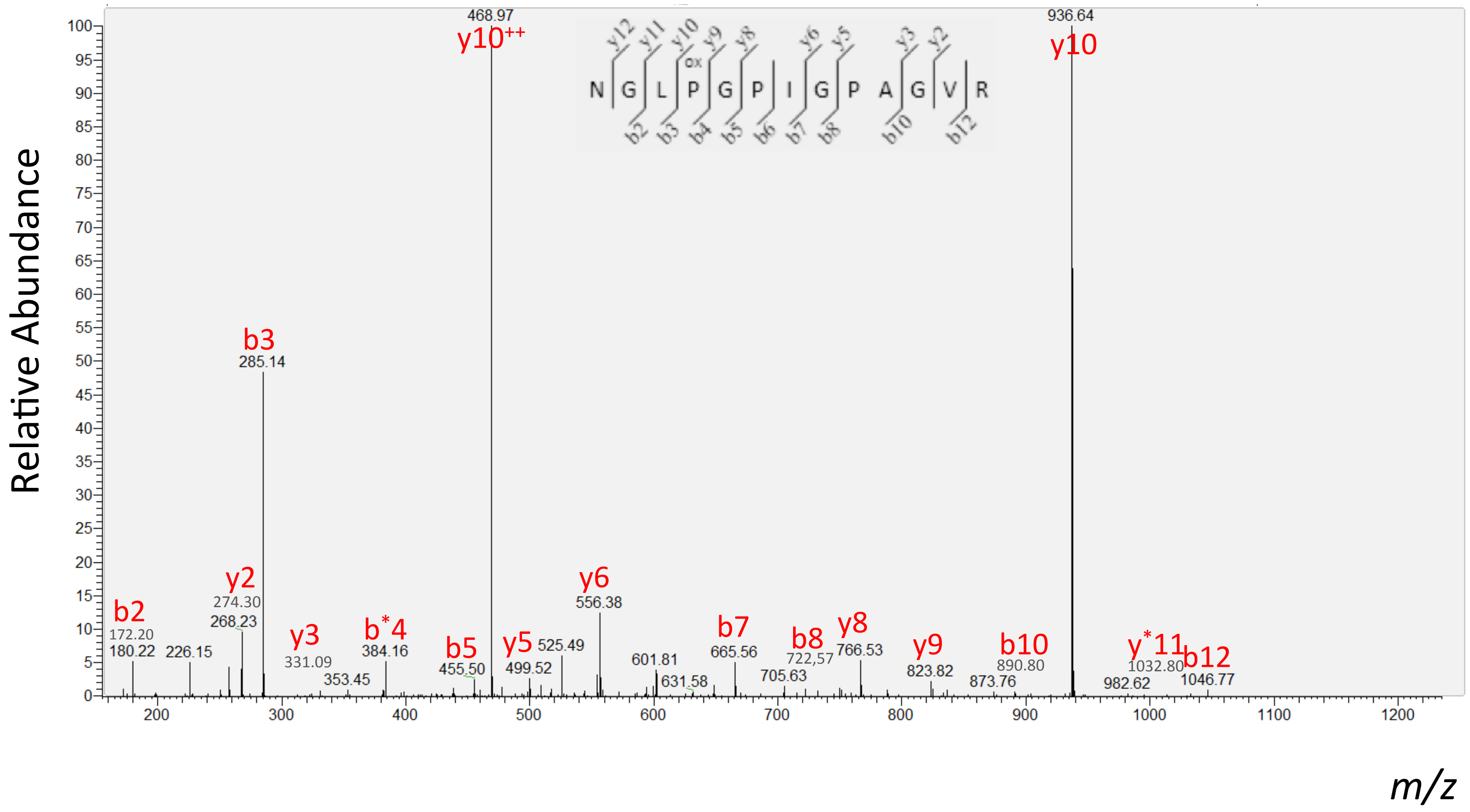

Supplementary Figure S2.4: Tandem mass spectrum for collagen (I) biomarker COL1A1T28 at *m/z* 1393 in all extant sea turtles.

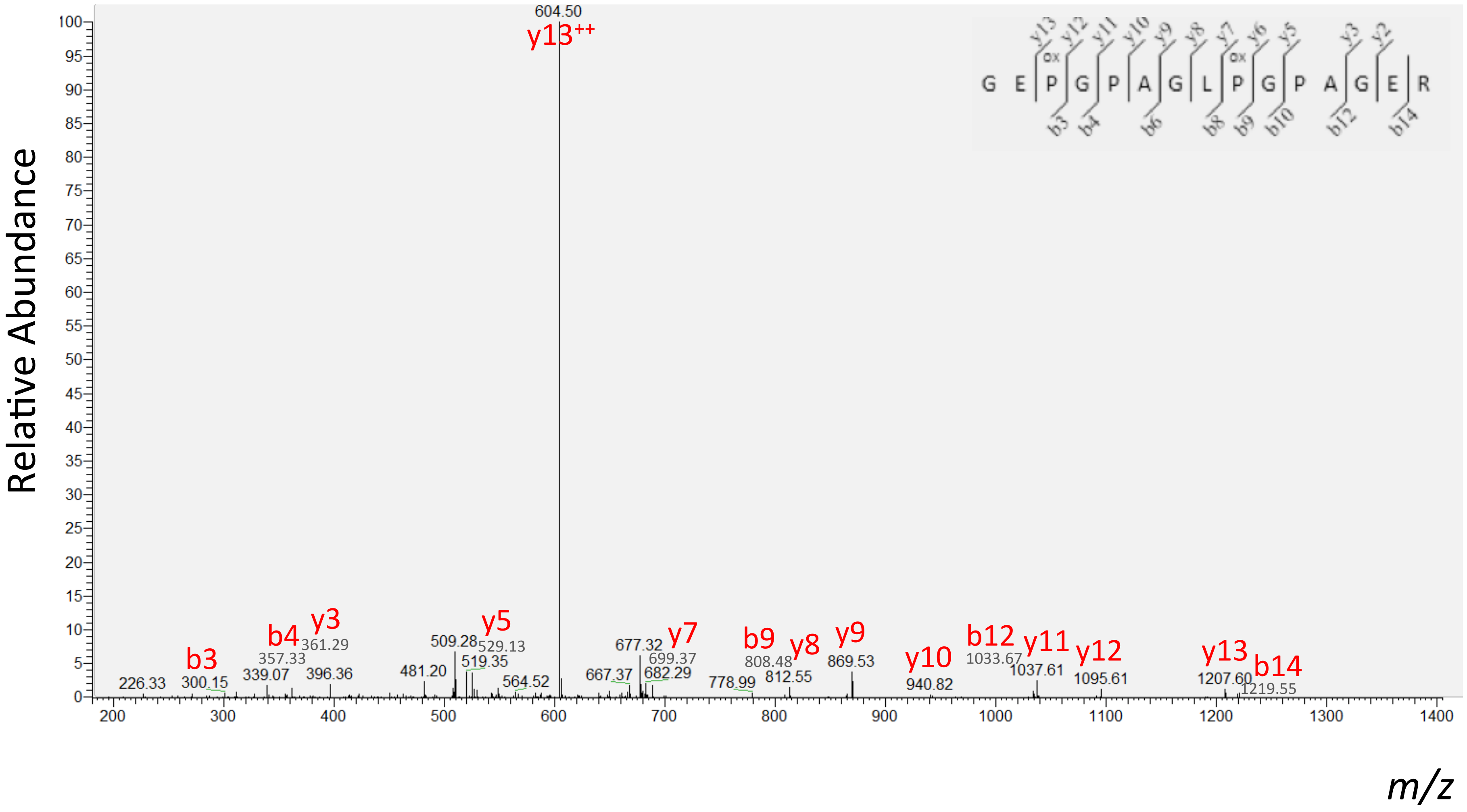

Supplementary Figure S2.5: Tandem mass spectrum for collagen (I) biomarker COL1A2T43 at *m/z* 1443 in *Eretmochelys imbricata*.

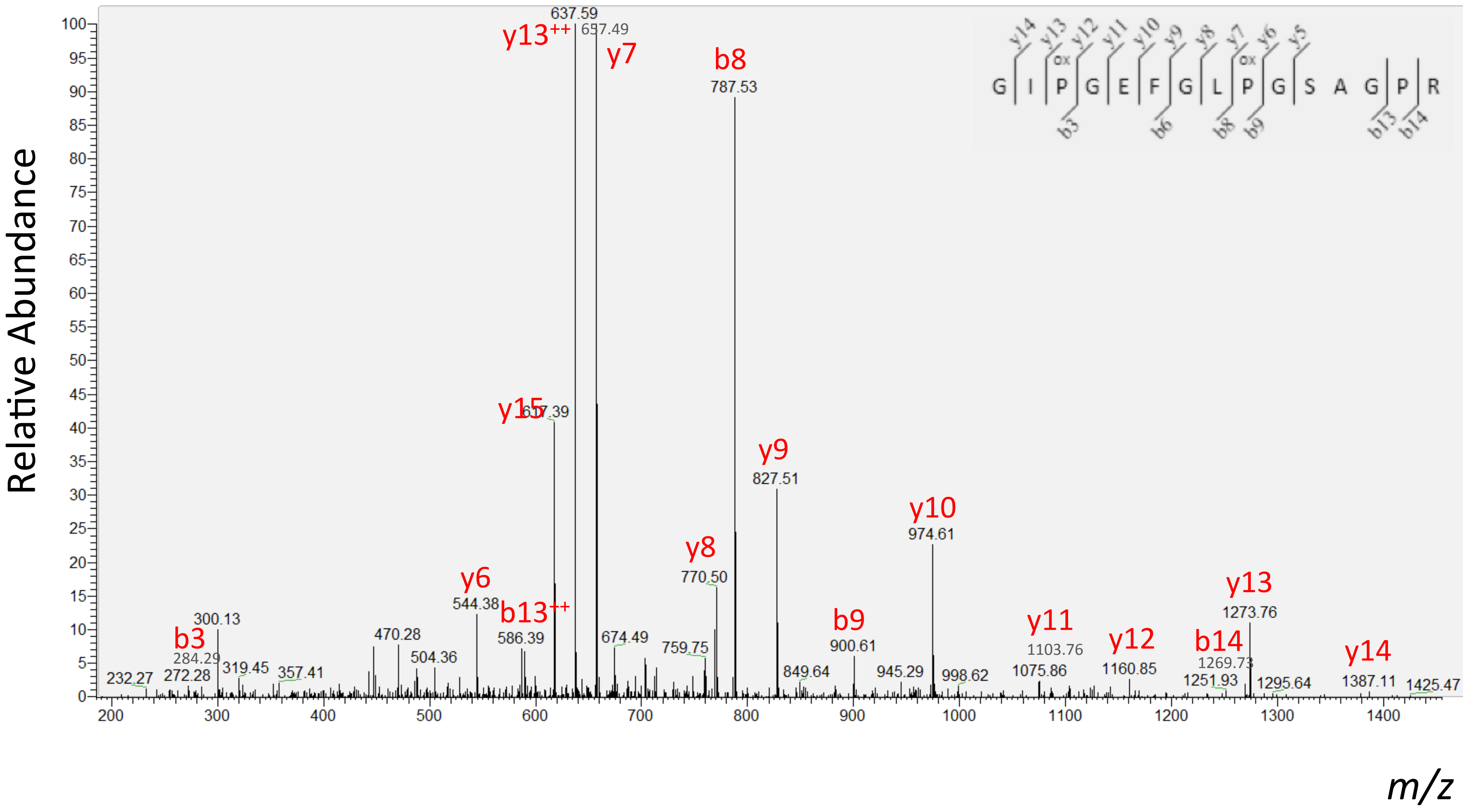

Supplementary Figure S2.6: Tandem mass spectrum for collagen (I) biomarker COL1A2T43 at *m/z* 1453 in *Chelonia mydas, Natator depressus, Caretta caretta, Lepidochelys kempii, L. olivacea* and *Dermochelys coriacea*.

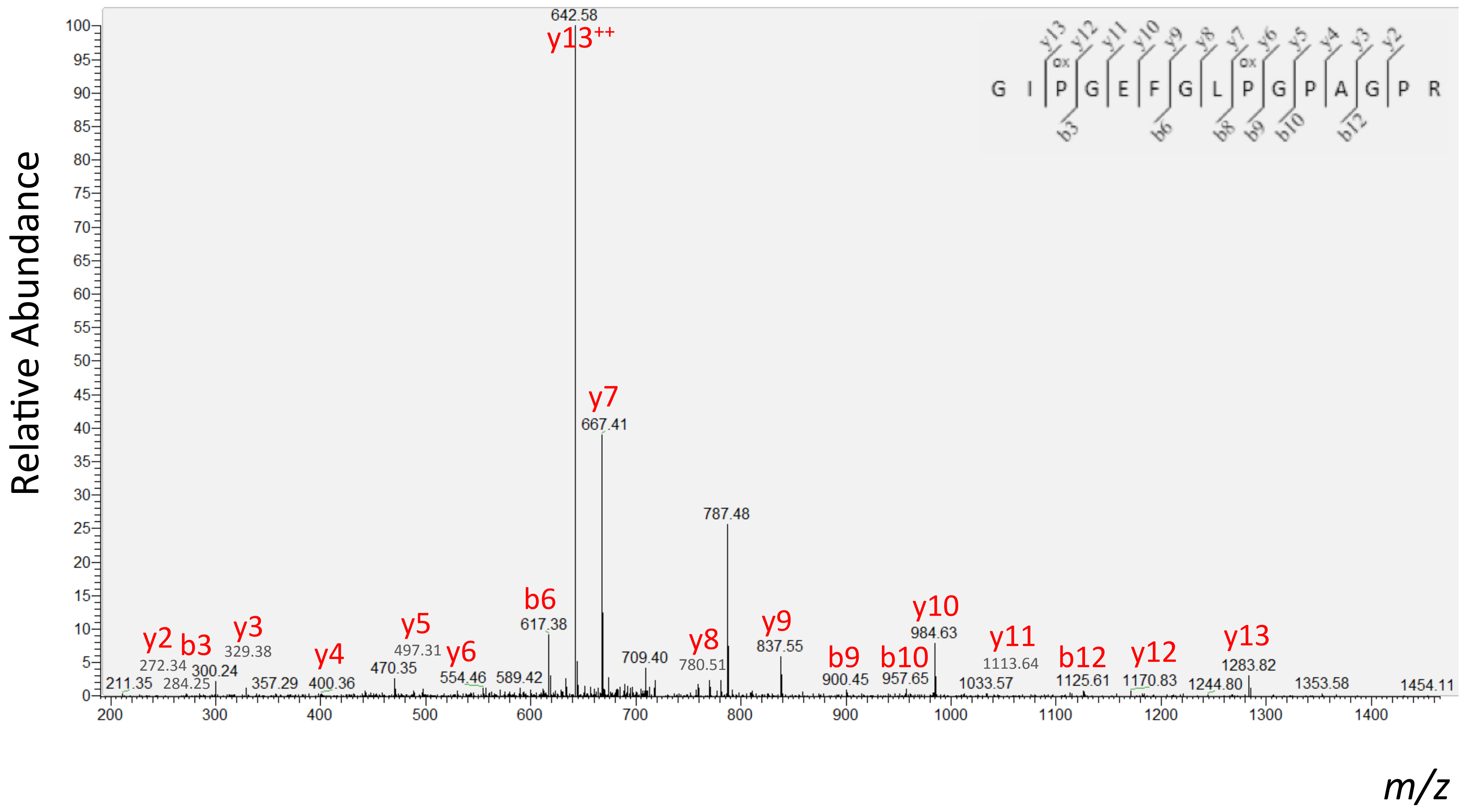

Supplementary Figure S2.7: Tandem mass spectrum for collagen (I) biomarker COL1A1T62 at *m/z* 1459 in all extant sea turtles.

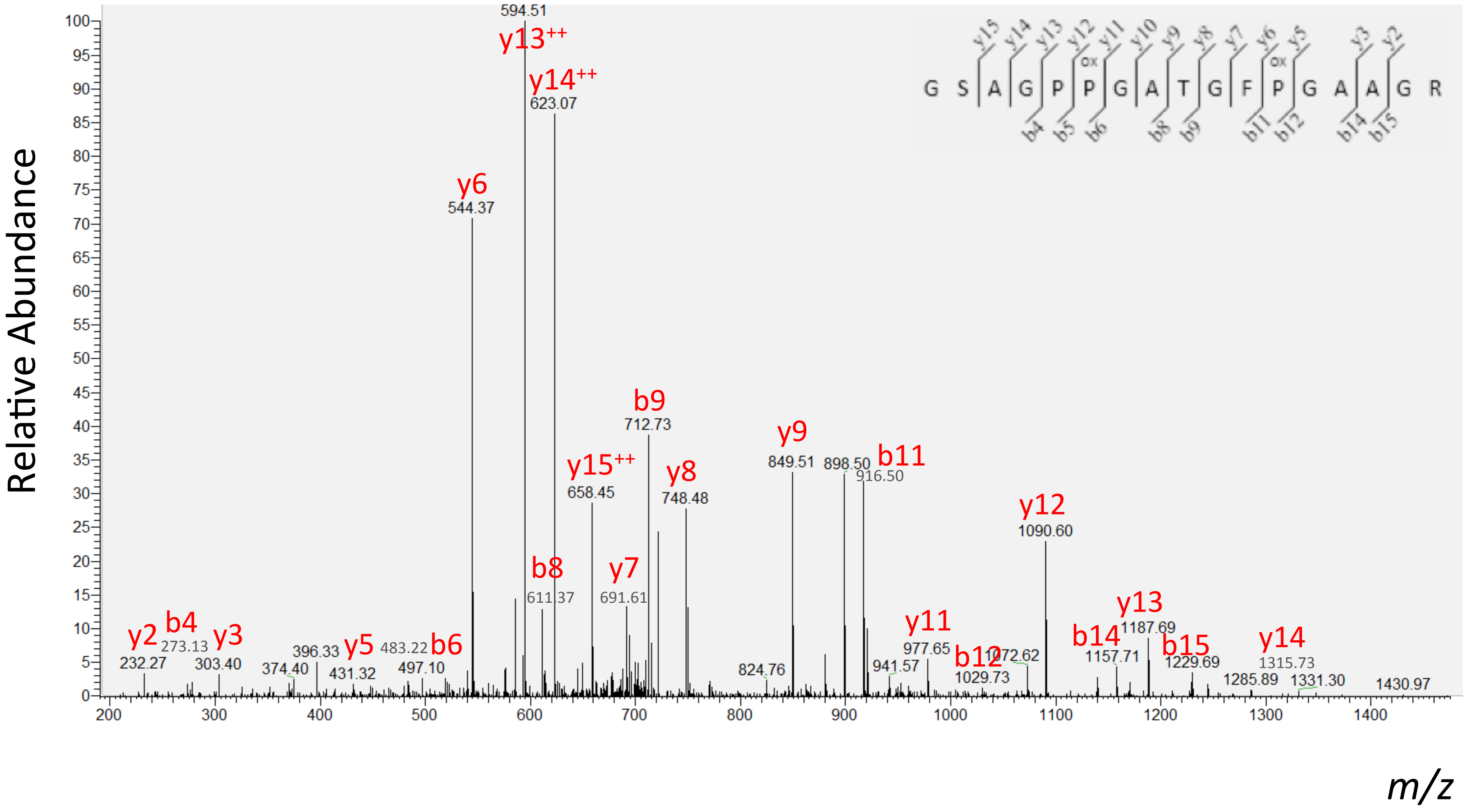

Supplementary Figure S2.8: Tandem mass spectrum for collagen (I) biomarker COL1A1T79 at *m/z* 1490 in *Natator depressus, Caretta caretta, Eretmochelys imbricata, Lepidochelys kempii, L. olivacea* and *Dermochelys coriacea*.

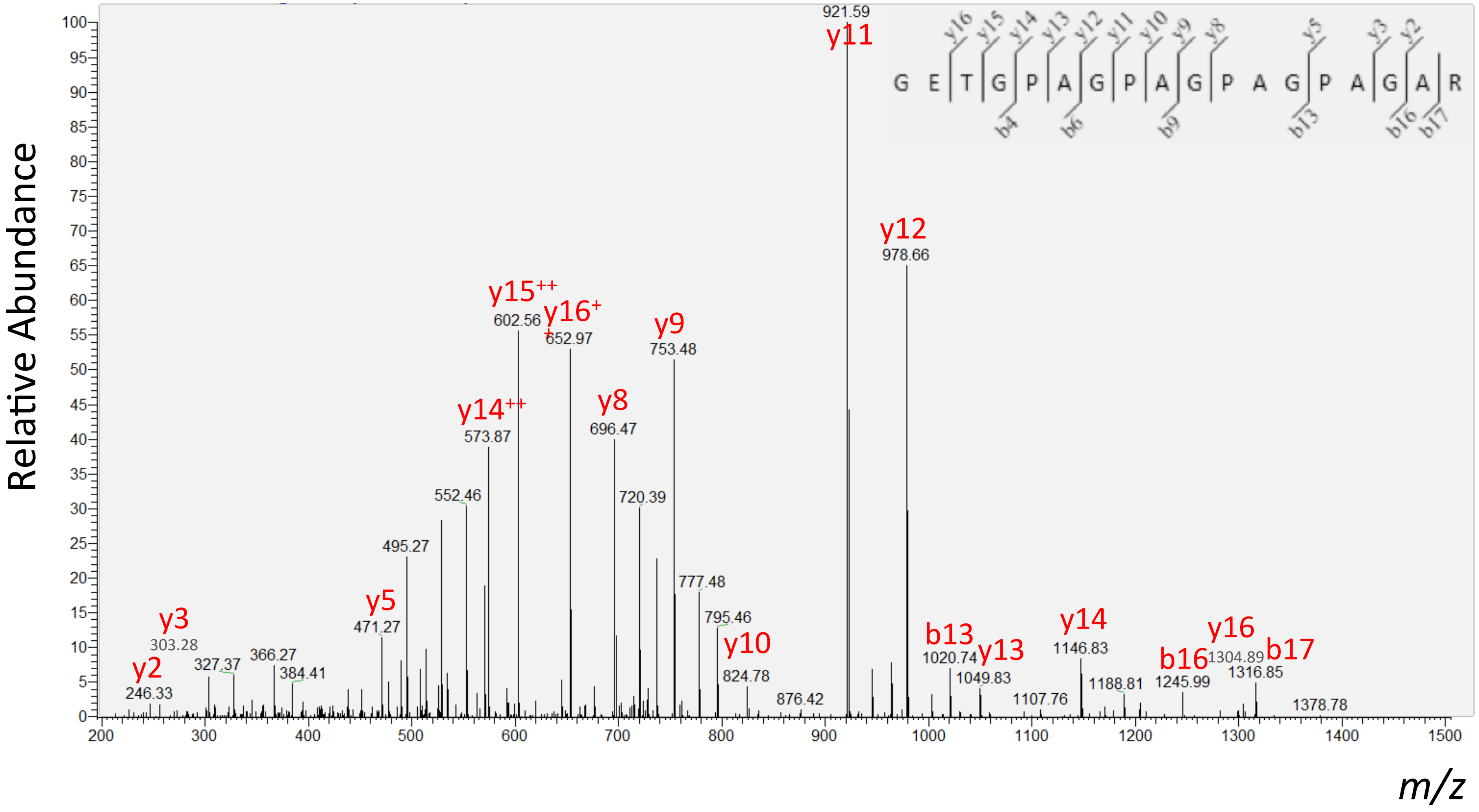

Supplementary Figure S2.9: Tandem mass spectrum for collagen (I) biomarker COL1A1T79 at *m/z* 1516 in *Chelonia mydas*.

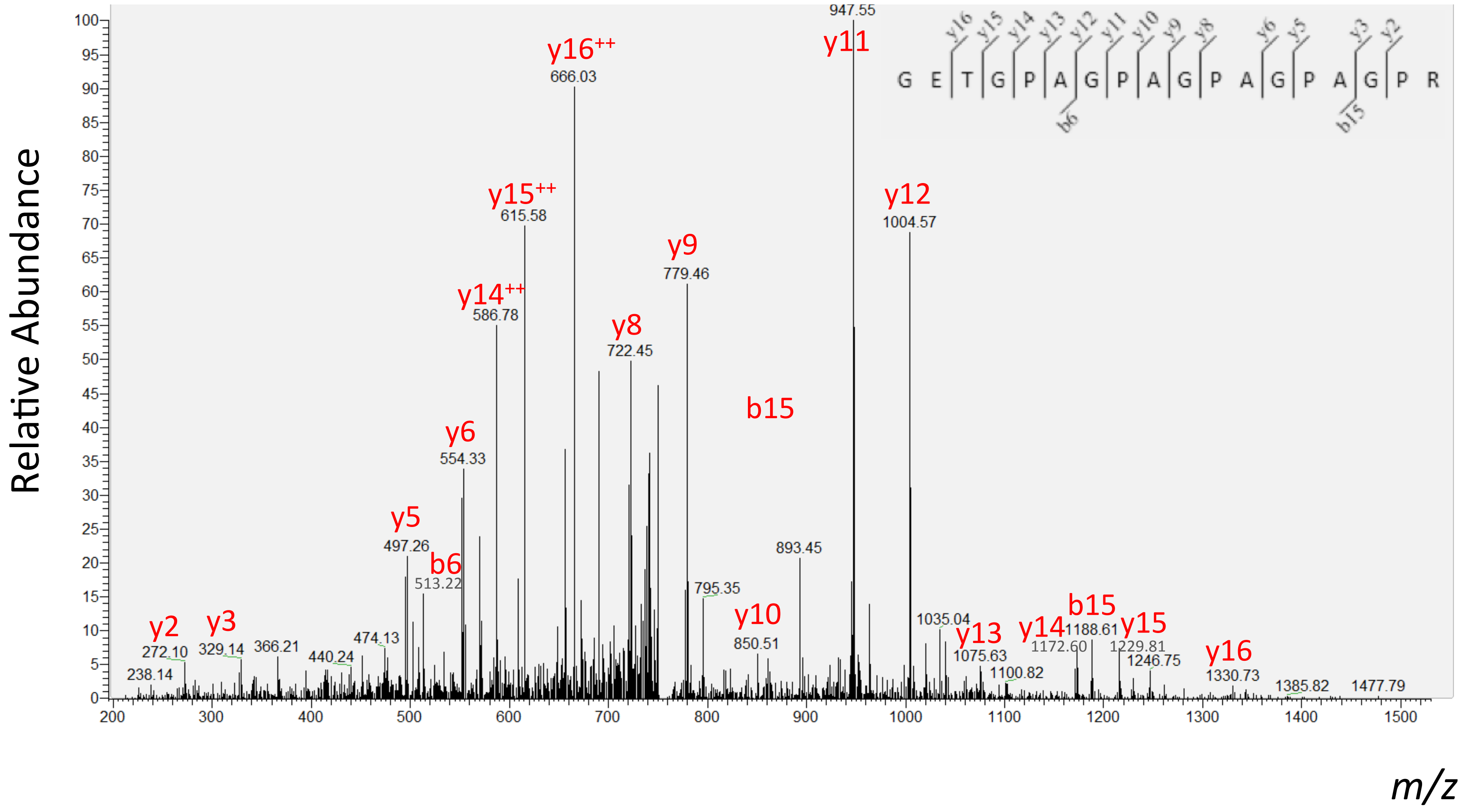

Supplementary Figure S2.10: Tandem mass spectrum for collagen (I) biomarker COL1A1T21 at *m/z* 1572 in all extant sea turtles.

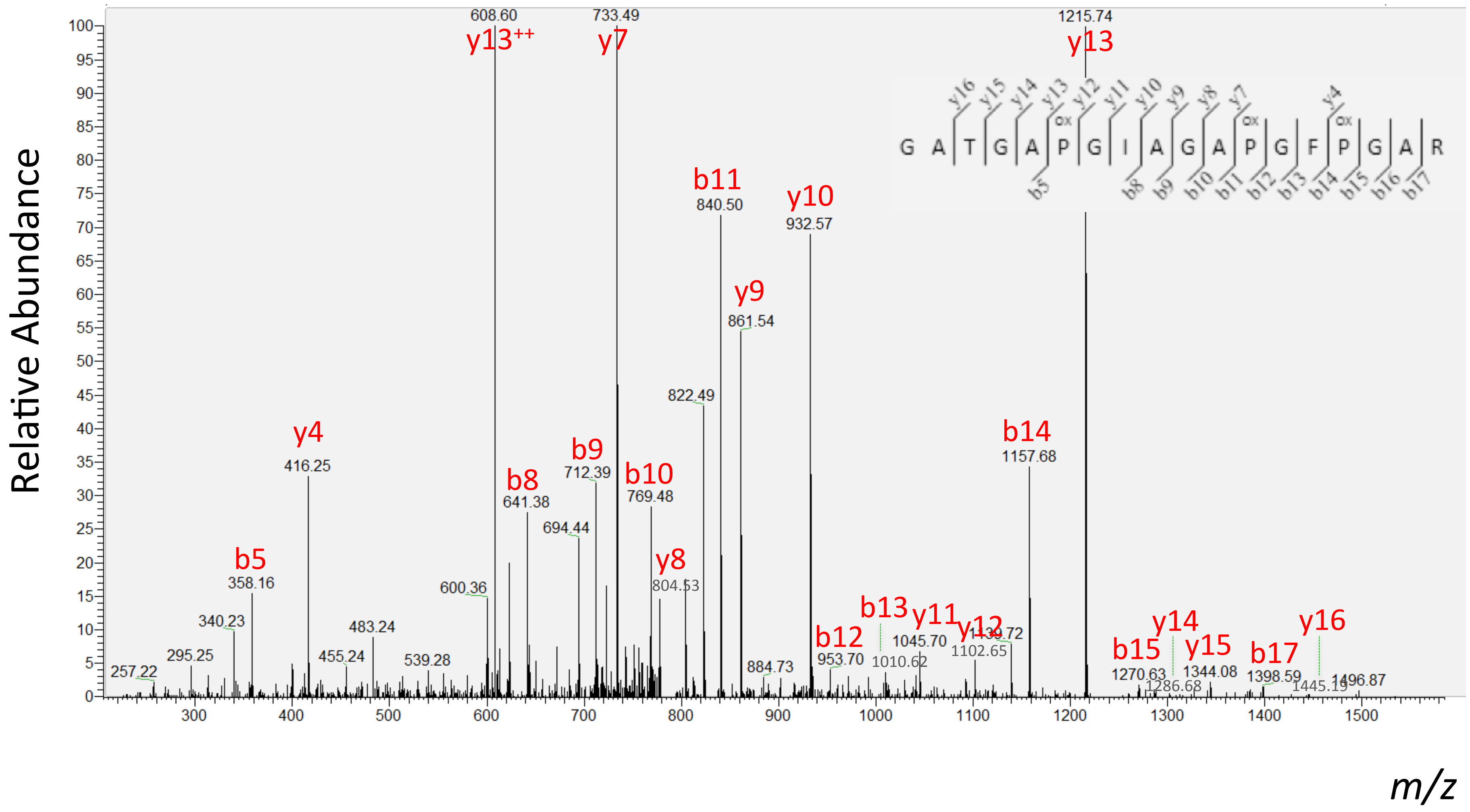

Supplementary Figure S2.11: Tandem mass spectrum for collagen (I) biomarker COL1A2T69 at *m/z* 2097 in *Chelonia mydas, Caretta caretta, Eretmochelys imbricata, Lepidochelys kempii* and *L. olivacea.*

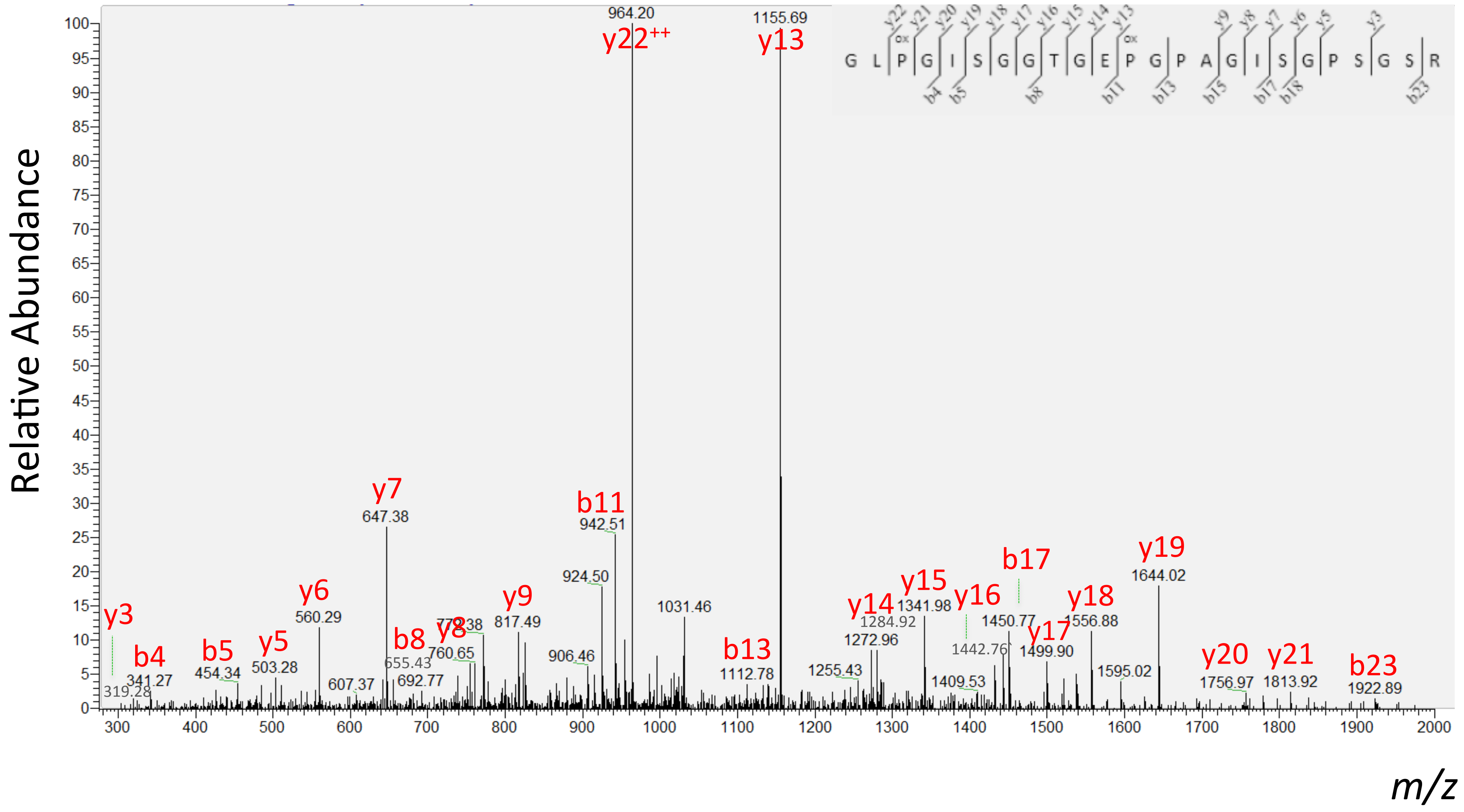

Supplementary Figure S2.12: Tandem mass spectrum for collagen (I) biomarker COL1A2T69 at *m/z* 2107 in *Natator depressus*.

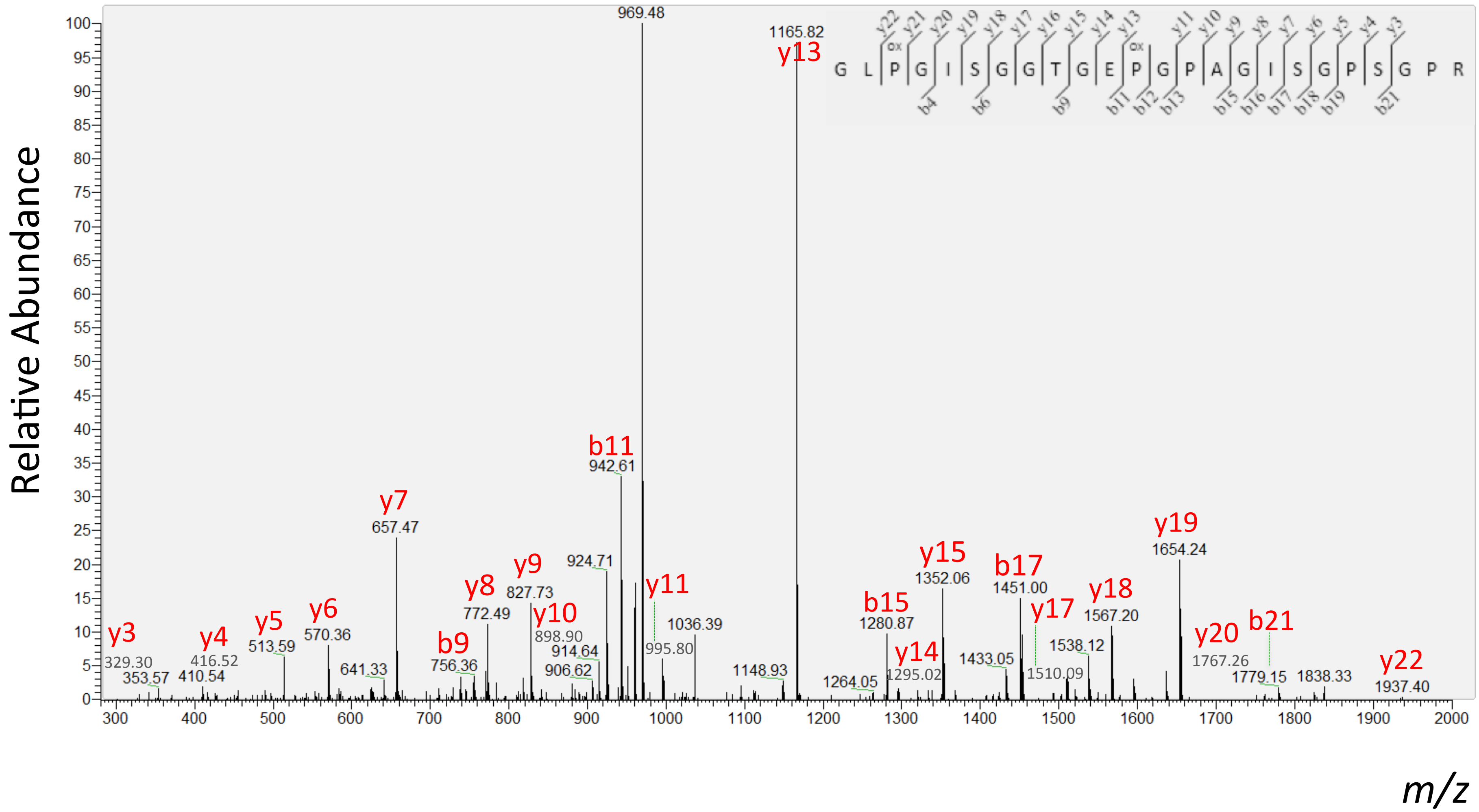

Supplementary Figure S2.13: Tandem mass spectrum for collagen (I) biomarker COL1A2T69 at *m/z* 2125 in *Dermochelys coriacea*.

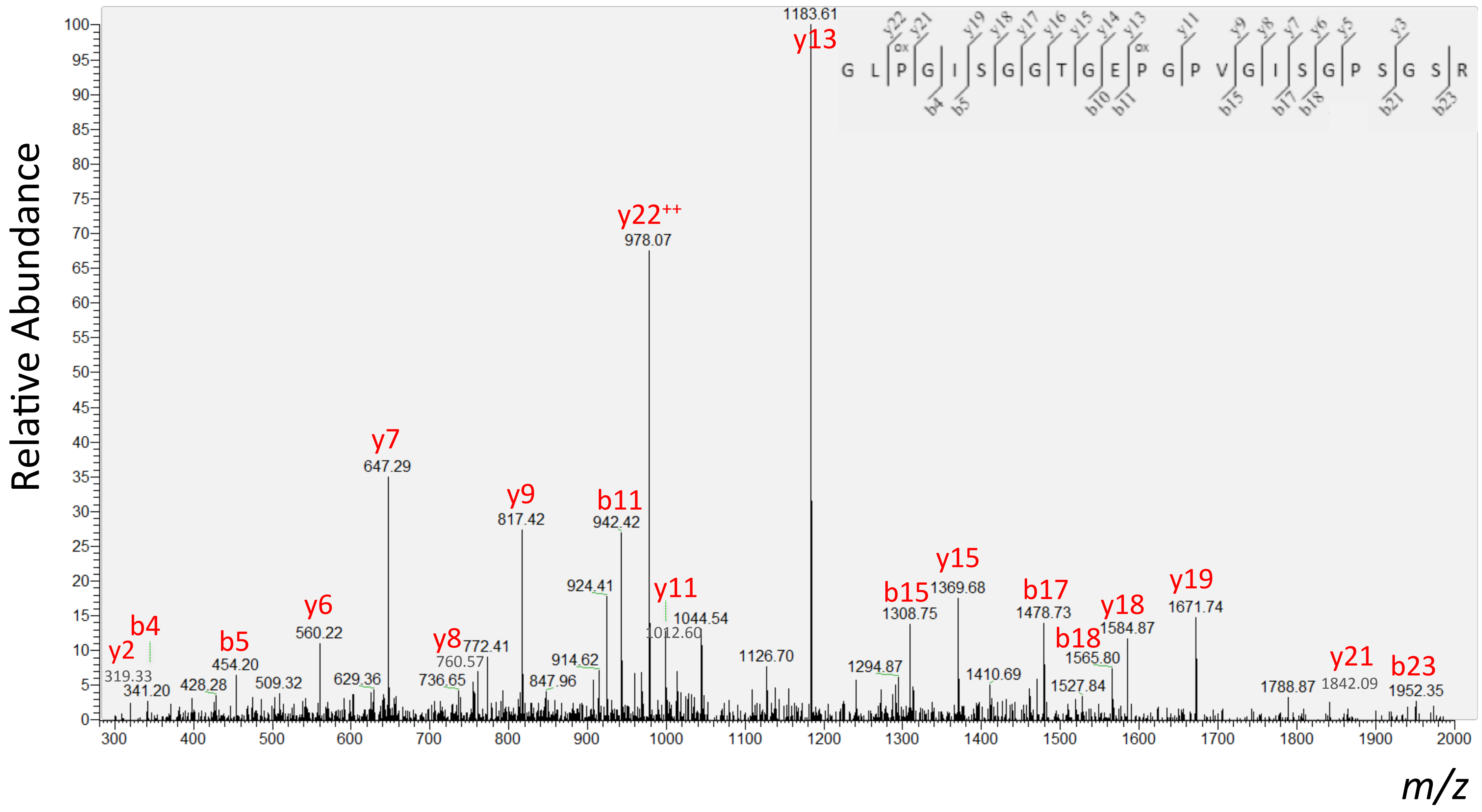

Supplementary Figure S2.14: Tandem mass spectrum for collagen (I) biomarker COL1A2T65/66 at *m/z* 2311 in *Dermochelys coriacea*.

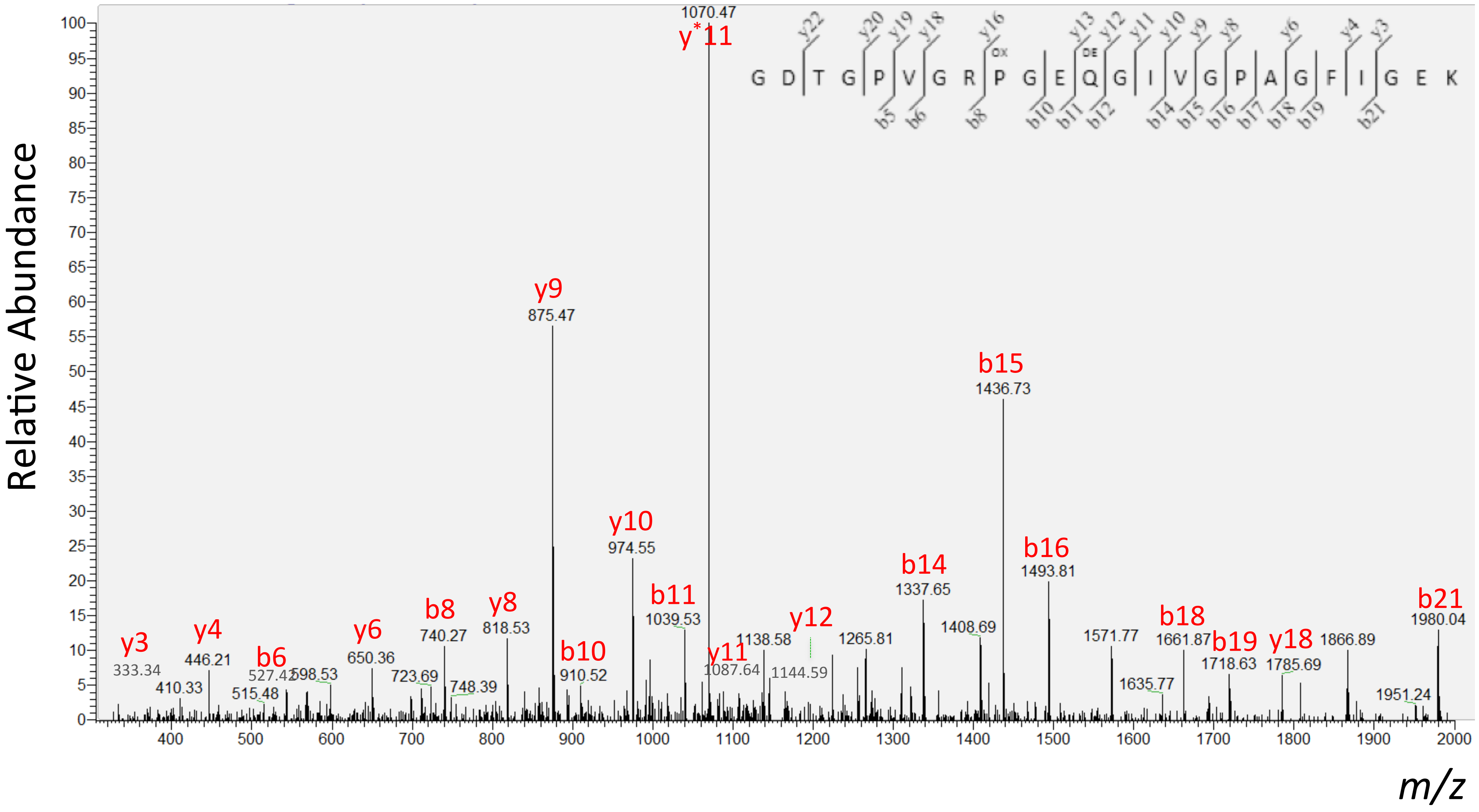

Supplementary Figure S2.15: Tandem mass spectrum for collagen (I) biomarker COL1A2T65/66 at *m/z* 2341 in *Natator depressus, Caretta caretta, Eretmochelys imbricata* and *Lepidochelys kempii* and *L. olivacea*.

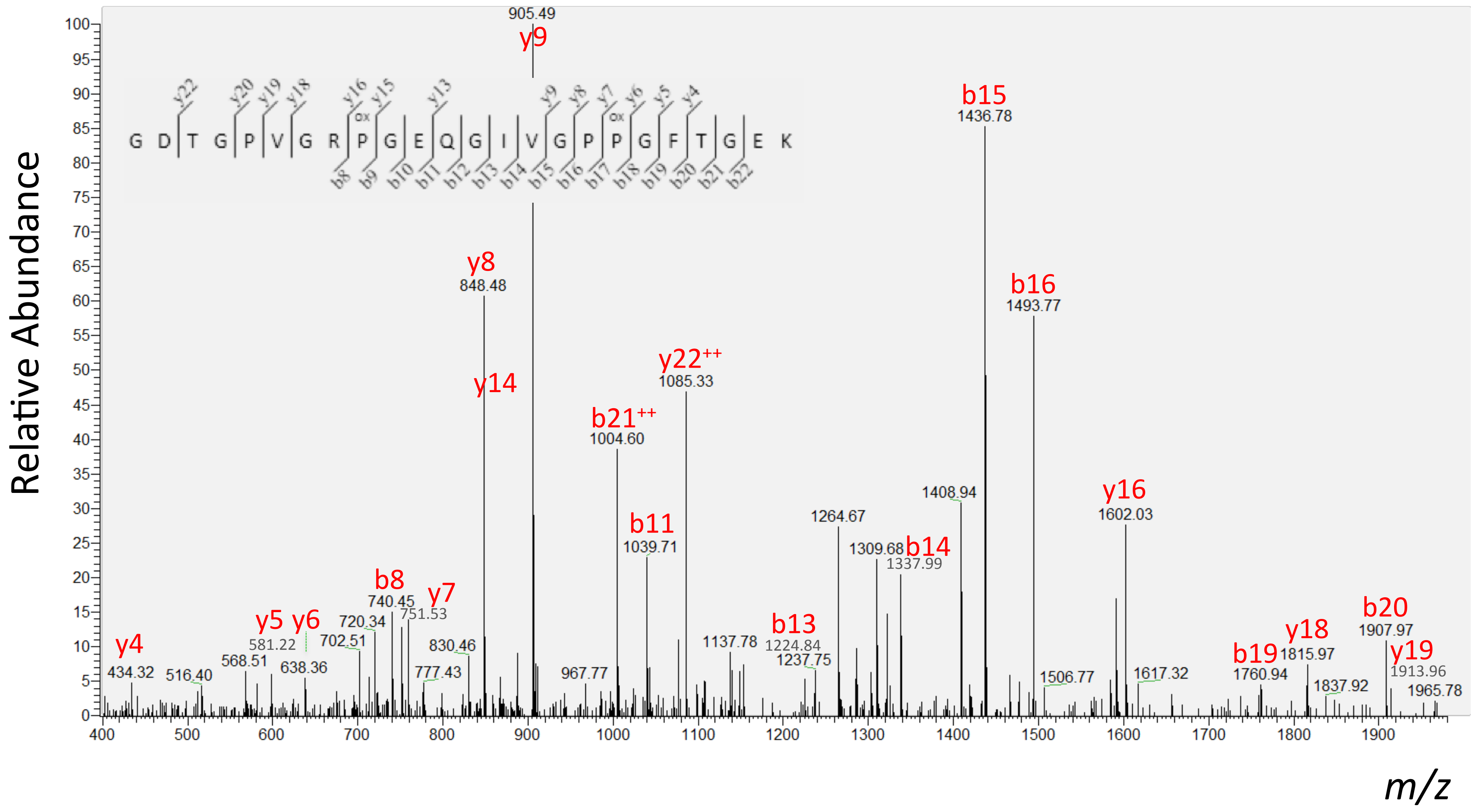

Supplementary Figure S2.16: Tandem mass spectrum for collagen (I) biomarker COL1A2T65/66 at *m/z* 2353 in *Chelonia mydas*.

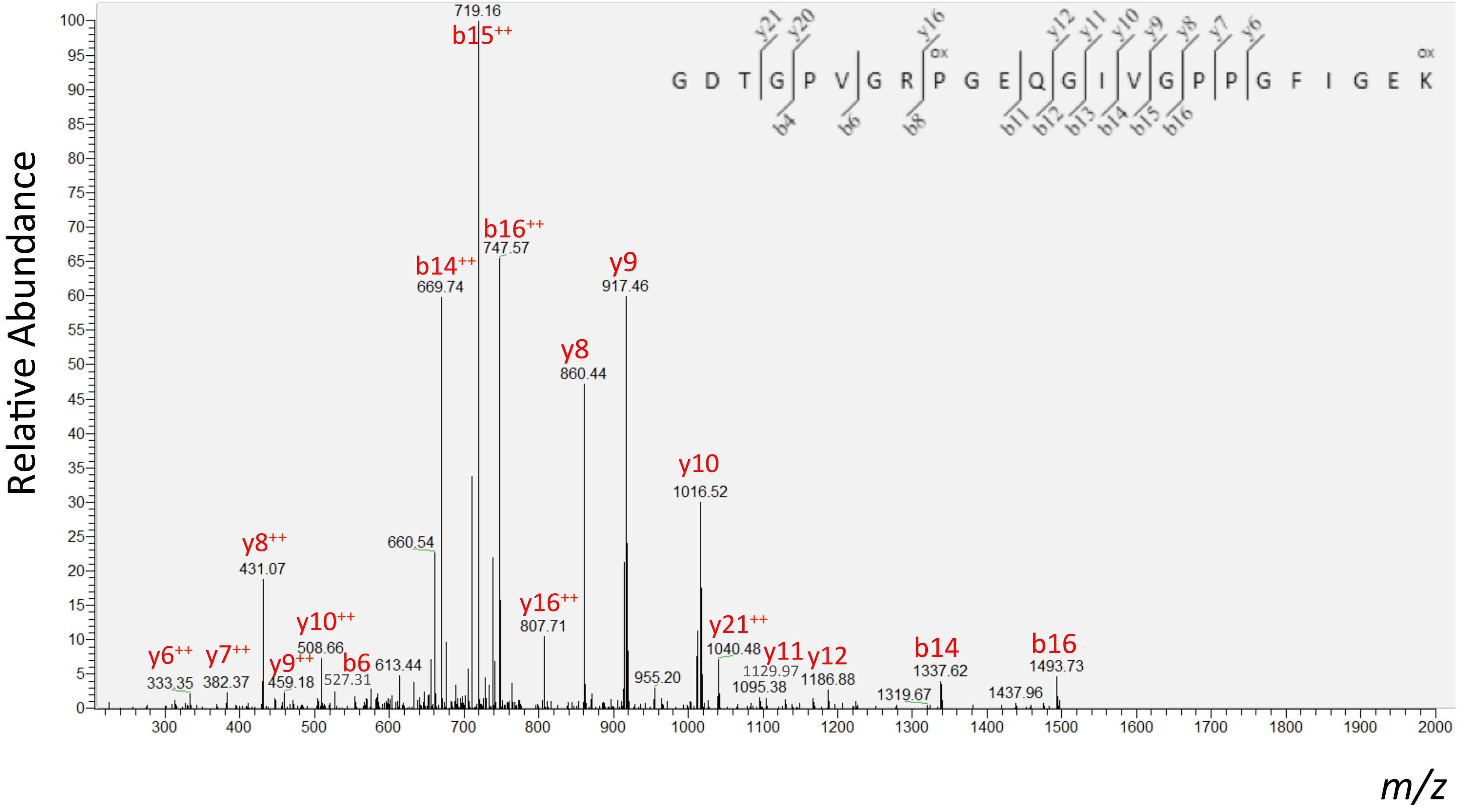

Supplementary Figure S2.17: Tandem mass spectrum for collagen (I) biomarker COL1A2T60 at *m/z* 2455 in *Chelonia mydas, Natator depressus, Caretta caretta, Lepidochelys kempii* and *L. olivacea*.

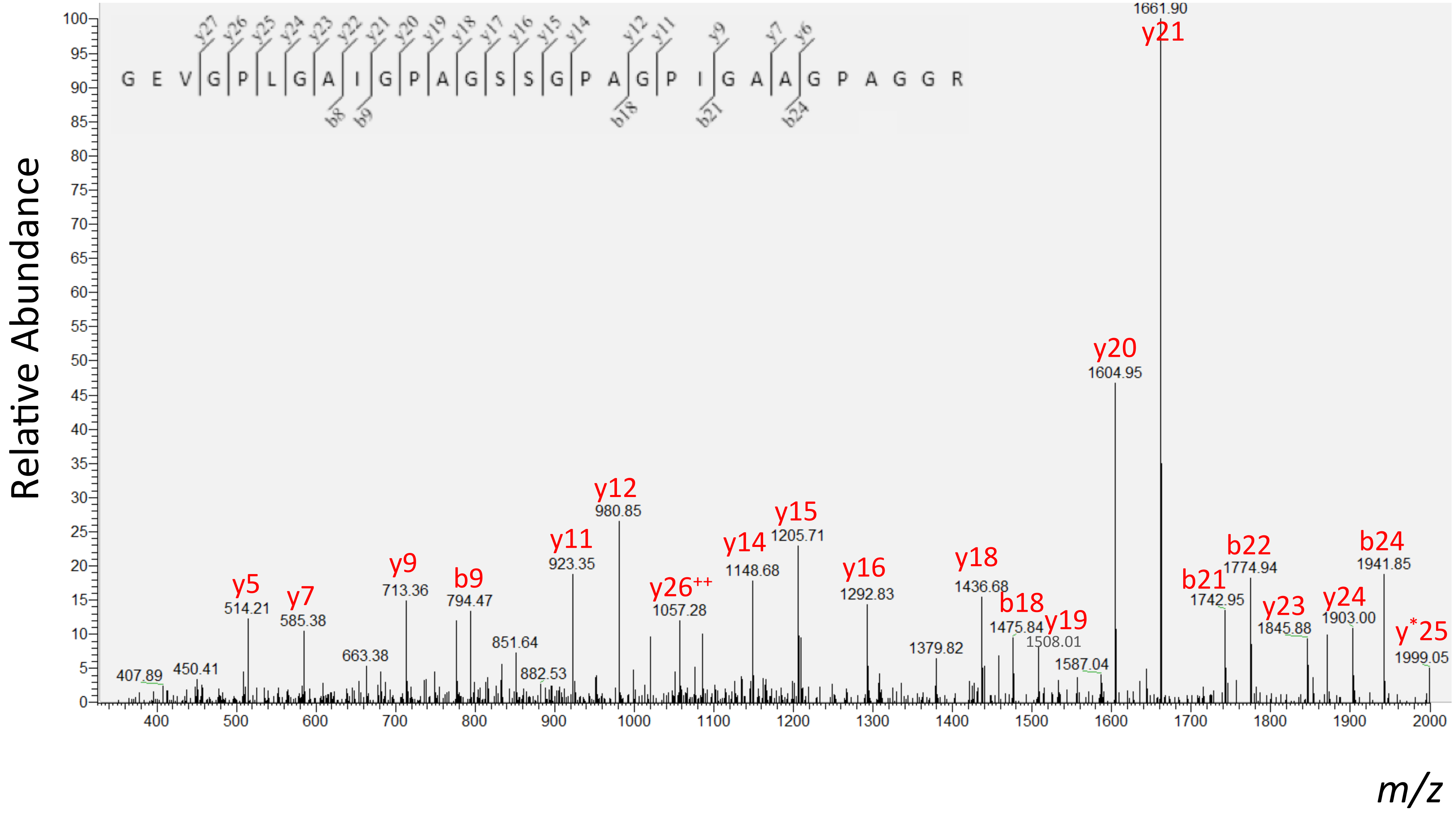

Supplementary Figure S2.18: Tandem mass spectrum for collagen (I) biomarker COL1A2T60 at *m/z* 2485 in *Eretmochelys imbricata*.

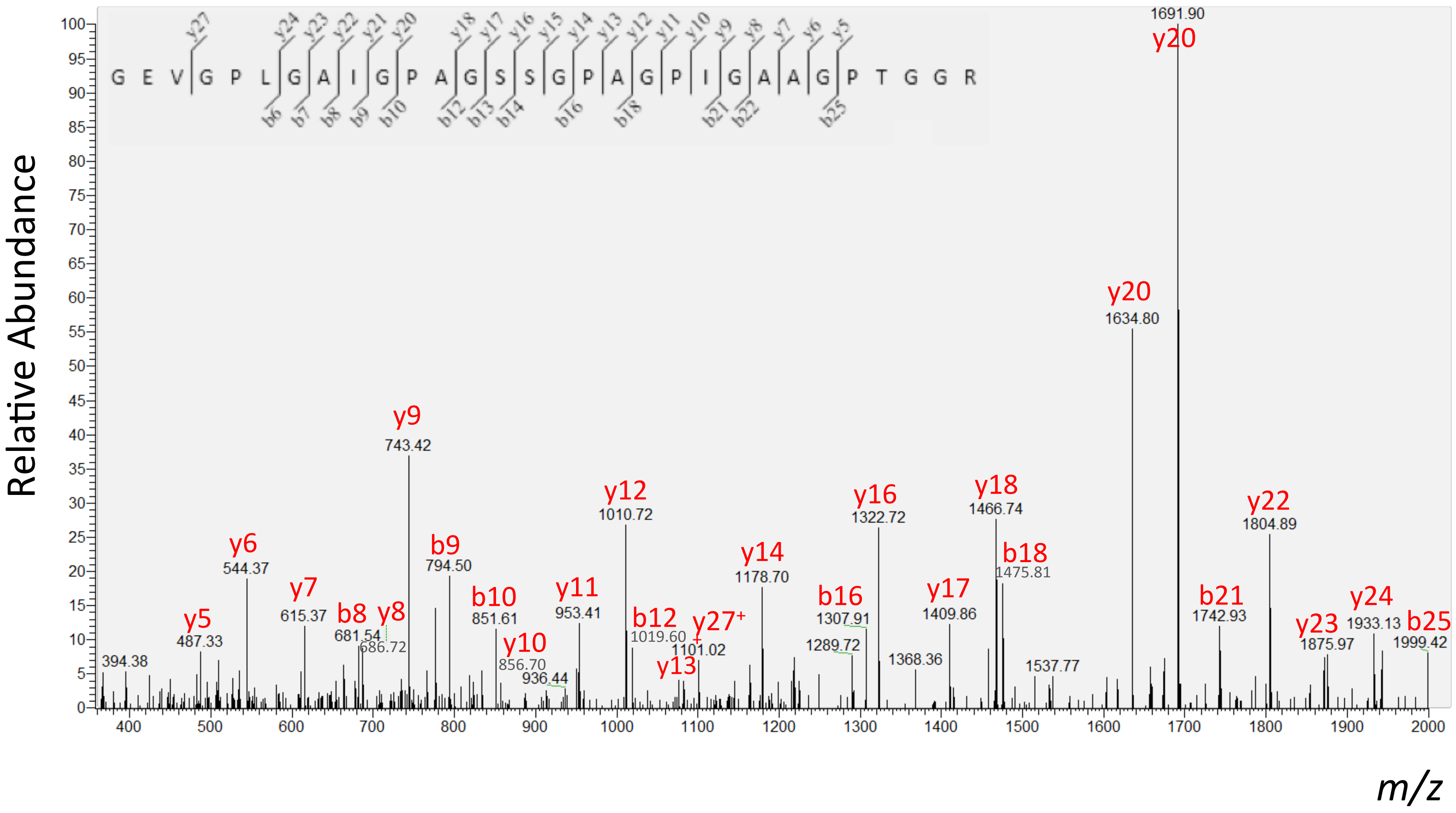

Supplementary Figure S2.19: Tandem mass spectrum for collagen (I) biomarker COL1A2T60 at *m/z* 2510 in *Dermochelys coriacea*.

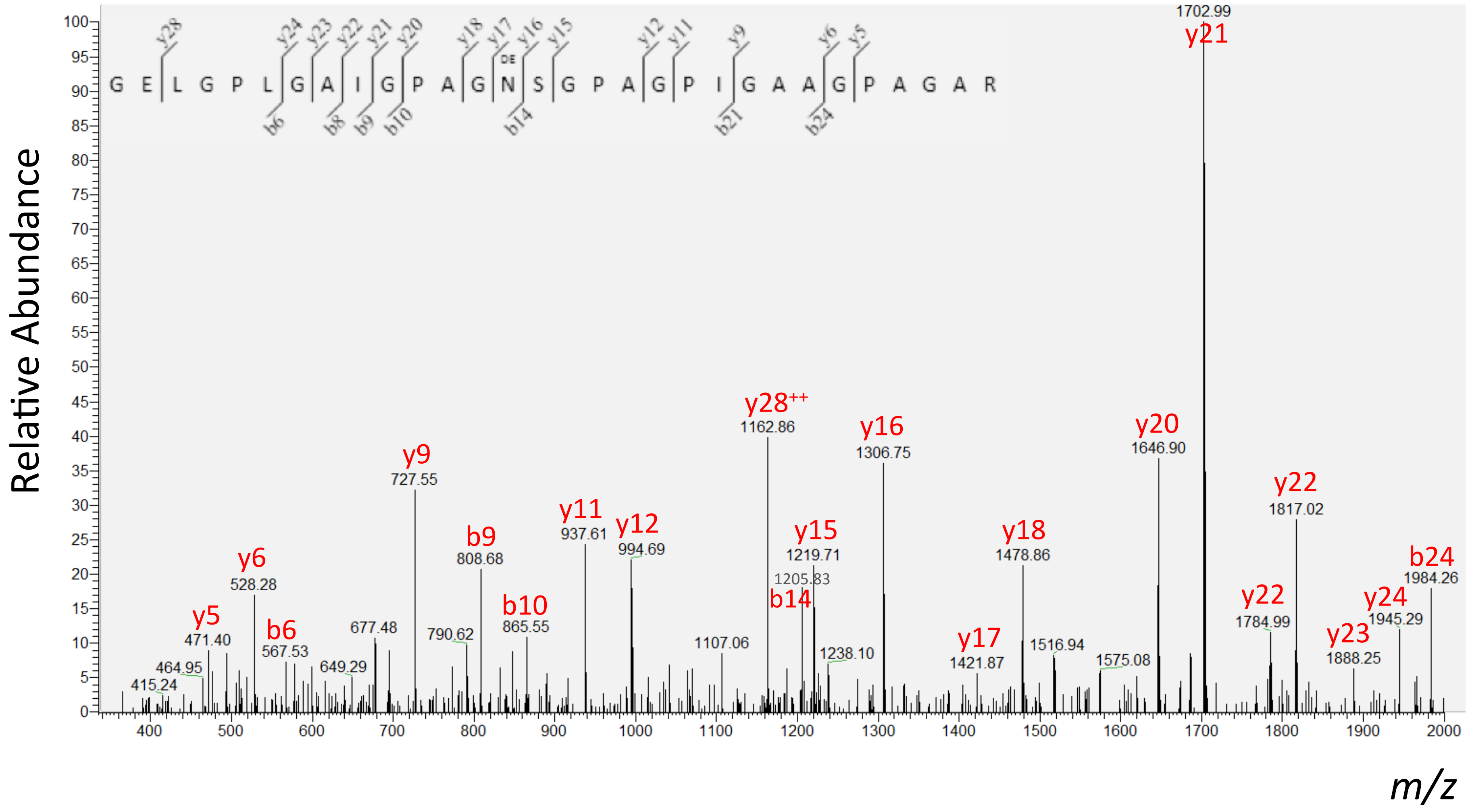

Supplementary Figure S2.20: Tandem mass spectrum for collagen (I) biomarker COL1A1T85 at *m/z* 2705 in all extant sea turtles.

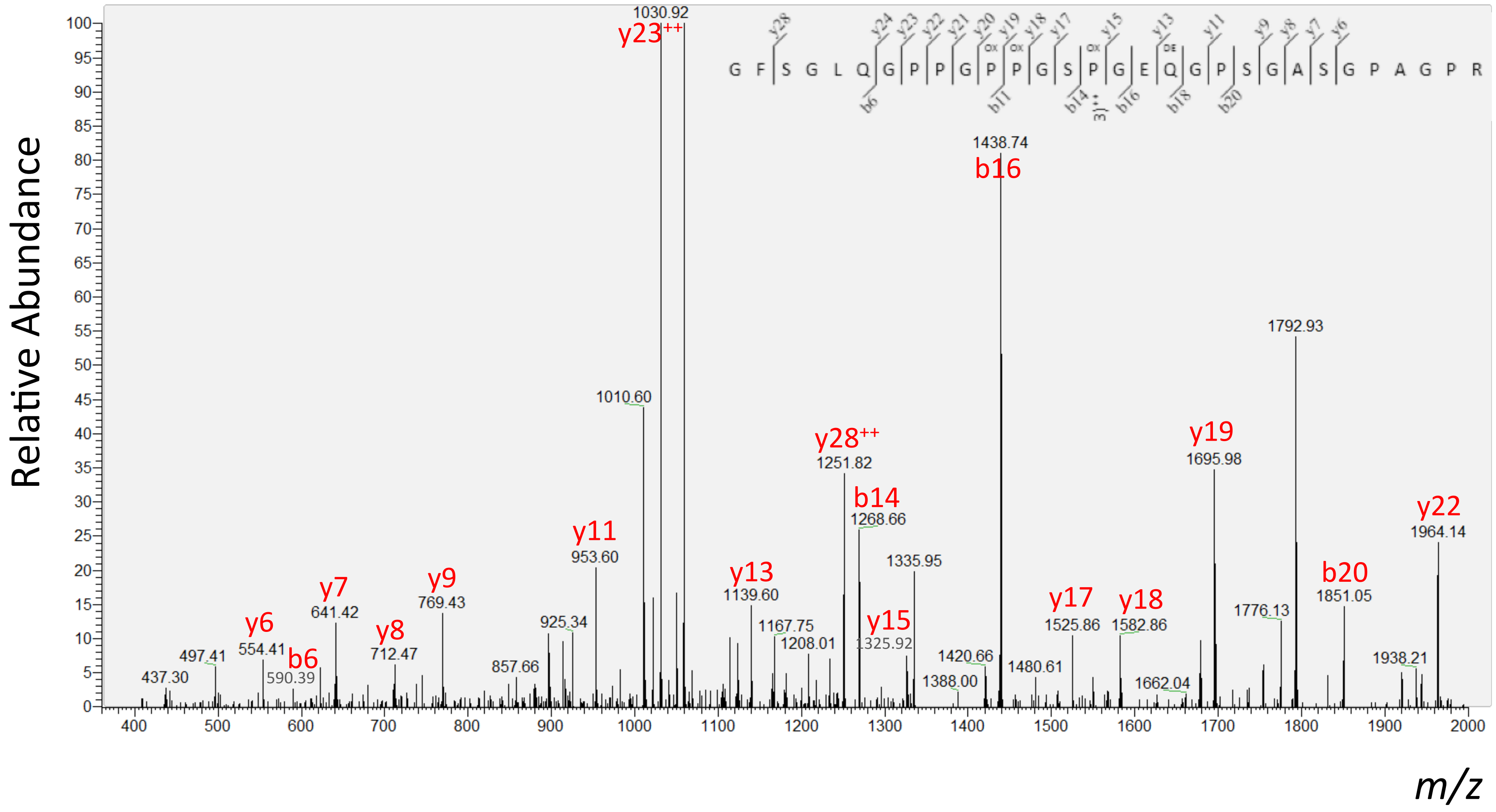

Supplementary Figure S2.21: Tandem mass spectrum for collagen (I) biomarker COL1A2T41/42 at *m/z* 2790 in all extant sea turtles.

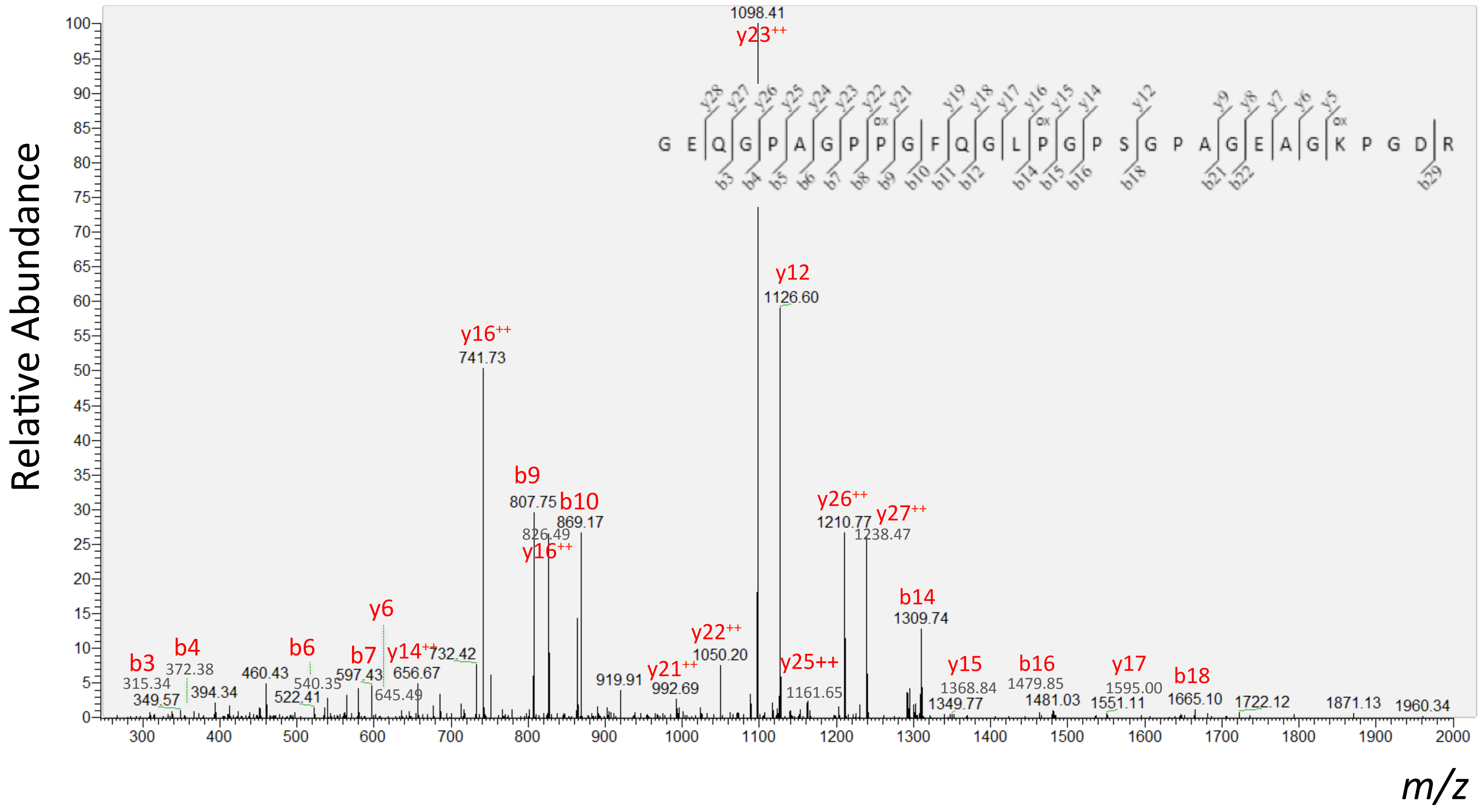

Supplementary Figure S2.22: Tandem mass spectrum for collagen (I) biomarker COL1A1T55/56 at *m/z* 2843/59 in *Chelonia mydas*.

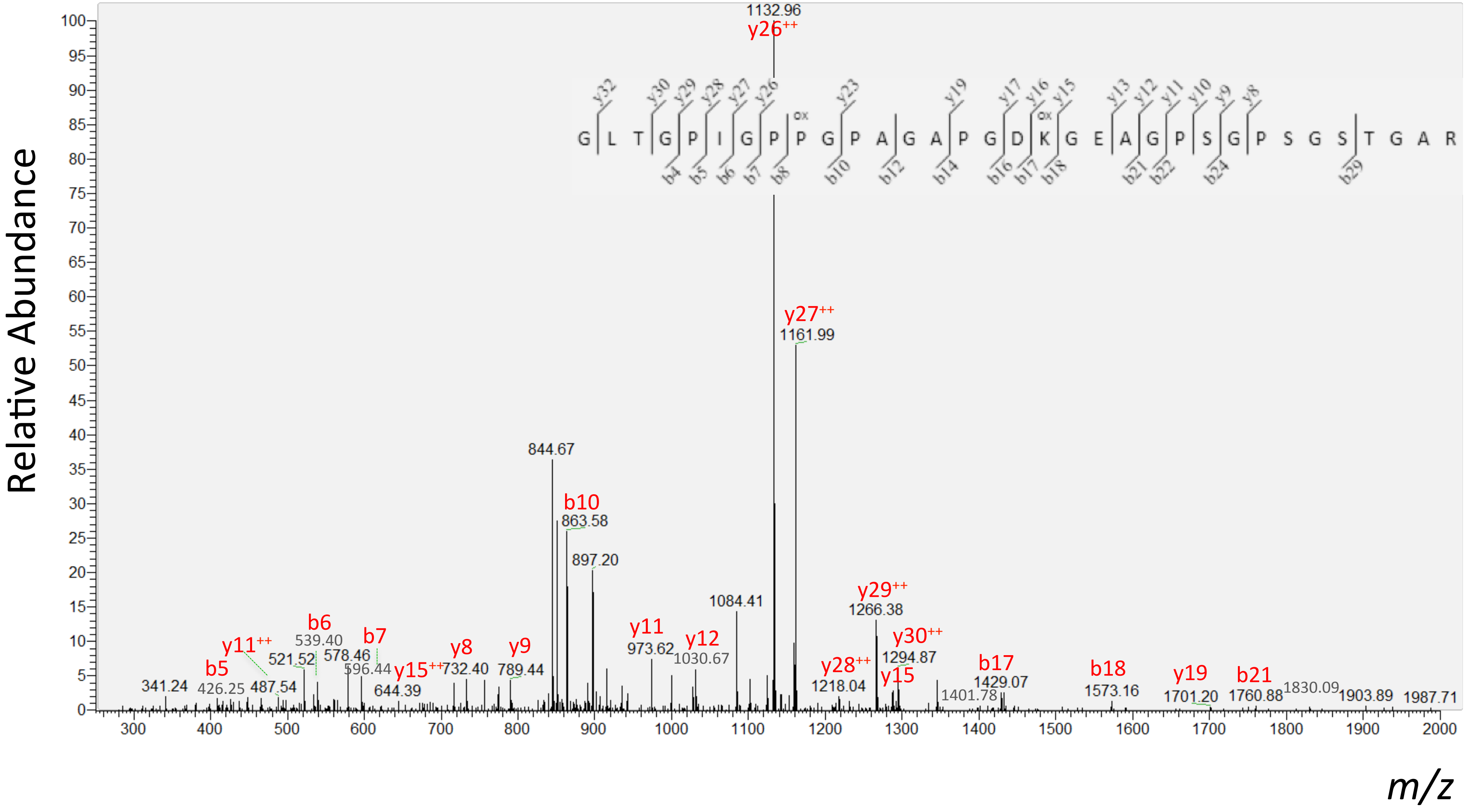

Supplementary Figure S2.23: Tandem mass spectrum for collagen (I) biomarker COL1A1T55/56 at *m/z* 2843/59 in *Eretmochelys imbricata*.

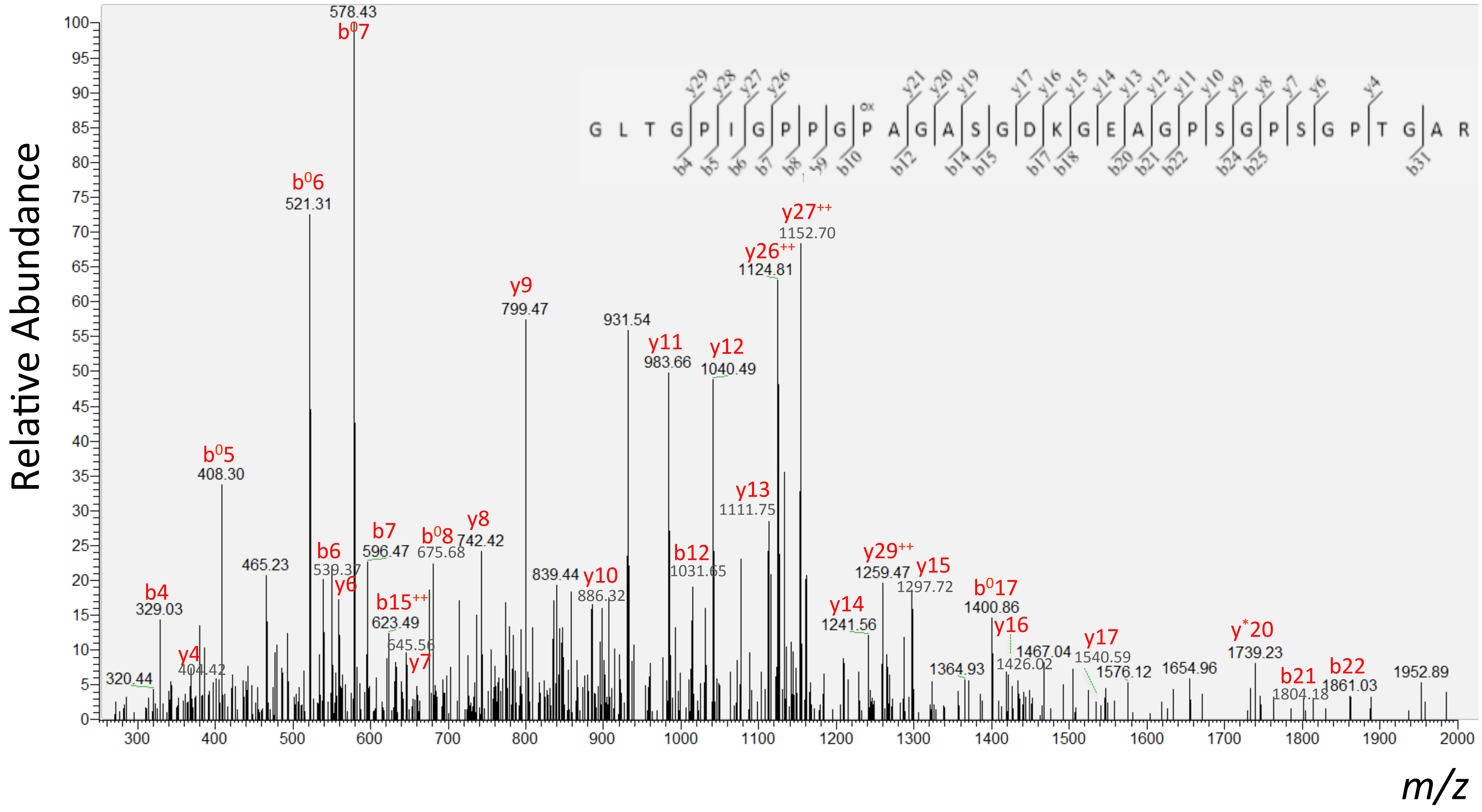

Supplementary Figure S2.24: Tandem mass spectrum for collagen (I) biomarker COL1A1T55/56 at *m/z* 2843/59 in *Lepidochelys kempii* and *L. olivacea*.

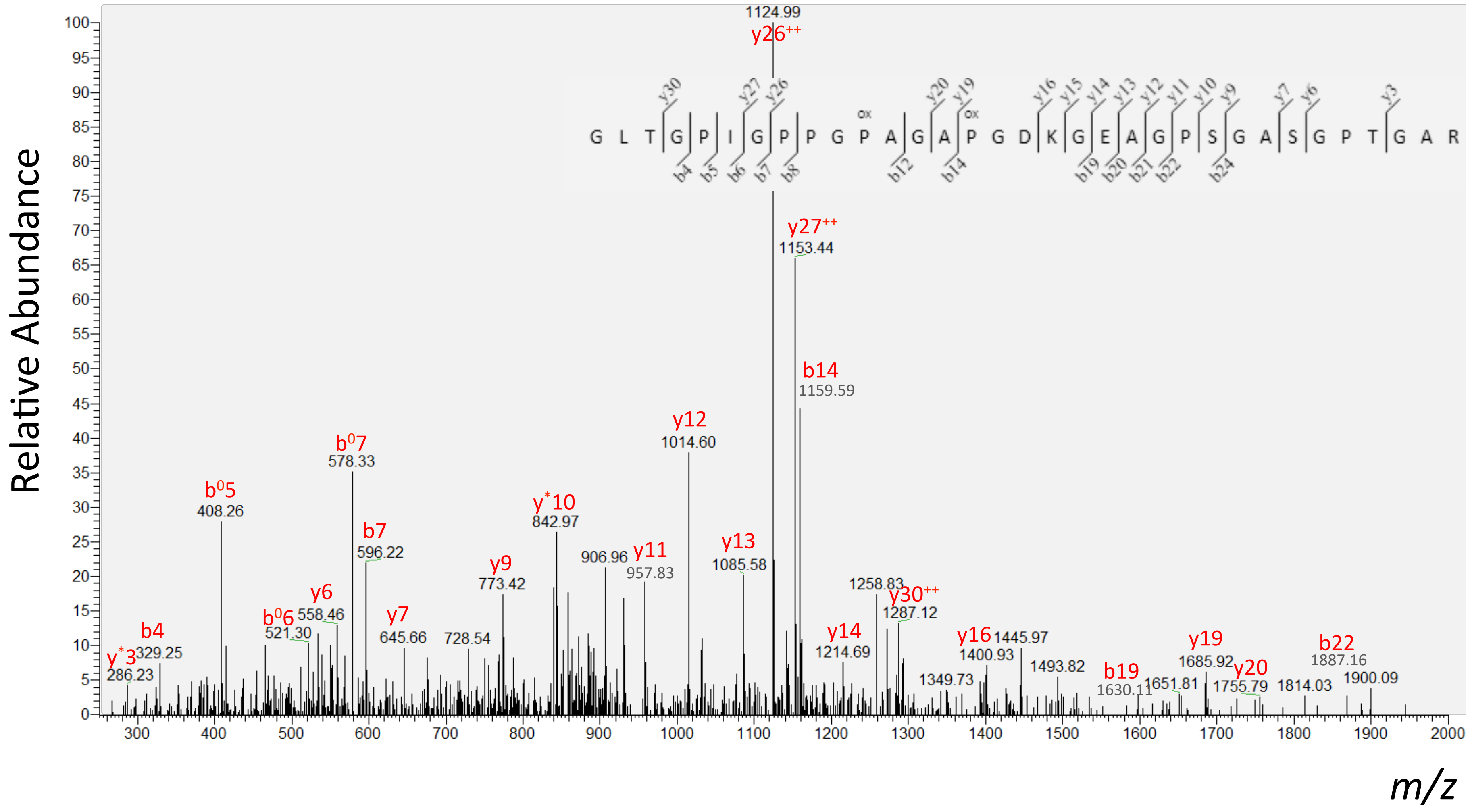

Supplementary Figure S2.25: Tandem mass spectrum for collagen (I) biomarker COL1A1T55/56 at *m/z* 2853/69 in *Dermochelys coriacea*.

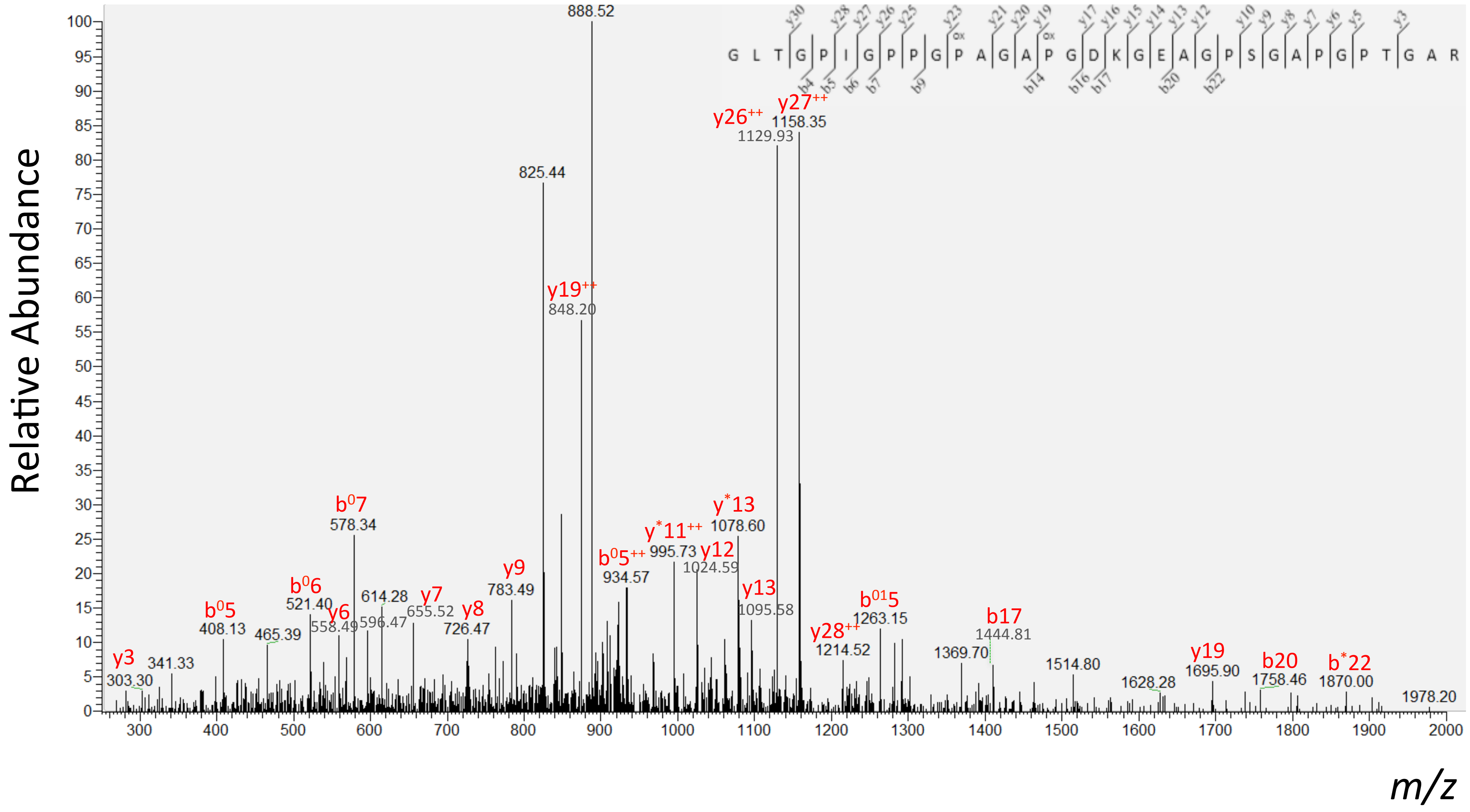

Supplementary Figure S2.26: Tandem mass spectrum for collagen (I) biomarker COL1A1T55/56 at *m/z* 2869/85 in *Natator depressus* and *Caretta caretta*.

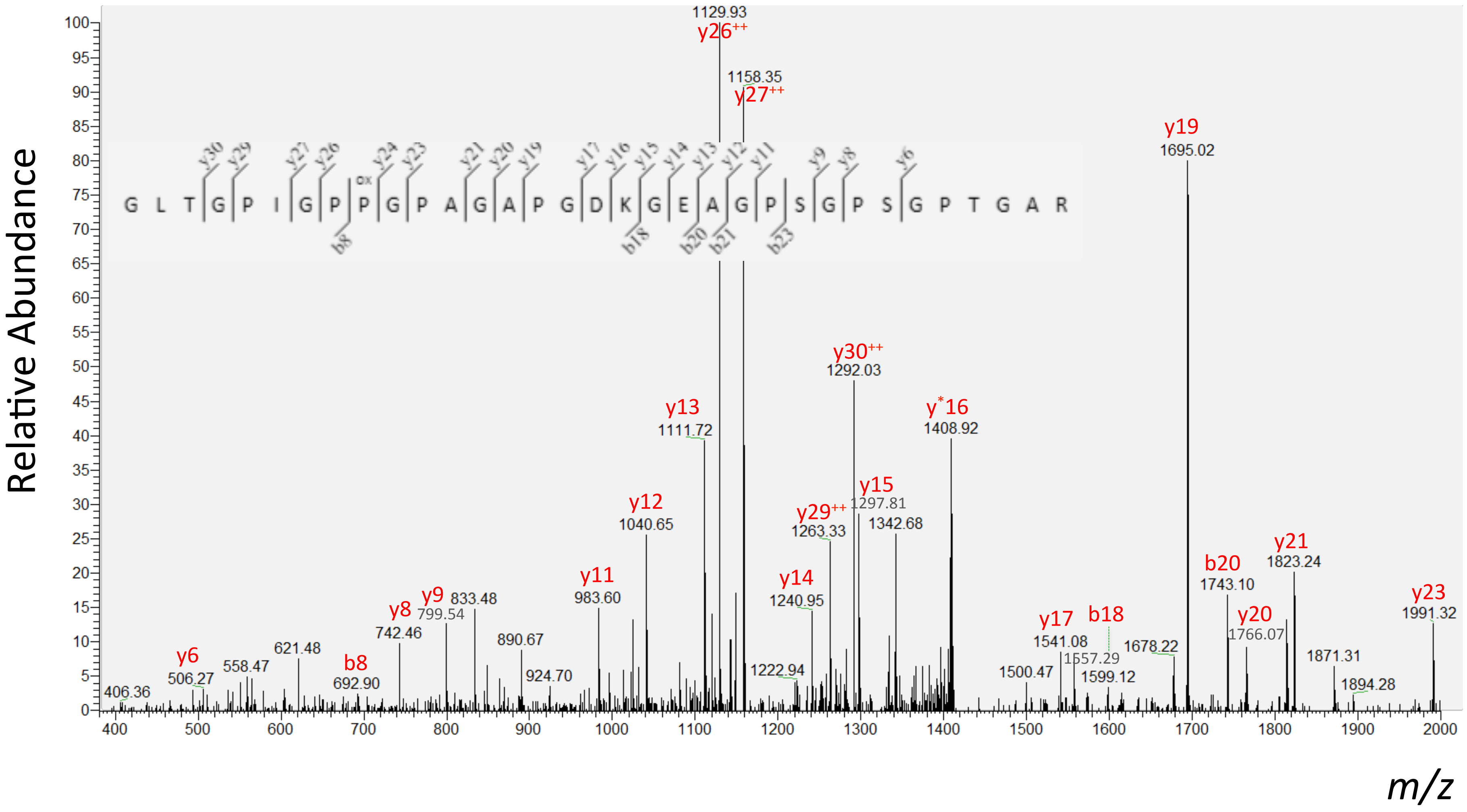

Supplementary Figure S2.27: Tandem mass spectrum for collagen (I) biomarker COL1A2T67 at *m/z* 2899 in *Dermochelys coriacea*.

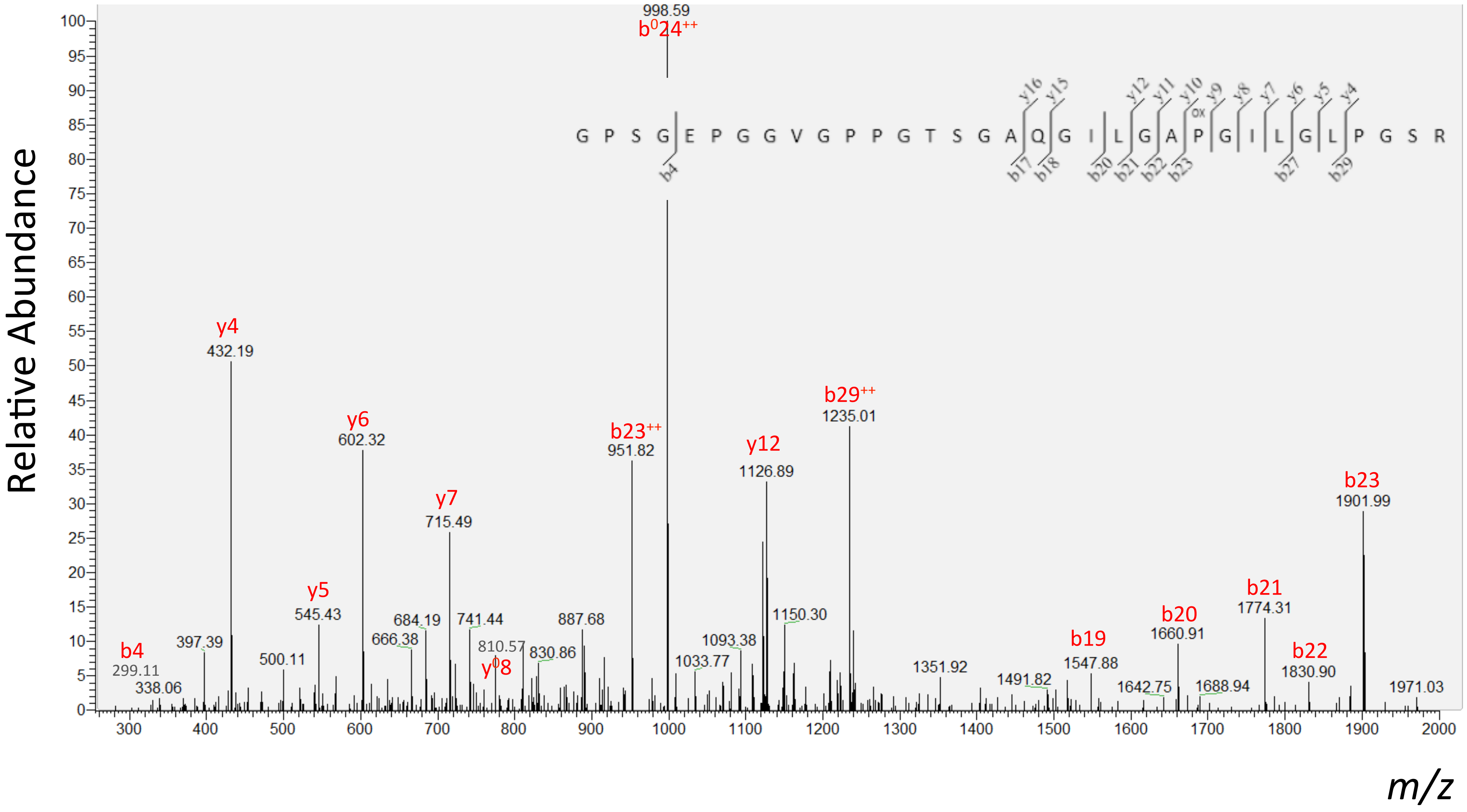

Supplementary Figure S2.28: Tandem mass spectrum for collagen (I) biomarker COL1A2T67 at *m/z* 2929 in *Chelonia mydas, Natator depressus, Caretta caretta, Eretmochelys imbricata, Lepidochelys kempii* and *L. olivacea*.

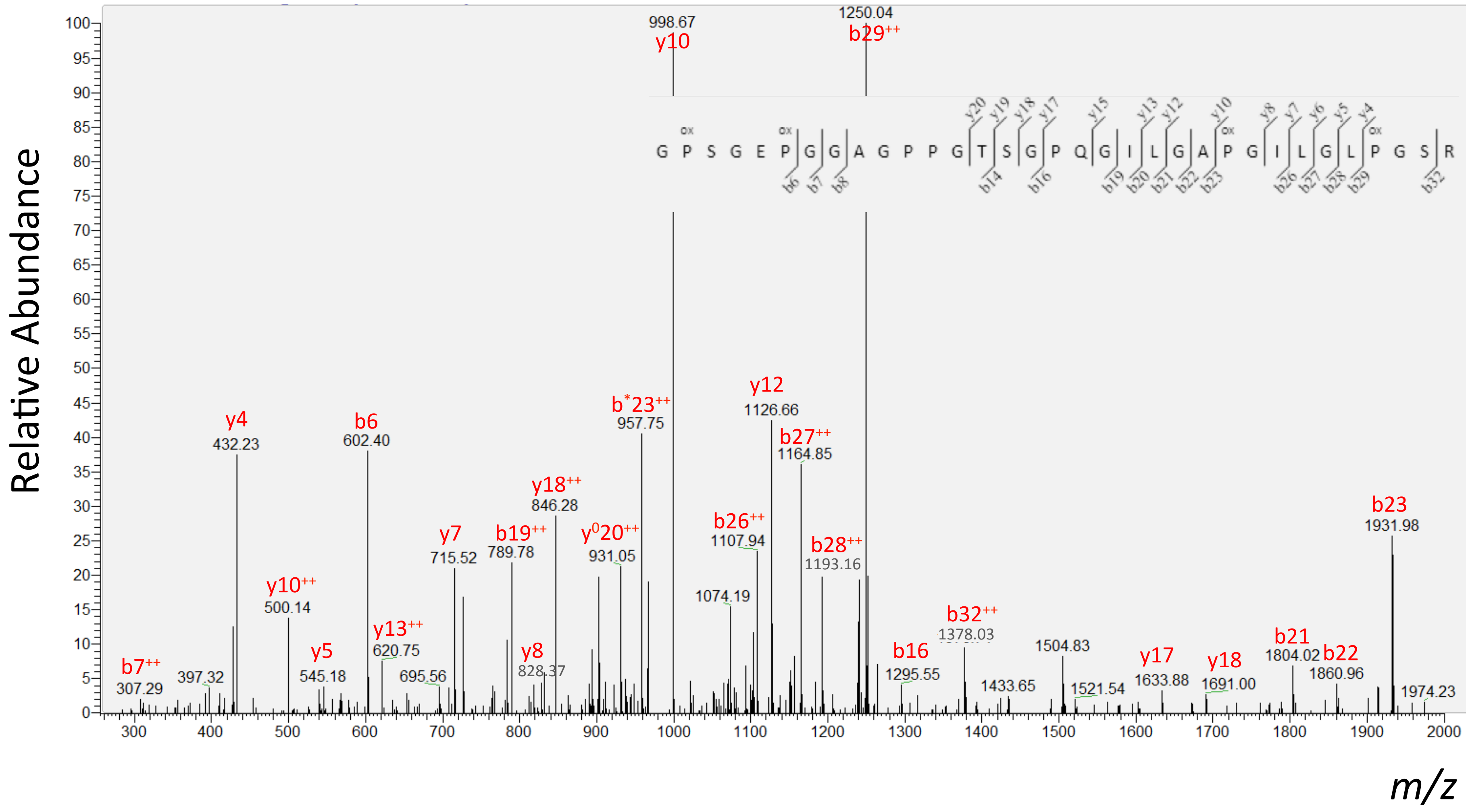

Supplementary Figure S2.29: Tandem mass spectrum for collagen (I) biomarker COL1A2T3 at *m/z* 3007 in *Chelonia mydas, Natator depressus, Caretta caretta, Eretmochelys imbricata, Lepidochelys kempii, L. olivacea* and *Dermochelys coriacea*.

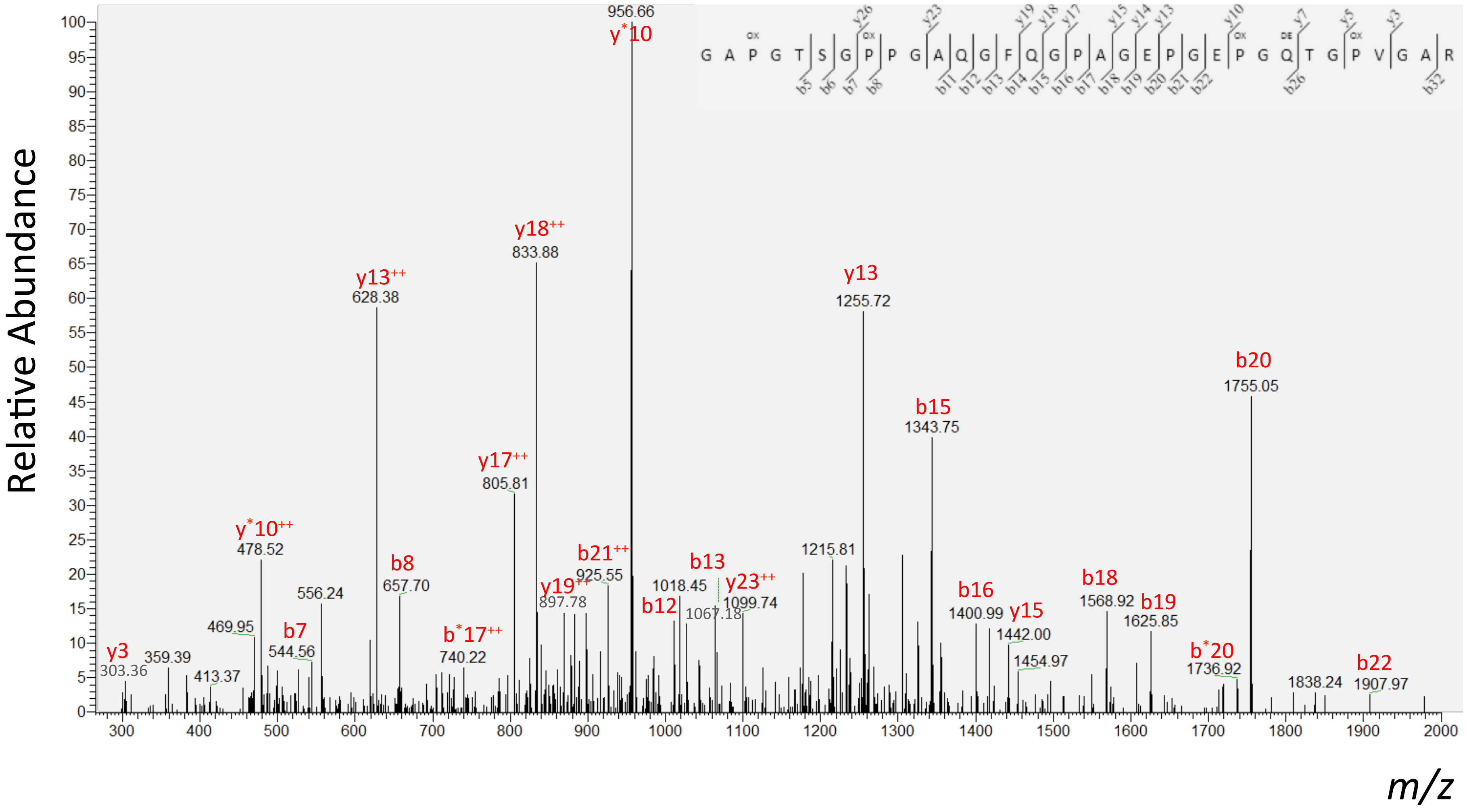

Supplementary Figure S2.30: Tandem mass spectrum for collagen (I) biomarker COL1A2T3 at *m/z* 3035 in *Chelonia mydas* (ancient samples GP9, GP20, GP56 and GB72 only).

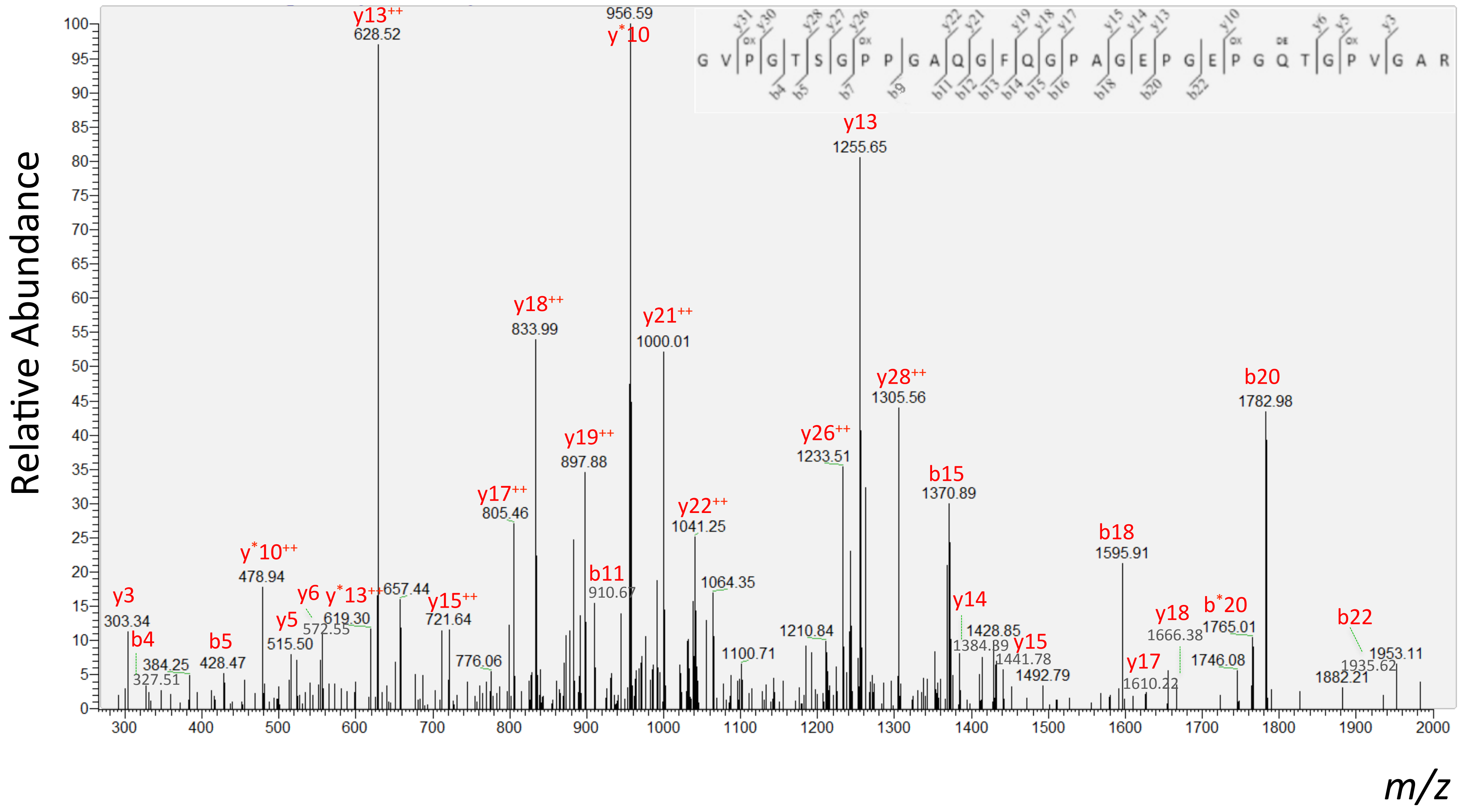

Supplementary Figure S3

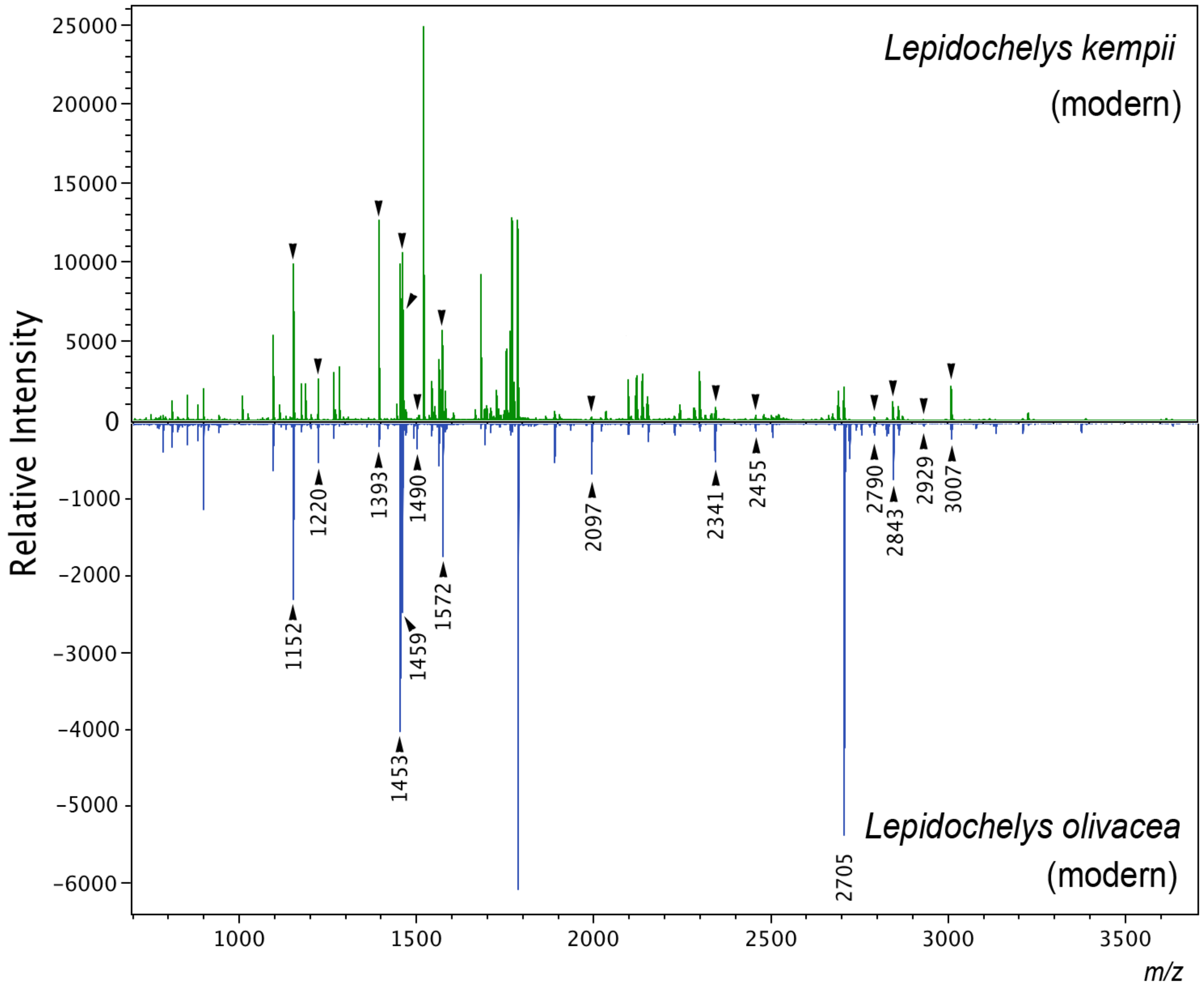

Supplementary Figure S3: Collagen fingerprints (10% and 50% combined fractions) for modern *Lepidochelys* species (top=Kemp's ridley, *L. kempii,* and bottom=olive ridley, *L. olivacea*), showing labelled biomarkers from Table 3.
